# Genome-Wide Evolution of Lineage-Specific Chromatin Accessibility Links Regulatory Changes to Cichlid Phenotypic Diversity

**DOI:** 10.1093/gbe/evag247

**Published:** 2026-10-08

**Authors:** Tarang K Mehta, Angela L Man, Graham Etherington, Alan M Smith, Adrian Indermaur, Walter Salzburger, Domino Joyce, Federica Di-Palma, Wilfried Haerty

**Affiliations:** Department of Biochemistry, Cell and Systems Biology, University of Liverpool, Liverpool L69 7BE, UK; Earlham Institute, Norwich NR4 7UZ, UK; Earlham Institute, Norwich NR4 7UZ, UK; Earlham Institute, Norwich NR4 7UZ, UK; Evolutionary and Ecological Genomics Group, School of Environmental and Life Sciences, University of Hull, Kingston Upon Hull HU6 7RX, UK; Department of Environmental Sciences, Zoological Institute, University of Basel, 4056 Basel, Switzerland; Department of Environmental Sciences, Zoological Institute, University of Basel, 4056 Basel, Switzerland; Evolutionary and Ecological Genomics Group, School of Environmental and Life Sciences, University of Hull, Kingston Upon Hull HU6 7RX, UK; Department of Medical Genetics, University of British Columbia, Vancouver V6H 3N1, Canada; Earlham Institute, Norwich NR4 7UZ, UK; School of Biological Sciences, University of East Anglia, Norwich NR4 7TJ, UK

**Keywords:** cichlid fishes, chromatin accessibility, epigenomics, gene regulatory networks, adaptive radiation, regulatory divergence

## Abstract

East African cichlids from Lakes Victoria, Malawi, and Tanganyika have undergone explosive adaptive radiation, generating extensive morphological, ecological, and behavioral diversity within a few million years. Understanding how genome-wide regulatory evolution contributes to this diversification requires comparative analyses of chromatin accessibility, transcription factor (TF) binding, and gene regulatory networks across species and tissues. Here, we combine high-coverage ATAC-seq with matched RNA-seq in forebrain, retina, liver, and testis from five East African cichlid species spanning radiating and nonradiating lineages to characterize the evolution of regulatory landscapes in this radiation. Thousands of promoter-associated accessible sites are conserved across species and tissues, yet more than half of these shared promoters show signatures of accelerated nucleotide evolution, particularly in genes linked to development and sensory function. In contrast, smaller sets of lineage- and tissue-specific accessible promoters, especially in retina, exhibit strong regulatory divergence and functional enrichment for visual system processes. Integrating TF footprinting and expression data uncovers pervasive TF binding site (TFBS) turnover at active promoters and extensive, tissue-dependent rewiring of motif-supported gene regulatory networks, with 86% to 97% of high-confidence edges being species-specific. Extending promoter TFBS variants into broader Malawi and tilapia phylogenies shows that functional variants in promoters of visual genes, such as *actr1*, segregate by clade and ecological niche. Together, these results demonstrate how accelerated regulatory sequence evolution, chromatin accessibility divergence, and TFBS turnover jointly reshape regulatory architecture genome-wide in an iconic vertebrate radiation, providing insights into how noncoding regulatory evolution can drive rapid phenotypic diversification.

## Graphical Abstract

**Graphical Abstract:**
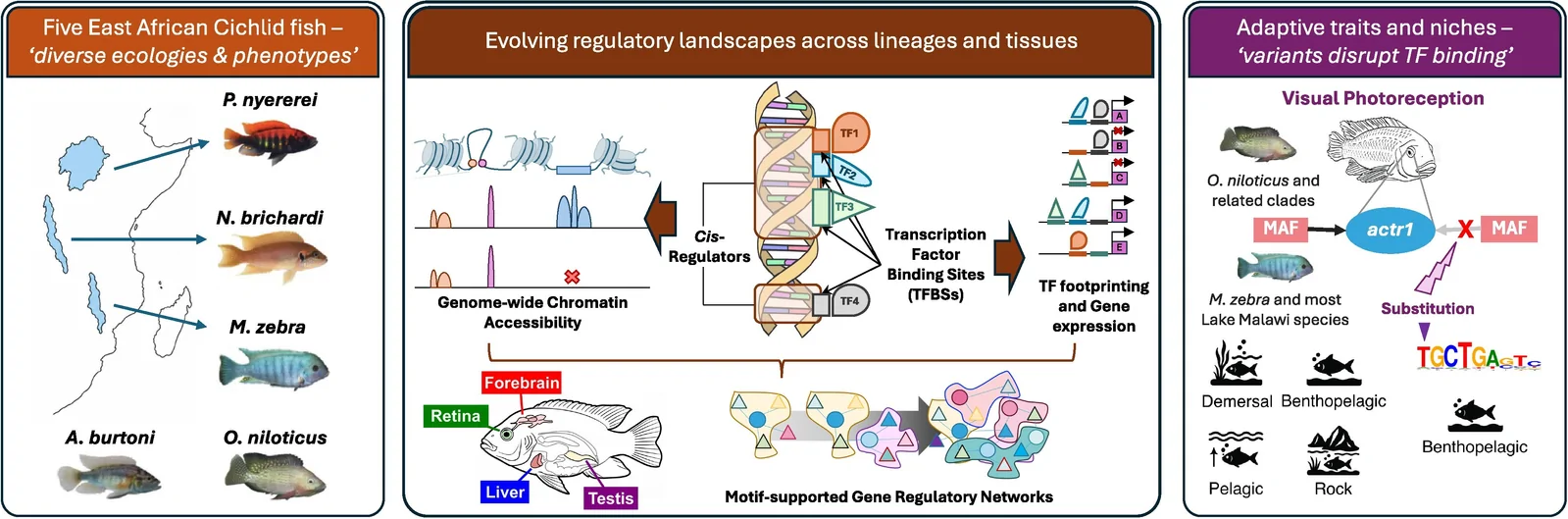

Significance Cichlid fishes have evolved an extraordinary range of colors, body shapes, and ways of life in a very short time, but we still know relatively little about how changes in gene regulation across the genome help to generate this diversity. This study uses genome-wide maps of regions open for transcription (chromatin accessibility) and gene activity in multiple tissues and species to show that many regulatory “switches” are both deeply conserved and rapidly changing, and that small DNA changes in these regions can alter when and where key genes are turned on. By linking these regulatory changes to genes associated with key traits that help cichlids adapt to diverse environments, such as vision, this work shows how evolution of the genome's regulatory control layer can drive rapid phenotypic diversification in natural vertebrate populations.

## Background

Seminal studies analyzing vertebrate protein evolution proposed that evolutionary changes in “regulatory systems” are a key driver of morphological diversity (Britten and Davidson 1969; Wilson et al. 1974; King and Wilson 1975; Prager and Wilson 1975). These systems comprise cis-regulatory elements (CREs) such as promoters, enhancers, and silencers, which act combinatorially and harbor transcription factor binding sites (TFBSs) that spatiotemporally control gene expression during development (Wittkopp and Kalay 2011). While several locus-specific studies have demonstrated the role of CRE evolution in morphological change (Gompel et al. 2005; Jeong et al. 2006; Prud'homme et al. 2006; Cretekos et al. 2008; Chan et al. 2010), novel sequencing methods now provide genome-wide, though indirect, views of chromatin accessibility and TF binding (Klemm et al. 2019). The assay for transposase-accessible chromatin using sequencing (ATAC-seq) is particularly powerful due to its speed and simplicity, enabling chromatin accessibility profiling from ultra-low input (Buenrostro et al. 2013) or even single cells (Buenrostro et al. 2015). Beyond insights into mammalian CRE evolution (Kuderna et al. 2024), ATAC-seq enables assessment of relative chromatin-accessibility divergence at candidate CREs in adaptive radiations (Roscito et al. 2018; Uesaka et al. 2019; Yuan et al. 2021; Tetrault et al. 2023).

Previous work in vertebrates has shown that evolution of noncoding regulatory elements and rewiring of gene regulatory networks (GRNs) can underlie rapid phenotypic change (King and Wilson 1975; Wray 2007; Carroll 2008). Cichlid fishes, with their explosive diversification in East African lakes (Salzburger 2018), provide a powerful model to investigate how such regulatory processes operate across multiple tissues and lineages in a natural adaptive radiation. Within the past few million years (Genner et al. 2007; Wagner et al. 2012), one or a few ancestral lineages gave rise to more than 2,000 cichlid fish species in the African Great Lakes (Victoria, Malawi, and Tanganyika). These lineages occupy diverse ecological niches, exhibiting extensive phenotypic diversity in coloration, skeletal morphology, trophic structures, and behavior (Hulsey 2009). Previous studies have demonstrated that the rapid evolution of regulatory elements (Brawand et al. 2014) and regulatory changes driving GRN rewiring (Mehta et al. 2022) have contributed to cichlid diversification. Other work has shown evolutionary changes in noncoding regulatory regions in the Lake Victoria radiation (Brawand et al. 2014; Urban et al. 2021) and linked gene expression divergence to speciation and trait diversification in Lake Tanganyika (El Taher et al. 2021; Fages et al. 2026).

Although still relatively unexplored, epigenomic studies in cichlids have begun to illuminate regulatory evolution. Initial work mapped active promoters in *Oreochromis niloticus* fin development and regeneration using H3K4me3 ChIP-seq (Kratochwil and Meyer 2015), and our recent work integrated ATAC-seq and RNA-seq analyses to identify regulatory loci underlying salinity and osmotic stress acclimation in tilapia (Mehta et al. 2023). Other studies have connected cis-regulatory divergence with dietary ecology in Lake Malawi cichlids (Vernaz et al. 2021), speciation processes (Vernaz et al. 2022), and sexual dimorphism in tilapia (Wan et al. 2016), as well as jaw shape variation to regulatory divergence in Malawi species (Tetrault et al. 2023). However, no study has yet compared cis-regulatory sequence evolution with tissue- and lineage-specific chromatin accessibility and transcription across multiple African cichlid lineages. Here, we present the first genome-wide, multitissue comparative analysis of chromatin accessibility and cis-regulatory sequence evolution across multiple East African cichlid species to assess three key questions: (i) how chromatin accessibility and TF binding landscapes evolve across tissues and species; (ii) how nucleotide changes in active regulatory DNA relate to TFBS turnover and GRN rewiring; and (iii) how these regulatory changes connect to adaptive traits in this iconic vertebrate radiation.

## Results

### Chromatin Accessibility Maps Regulatory Architecture Across Tissues

To characterize variation in chromatin accessibility associated with phenotypic diversity in cichlid fishes, we performed ATAC-seq on four tissues that link to traits under natural/sexual selection—forebrain, retina, liver, and testis—from five representative East African cichlids used in comparative studies (Brawand et al. 2014) and spanning the major haplochromine radiation and the more distantly related lamprologine lineage: *Metriaclima zebra* (Lake Malawi)*, Pundamilia nyererei* (Lake Victoria)*, Astatotilapia burtoni* (Lake Tanganyika associated, but also inhabiting multiple rivers) from the haplochromine radiation, *Neolamprologus brichardi* from the lamprologine lineage of Lake Tanganyika, and *O. niloticus* as a riverine and lacustrine freshwater reference species (Fig. 1a and Table S1; see “*Methods*”). This dataset enabled genome-wide annotation and comparison of conserved and species specific open chromatin regions across the phylogeny (Fig. 1d). Across all species and tissues—using consensus peaks that overlap a gene's upto 5 kb promoter region across replicates (see “*Methods*”)—78% to 99% had a summit within 5 kb of that gene's transcription start site (TSS), and 10% to 20% of these peaks fell within the core promoter region [±100 bp (Roeder 1996) of the TSS] (Fig. 1b and c, Figs. S1 and S2, Tables S2 and S3; see “*Supplementary information*”). Separately, genome-wide annotation of all consensus peaks to one dominant feature revealed that across all five species, peaks overlapped intronic regions most frequently (40%), followed by exons (23.5%) and intergenic regions (17.4%), with smaller fractions assigned to 5′ UTRs (6.5%), promoter regions within 5 kb upstream of the TSS (10%), 3′ UTRs (2.5%), and conserved noncoding elements (0.1%) (Fig. S2, Table S3). This overall distribution is broadly consistent with profiles reported from large-scale human ATAC-seq datasets (Smith et al. 2021), indicating that East African cichlids share a conserved regulatory architecture in which promoters and intragenic elements are major hubs of chromatin accessibility. This architecture provides a basis on which regulatory changes can accumulate across tissues and species. This shared architecture forms the common backbone against which the lineage- and tissue-specific differences in accessibility described in the following sections can be assessed.

**Fig. 1.**
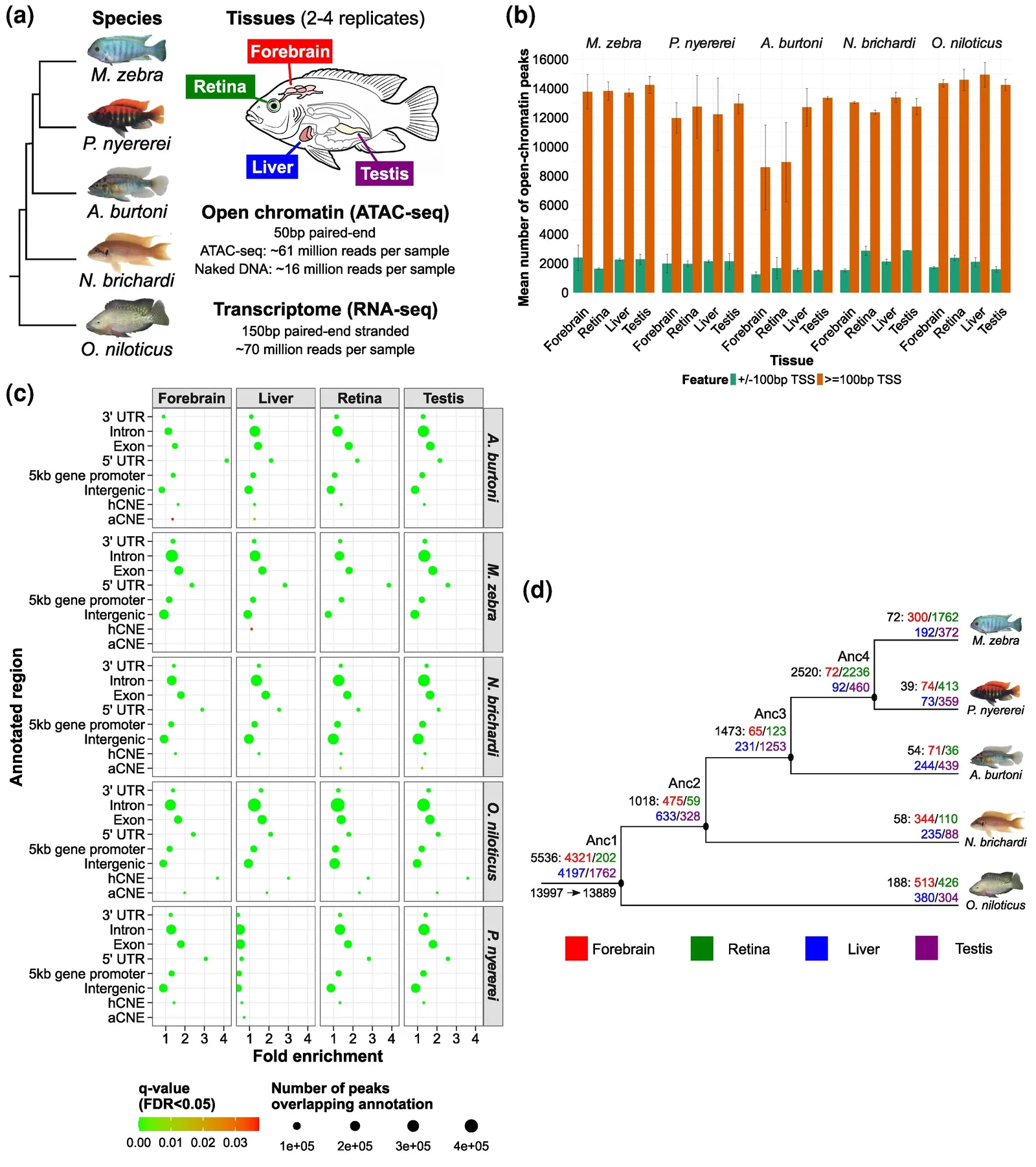
The open chromatin landscape of five cichlid species. (A) Summary of the species, tissues, and sequencing design of this study. See ‘Methods’ and Supplementary Table S1. (B) Mean number of open chromatin peaks across the five species and four tissues with standard deviation (error bars), separated by peaks that are located either +/- 100 bp from a Transcription Start Site (TSS) or >=100 bp from a TSS. (C) Fold enrichment of tissue-specific open chromatin peaks overlapping coding and noncoding regions in the five cichlid genomes. Circles show enriched annotated region (y-axis) of significance (FDR <0.05, to right) and fold enrichment (x-axis) values of all annotated peaks. Number of peaks overlapping each annotation shown by size of each circle. (D) Number of 1-to-1 orthologous gene with associated open chromatin peaks (overlapping up to 5kb gene promoter) shown along the phylogeny, indicating in black the count of orthologous genes with positionally conserved peaks at ancestral nodes or those specific to each species, and in red, green, blue, or purple the count present in forebrain, retina, liver, or testis, respectively.

### Core Regulatory Elements Underpin Shared and Tissue-specific Functions

To define the core promoter-accessibility architecture shaping cichlid diversity, we mapped promoter open chromatin peaks of species-tissue replicates to 13,889 1-to-1 orthologous genes across forebrain, retina, liver, and testis of five species, revealing an enrichment of broadly shared processes such as epithelium development together with tissue-specific functions including synapse organization (forebrain), axon extension (retina), myotube differentiation (liver), and dephosphorylation (testis) (Fig. 1d; Fig. S3; *see* “*Methods*”). We first examined positionally “conserved” promoter peaks, defined as open chromatin peaks with overlapping summits across species (see “*Methods*”). In total, we identified 5,536 orthologous genes, representing 40% of all 13,889 1-to-1 orthologous genes, with such positionally conserved promoter peaks shared across all five species within the same tissue (202 to 4,321 genes per tissue), indicating positionally maintained regulatory elements along the phylogeny (“conserved” at ancestral node 1, *Anc1*, Fig. 1d; see “*Methods*”). These genes are enriched for fundamental biological processes such as “cell migration” (Fig. S4; Table S4) and include key developmental and housekeeping regulators, for example *irx2*, a core vertebrate neural differentiation gene (Cohen et al. 2000). Within *P. nyererei*, *M. zebra,* and *A. burtoni* (haplochromine species), we identified 1,473 nonredundant genes (65 to 1,253 orthologous genes per tissue) with positionally conserved promoter peaks across tissues (“conserved” in haplochromine ancestor—ancestral node 3, *Anc3*, Fig. 1d). Gene ontology analysis linked these positionally conserved peaks to core vertebrate functions such as sensory system development (Fig. S4; Table S4) and to promoter activity of fundamental genes, for example *pax6*, a master regulator of eye development in all seeing animals (Gehring and Ikeo 1999). Together, these evolutionarily maintained candidate core regulatory elements could underpin shared biological functions and key aspects of cichlid development. Further, the 1,473 genes with clade-specific conserved promoter peaks in the haplochromine ancestor (Anc3) indicate positionally conserved regulatory elements that either predate or were gained early in, the haplochromine radiation. This combination of a conserved regulatory core and flexible, lineage-specific elements illustrates how gene promoter accessibility could support both stability and innovation in cichlid regulatory systems.

### Conserved and Species-specific Promoter Accessibility Reveals Tissue-specific Regulatory Variation

We next explored unique patterns of chromatin accessibility that distinguish species and tissues by classifying nonoverlapping promoter peaks as “unique”, that is, peaks whose summits were not encompassed within a shared interval between species (see “*Methods*”). Across all five species and four tissues, species-specific promoter peaks were associated with a total of only 39 to 188 nonredundant orthologous genes (Fig. 1d). However, these “unique” peaks likely contribute to regulatory diversity, especially in the retina, where the five species together showed a total of 2,747 nonredundant orthologous genes with unique gene promoter peaks compared with 1,124 to 1,562 in the other tissues (Fig. 1d). Unique peaks also showed high variability at both the ancestral and species level (ancestral coefficient of variation CV = 162%, species-specific CV = 122%; Fig. 2a; see “*Supplementary information*”). Notably, the retina exhibits the fewest positionally “conserved” promoter peaks in only 202 orthologous genes (*Anc1* node, Fig. 1d, i.e. peaks shared by all five species in retina specifically), representing only 1.8% to 2.9% of the 6,981 to 11,382 1-to-1 orthologous genes that are expressed in retina tissue across the five species. This high variation in chromatin accessibility supports previous findings of dynamic, lineage- and clade-specific rewiring of visual system regulatory networks in the same species (Mehta et al. 2021, 2022).

**Fig. 2.**
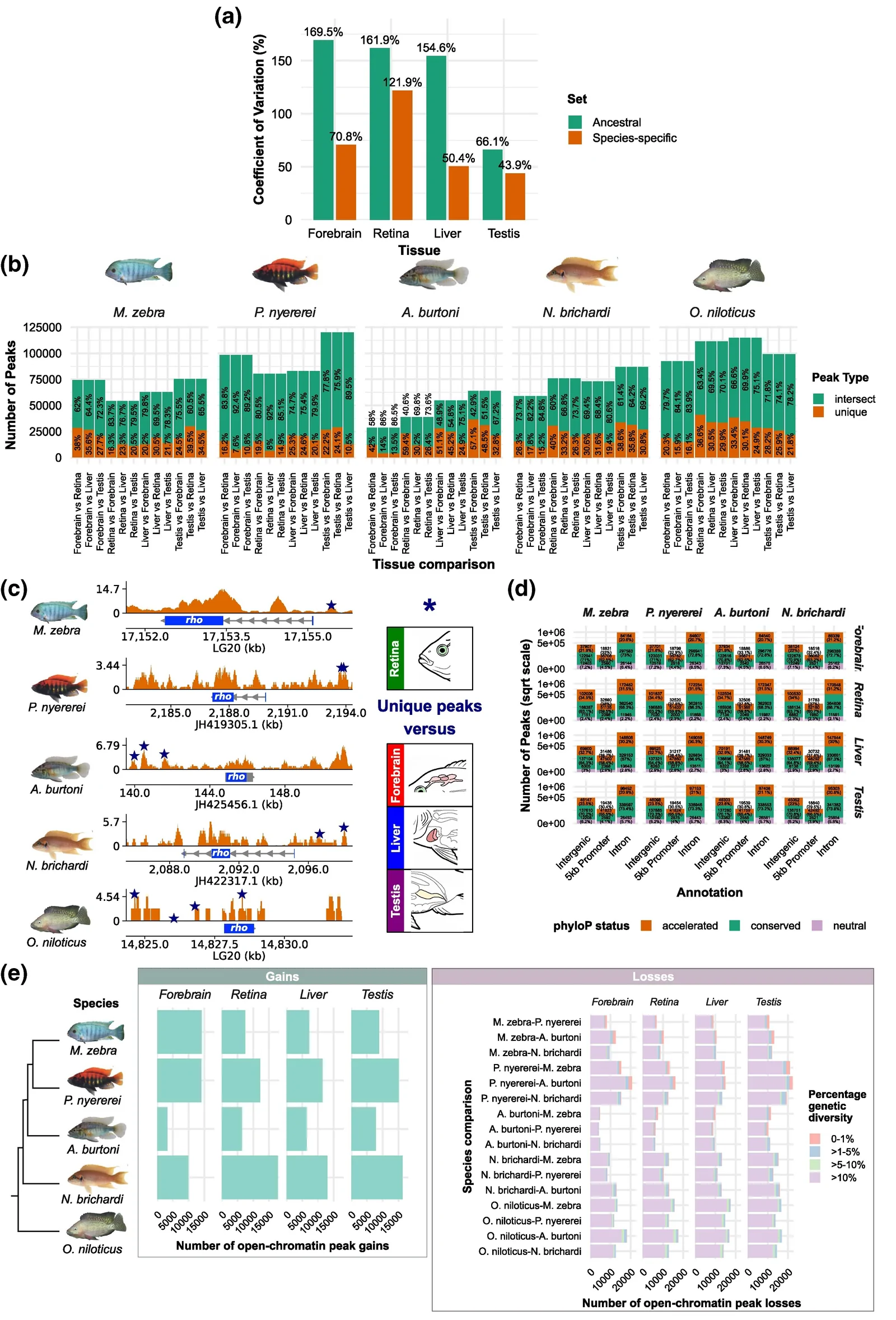
Evolution of gene promoter peaks across species and tissues. a) CV in the number of promoter peaks in orthologous genes per tissue, comparing the ancestral node of all species (Anc1 in Fig. 1d; green bars) with species-specific sets (orange bars); CV values shown above each bar. b) Number and percentage of shared and unique gene promoter peaks between all pairwise tissue comparisons within species. Peaks were aggregated using the highest −log10 q-value for fully overlapping replicates, with each reference tissue peak counted once if overlapping, resulting in asymmetry for reverse comparisons, where a single reference peak overlapping *n* comparison peaks is counted *n* times (see Table S5). c) Retina-specific peaks (navy stars) versus other three tissues in the rho gene promoter region of each species. d) Stacked plot (square root scale) of phyloP-classified conserved, neutral, and accelerated peaks found in intergenic, promoter, or intronic regions across four species and tissues, using *O. niloticus* as the reference. True peak counts and percentages of total peaks per annotation are indicated within bar stacks; low-count annotations (UTRs, exons, CNEs) omitted for clarity and presented in Table S6. e) Number of open chromatin peak gains and losses in 1-to-1 orthologous gene promoters, with species-specific gains and pairwise losses determined by lack of overlapping peaks in promoter alignments. Pairwise losses are binned by percentage genetic diversity (0% to 1%, >1% to 5%, >5% to 10%, >10%) between the reference and aligned region in the other species.

To further dissect regulatory specificity, we performed pairwise comparisons of chromatin peaks between tissues within each species, defining promoter peaks as “conserved” only when both tissues' peak summits fell within the shared interval and as “unique” otherwise. Promoter peaks showed high conservation across all 60 tissue–tissue comparisons (mean 72.7% shared ± 11.3 SD, range 41% to 92%; Table S5). Retina tissue showed the numerically highest, though not statistically significant, mean proportion of unique peaks across all pairwise comparisons (mean 29.8% ± 10.9 SD, *n* = 30; range 8% to 59%), the highest among all tissues (forebrain 27.3%, liver 26%, and testis 26.2%), with no significant difference across tissues confirmed by ANOVA (*P* = 0.55; Table S5). Nonetheless, this overall prevalence of retina-specific chromatin peaks mirrors findings in the human retina, where sensory tissues exhibit distinct and highly dynamic accessible regions (Xie et al. 2020; Liang et al. 2023), and the identification of ∼41% retinal-specific peaks in retina and retinal pigmented epithelium comparisons (Wang et al. 2018). Conserved peaks across pairwise tissue comparisons identify genes implicated in shared tissue functions—such as axonal guidance, e.g. *nrp1a* (Bloomquist et al. 2017) in forebrain/retina of all species—while unique peaks are often associated with tissue-specific genes, e.g. the dim-light vision gene *rhodopsin* (*rho*) in the retina of all species (Fig. 2c). These findings indicate that, while a core of conserved regulatory elements is important for shared functions, tissue-specific peaks could provide a major axis of regulatory flexibility. In cichlids, this flexibility is particularly evident in sensory tissues such as the retina, where a conserved core cis-regulatory architecture provides the background upon which lineage-specific regulatory innovations can accumulate (Ogawa et al. 2025).

### Accelerated Nucleotide Evolution is Prevalent at Accessible Gene Promoters in Cichlids

To investigate the scale of gene regulatory evolution along the cichlid phylogeny, noting that elevated substitution rates can arise from relaxed constraint, biased gene conversion, or mutation-rate variation as well as positive selection, we calculated rates of nucleotide substitutions (Pollard et al. 2010) at gene promoter chromatin peaks, using the phylogenetic outgroup species, *O. niloticus*, as an unbiased reference for comparisons with the four other species (see “*Methods*”). Notably, relative to other noncoding regions (intergenic and intronic), gene promoters harbored a significantly higher proportion of accelerated peaks than exons in every comparison and higher than introns in most pairwise species comparisons (Wilcoxon rank-sum test, BH-adjusted; Table S7). Promoters also showed the numerically highest proportion (30% to 39%) of open chromatin peaks undergoing accelerated evolution among all annotations (Fig. 2d), exceeding both intergenic and intronic regions in every species–tissue combination (Table S6) and surpassing rates expected under a neutral model (Figs. S5 and S6; Tables S6 and S7; see “Supplementary information”), thereby underscoring the rapid divergence of CREs at gene promoter sites. Among 5,536 orthologous genes (202 to 4,321 genes across tissues) with positionally “overlapping” promoter peaks across all species (Anc1, Fig. 1d), over half (3,061 orthologous genes, 55%) demonstrated accelerated evolution (see “*Methods*”), with significant enrichment for genes tied to key developmental processes such as retinal development and cell junction assembly (Fig. S7). This prevalent pattern reveals that even deeply conserved gene promoter peaks can undergo substantial sequence divergence in at least one lineage, facilitating clade-specific functional adaptations. In the five cichlids, these accelerated promoters represent candidate loci where discrete nucleotide changes could plausibly affect gene regulation and thus, the evolution of key adaptive traits, though this requires functional validation.

To further quantify regulatory evolution, we measured gain and loss of promoter peaks across each lineage in relation to the corresponding genetic diversity (Fig. 2e, Tables S8 and S9). With no previous knowledge of expected divergence at open chromatin sites, we use overlapping *O. niloticus* peak summits as a reference in all comparisons (Table S8, see *Methods*). Pairwise comparisons based on overlapping promoter peaks of 1,956 to 4,983 1-to-1 orthologous genes revealed that 37% to 66% (mean of 52%) of peaks across all species and tissues retained strong sequence conservation (less than 10% divergence, Table S8), consistent with 85% to 89% pairwise nucleotide identity across cichlid genomes (Mehta et al. 2022) (see *Methods*). However, species- and tissue-specific differences in gene promoter peak gains were also evident and unbiased by genome completeness or annotation quality (see “*Supplementary information*”); for example, *N. brichardi* exhibited the most gains in retina (52% of all 13,889 1-to-1 orthologous genes), while *M. zebra* showed the highest gains in forebrain tissue (45% of 1-to-1 orthologous genes)—often enriched (Benjamini–Hochberg [BH] (Benjamini and Hochberg 1995) adjusted *P-value* <0.05) for relevant tissue-specific functions, such as neurogenesis (in *M. zebra)* or monatomic ion transport (in *A. burtoni*; Fig. S8). Peak loss was similarly variable across species, with the majority (76% to 91%) of losses occurring in regions with elevated sequence divergence (greater than 10% difference; Fig. 2e, Table S8), suggesting that sequence evolution frequently underpins regulatory site turnover, including the potential gain or loss of TFBSs.

### TF Binding Landscapes are Extensively Reshaped at Active Regulatory Sites

TFs are central drivers of gene regulatory innovations, shaping networks associated with cichlid phenotypic diversity (Mehta et al. 2021, 2022, 2023). To systematically profile TF-mediated regulation, we took advantage of our experimental ATAC-Seq data to map between 54,000 and 226,000 TF footprints within gene promoters (representing 8% to 13% of each genome) across four tissues and five species, using 1,442 unique TF motifs spanning cichlid (Mehta et al. 2021) and vertebrate (Kulakovskiy et al. 2013; Rauluseviciute et al. 2024) lineages. Profiling of footprints with 1,442 vertebrate motifs yielded between 530,000 and 2,200,000 nonredundant TFBSs (Table S10a and b, see *Methods*), with a mean of 1.4 million nonredundant promoter TFBSs per sample; this is remarkably consistent with the 3.6 million footprints mapped to the ∼3 × larger human genome (Vierstra et al. 2020).

Consistent with previous work (Mehta et al. 2021, 2022, 2023), we found that TFBSs are significantly enriched (BH (Benjamini and Hochberg 1995) adjusted *P-*value <0.05) in noncoding regulatory regions, especially gene promoters and conserved noncoding elements (CNEs; Fig. 3a and Table S11). Notably, TFBSs are over four-fold more abundant in highly conserved CNEs (hCNEs with sequence identity ≥90% over ≥30 bp, see *Methods*), identifying TF binding sites maintained by selective constraint. In some tissues and species, e.g. *O. niloticus* forebrain and *N. brichardi* retina (Fig. 3a and Table S11), TFBSs are more than two-fold enriched in accelerated CNEs (aCNEs with sequence identity <90% over ≥30 bp and significantly deviating from a neutral model, see *Methods*)—highlighting a set of novel, potentially lineage-specific regulatory sites, such as the previously characterized NR2C2-*sws1* interaction in *N. brichardi* (Mehta et al. 2021).

**Fig. 3.**
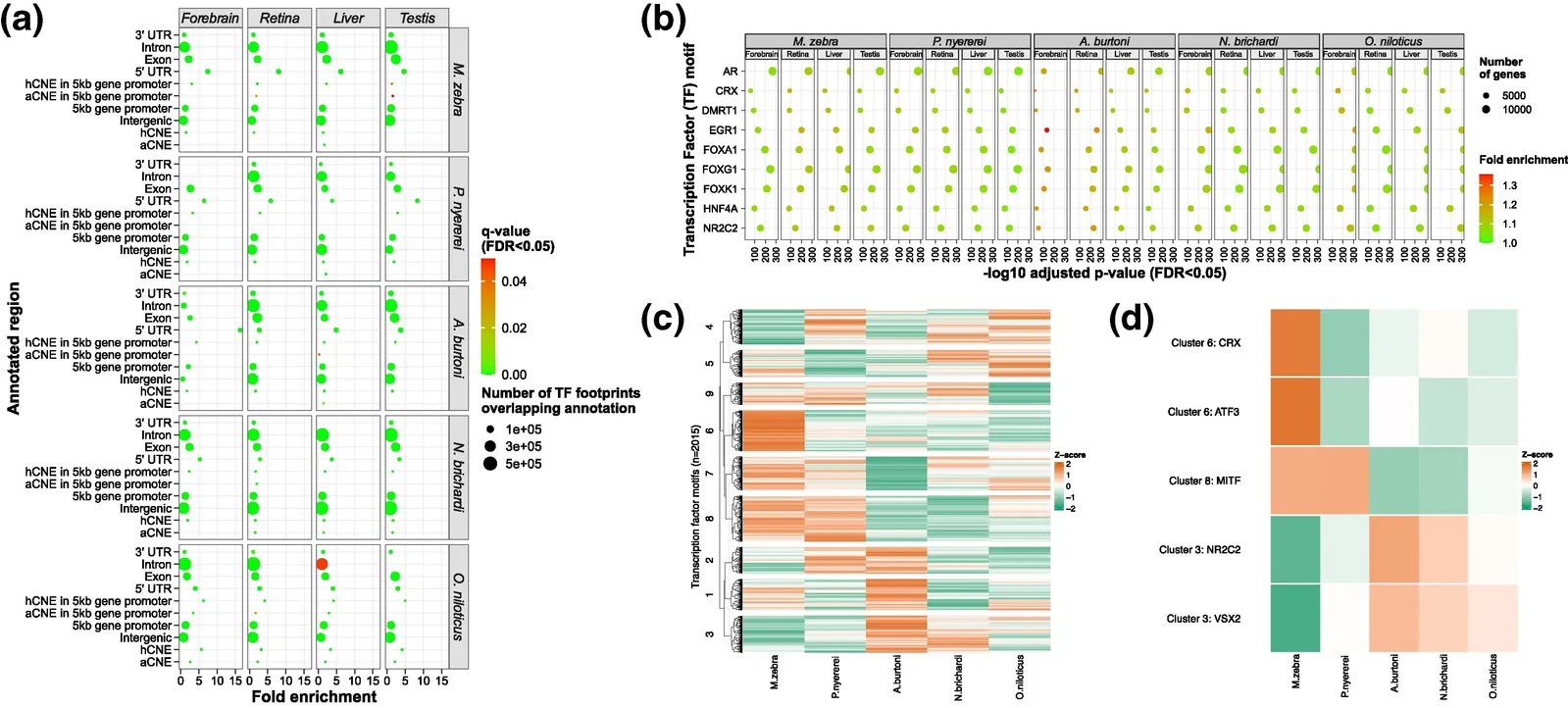
Transcription factor motif profiling of chromatin accessibility evolution in five cichlids. (A) Fold enrichment of TF motifs overlapping coding and noncoding regions across species and tissues. Circles show enriched annotated region (y-axis) of significance (FDR <0.05, to right) and fold enrichment (x-axis) values of all annotated peaks. Number of TF motifs overlapping each annotation shown by size of each circle. (B) Enrichment of nine functionally associated TF motifs in gene promoter peaks across species and tissues. Circles show TF motif (y-axis) with fold enrichment (heatmap to right) and significance of enrichment as -log10 FDR <0.05 (x-axis) across each species and tissue. Number of enriched genes for each TF motif shown by size of each circle. (C) Clustering of TF activities in gene promoter regions of retina tissue across the five species. Each row represents a single, clustered TF, and each column a species. Mean TF bit-score of motif match across gene promoters (as a measure of activity) from HINT-ATAC are Z-score transformed across rows. Green colour indicates low activity and orange colour indicates high activity. (D) TF activities and cluster assignment of five eye-related TFs in gene Li et al. 2019 promoter regions of retina tissue across the five species. Each row is an eye-related TF, and each column a species. Mean TF bit-score of motif match across gene promoters (as a measure of activity) from HINT-ATAC Li et al. 2019 are Z-score transformed for each TF. Green colour indicates low activity and orange colour indicates high activity.

To explore TF functional relevance, we identified 824 (57%) of the 1,442 input vertebrate TF motifs significantly enriched (BH (Benjamini and Hochberg 1995) adjusted *P*-value <0.05) in active promoter regions across tissues (Table S12), many with known roles in vertebrates, as well as cichlid adaptive traits. Topmost enriched examples across species include *foxg1* and *egr1* in the forebrain (implicated in neural diversity (Sylvester et al. 2013) and behavior (Burmeister et al. 2005)), *crx* and *nr2c2* in the retina (opsin gene regulation (O'Quin et al. 2011; Mehta et al. 2021)), *hnf4a* and *foxa1* in the liver (diet (Vernaz et al. 2021) and hepatogenesis (Lee et al. 2005)), and *ar* and *dmrt1* in the testis (sex differentiation (Wei et al. 2019) and reproductive traits (Hoadley et al. 2022); Fig. 3b, Table S12). To assess whether these TFs are expressed in the corresponding tissue, we cross-referenced RNA-seq TPM data for these examples (see “Methods”): *foxg1, egr1, nr2c2, hnf4a, ar,* and *dmrt1* showed clear expression (mean TPM approximately 0.15 to 42) in their respective tissue across all five species, and *crx* was expressed in four of five species' retina. These examples suggest that TFs with deeply conserved roles in vertebrate development and physiology can function through highly dynamic binding landscapes, linking divergence in motif usage and TF activity to the potential diversification of complex traits within a single radiation.

Clustering tissue-specific TF activity within each species (see *Methods*) revealed distinct coactive groups of TFs (Fig. 3c, Figs. S9 to S11), reflecting both conserved and divergent patterns of transcriptional regulation (see “Supplementary information”). In retina tissue (Fig. 3c), clear differences emerged among species: for example, *M. zebra* exhibited strong co-activity (*Z*-score of 1.6 vs. −1 to 0.05 in other species, Fig. 3d) of *crx* and *atf3*, which are key regulators of retinal development (Kole et al. 2020; Mehta et al. 2022) and opsin expression (Takechi et al. 2008) (Fig. S12), while *P. nyererei* and *M. zebra* shared high *mitf* activity (Z-score of 1 vs. −0.1 to −1 in other species, Fig. 3d), related to retinal pigment epithelium formation (Lane and Lister 2012) (Fig. S12). Conversely, *A. burtoni* and *N. brichardi* showed higher activity for *nr2c2* and *vsx2* (*Z*-score of 1.1 and 0.6 vs. 0 to −1.5 in the other species; Fig. 3d; Fig. S12), implicating divergent regulatory programs for these genes associated with cichlid visual system adaptation (Mehta et al. 2021).

Taken together, these results demonstrate that divergence in TF binding activity—through both conserved and lineage-specific regulatory motifs—could enable regulatory plasticity of genes underlying cichlid adaptive traits, reinforcing associations between regulatory innovation and cichlid phenotypic diversification.

### Dynamic Regulatory Site Divergence is Associated With Regulatory Rewiring in Cichlids

To directly link the observed divergence of TF binding activity with genetic changes in regulatory elements, we examined nucleotide variation at active TFBSs in orthologous gene promoters. Using the phylogenetic outgroup species, *O. niloticus*, as the reference (see *Methods*), we found that the vast majority (76% to 78%) of TF motifs in the other four cichlid species only partially positionally overlap (<100%) with the reference, and nearly all motifs (99.1% to 99.9%) differed in at least one position or context, including single-nucleotide substitutions or positional shifts, which do not necessarily alter TF-binding capacity (Fig. 4a, Table S13). This points to pervasive divergence of TF binding landscapes, even at orthologous promoter regions across species, offering a potential mechanism by which closely related lineages can rapidly evolve distinct adaptive phenotypes.

**Fig. 4.**
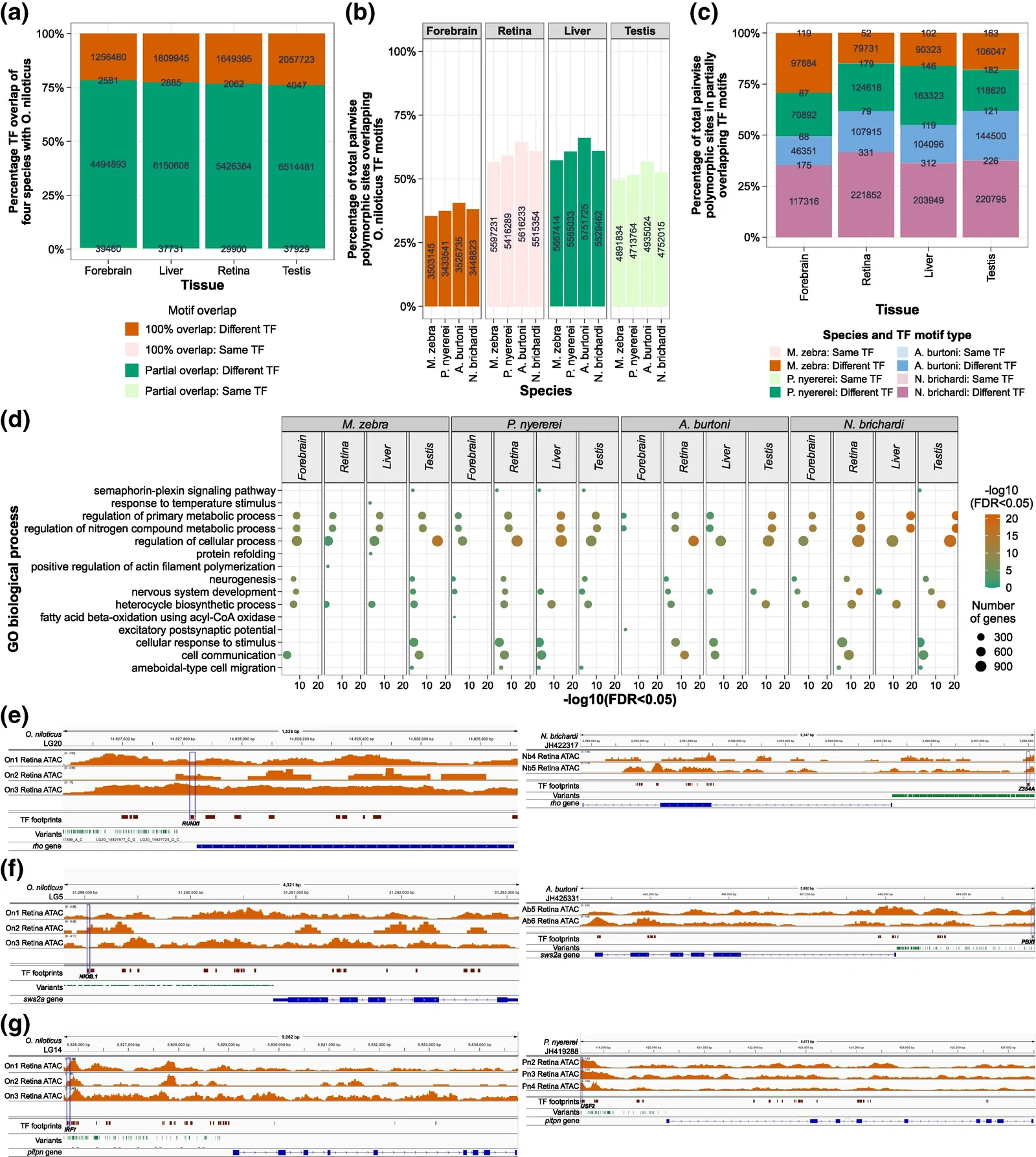
Nucleotide divergence of actively bound TFs across five cichlids. (A) Percentage TF motif overlap of same or different motifs of four species versus *O. niloticus* reference across four tissues. Number of TF motifs within each category are shown within each bar. (B) Percentage of total pairwise polymorphic sites overlapping *O. niloticus* TF motifs in the four species and tissues. Number of pairwise polymorphic sites in each species tissue versus *O. niloticus* shown within each bar. (C) Percentage of total pairwise polymorphic sites in partially overlapping same or different *O. niloticus* TF motifs in the four species and tissues. Number of pairwise polymorphic sites overlapping same or different TF motifs in each species tissue versus *O. niloticus* shown within each bar. (D) Gene ontology (GO) biological process enrichment of accessible gene promoter regions containing pairwise polymorphic variants versus *O. niloticus* across the four species and tissues. GO terms are a subset of enrichment -log10 (FDR<0.05) of >1.3. Circles show enriched term (y-axis) with significance of enrichment as -log10 FDR <0.05 (x-axis and heatmap to right) across each species and tissue. Number of enriched genes for each GO term shown by size of each circle. Species chromosome tracks of (E) *O. niloticus* (left) and *N. brichardi* (right) *rho* gene; (F) *O. niloticus* (left) and *A. burtoni* (right) *sws2a* gene; and (G) *O. niloticus* (left) and *P. nyererei* (right) *pitpn* gene. Coding sequence shown as blue annotations including upstream gene promoter regions, accessible peaks across retina tissue biological replicates (orange peaks), TF footprints (maroon mark), and pairwise polymorphic variants (green marks). Candidate actively bound TF footprints with pairwise variation are labelled highlighted with blue box.

To quantify the genetic basis of these regulatory differences, we identified 9 to 10 million pairwise polymorphic sites within aligned promoter regions of 10,699 out of the 13,889 1-to-1 orthologous genes across the five species (Table S14a). On average, 53% of promoter variants (range 35% to 66%) fell within and overlapped predicted *O. niloticus* TF binding motifs in promoters of 6,842 to 8,200 orthologous genes per tissue (Fig. 4b, Table S14b). These motifs carried only 43% as many variants per affected gene promoter versus those promoters without variants overlapping motifs (paired *t*-test across 16 species-tissues: *t* = −16.8, *P* = 3.9 × 10^−11^), indicating purifying selection. We defined a “principal set” of 1% to 5% of all promoter variants (1,385 to 1,439 of the 1,442 nonredundant input vertebrate TF motifs in 749 to 2,747 genes) as those disrupting partially (<100%) overlapping motifs between species and *O. niloticus,* where 99.9% of motifs diverged to a different TF identity, possibly arising from substitutions and/or short indels within the position weight matrix (PWM) footprint that requires further testing (Table S14c). Notably, 6% to 13% of principal set variants (175 to 1,361 motifs in up to 618 orthologous genes) occurred in accelerated peaks (Table S14c). These regions likely represent hotspots of regulatory innovation driving species-specific regulatory changes.

Functional annotation of “principal set” genes affected by TFBS divergence revealed significant enrichment (BH (Benjamini and Hochberg 1995) adjusted *P*-value <0.05) for biological processes central to vertebrate traits, such as neurogenesis in the forebrain and cellular process regulation in the retina (Fig. 4d). This includes known cichlid adaptive trait genes like, for example, in the *bmpr1* and *rho* promoters of *P. nyererei* and *N. brichardi,* a total of four nucleotide changes (vs. *O. niloticus*) alter motifs from CTCFL/RUNX1 to ZN768/Z354A, implicating regulatory evolution in their functions of jaw morphology (Terai et al. 2002) and dim-light vision (Sugawara et al. 2002; Ricci et al. 2022) (Fig. 4e). Similarly, in *M. zebra* and *A. burtoni*, five and six respective variants in the *cntn4* and *sws2a* promoters result in turnover from ZNF263/NKX6.1 to MEIS1/PBX1 motifs, potentially affecting how these two genes that are linked to either morphogenesis (Brawand et al. 2014) and visual system (Carleton 2009) divergence (Mehta et al. 2021, 2022) are regulated (Fig. 4f). Both discrete (single-nucleotide change) and complex motif rewiring (multiple nucleotide changes)—such as the replacement of IRF7 with USF2 motifs in the promoter of the retinal function related gene, *pitpn* (Ile et al. 2010) (Fig. 4g) and STAT1 with a TEAD4 motif in the promoter of the liver development gene, *foxa2* (Lee et al. 2005)—were also observed in additional tissue- and lineage-specific contexts (see “*Supplementary information*”).

Together, these patterns of nucleotide divergence at candidate regulatory binding sites are consistent with a role in GRN rewiring, representing testable hypotheses for how regulatory sequence change could contribute to the evolution of adaptive traits across lineages.

### Regulatory Divergence in Chromatin Accessibility Associates With Transcriptional Changes in Tissue-specific and Adaptive Genes

While transcriptional variation can underlie cichlid diversification (El Taher et al. 2021), the precise role of epigenetic regulation in driving tissue-specific transcriptomic divergence across East African lineages remains understudied. To address this, we integrated chromatin accessibility and RNA-sequencing data from matched tissues across the five cichlid species (see “*Methods*”, Fig. 1a, Table S1). Among the 23,044 to 23,568 protein-coding genes per genome, we quantified expression (as transcripts per million, TPM) for 13,121 to 21,163 genes (56% to 75% of all coding genes per species). Within each species-tissue dataset, 20,652 to 226,889 accessible chromatin peaks (47% to 94% of identified peaks) were associated with the expression of 6,657 to 18,787 genes (29% to 70% of protein-coding genes per genome; Table S15). These differences reflect tissue- and species-specific regulatory complexity, e.g. testis and forebrain have the highest numbers of associated peaks (129,254 to 166,802) and genes (13,374 to 15,991) on average, whereas liver (90,452 peaks and 10,331 genes) and retina (83,902 peaks and 12,122 genes) show fewer (Table S15), rather than absence of gene expression.

Assessing regulatory relationships, we confirmed a weak but significant positive correlation between promoter chromatin accessibility (within 5 kb of the TSS) and gene expression level in 18 out of 20 species-tissue datasets (Spearman's *R* = 0.014 to 0.21, *P* < 0.05; Figs. S13 to S17). Although statistically significant, these correlation coefficients are low, indicating that promoter chromatin accessibility alone explains only a small proportion of the variance in gene expression level, consistent with transcription being jointly determined by additional layers of regulation not captured here (e.g. distal enhancers, TF activity, post-transcriptional control). Exceptions included *A. burtoni* (*R* = −0.02, *P* < 2e−7) and *M. zebra* (*R* = −0.003, *P* = 0.53) liver, where the correlation was weak or absent (Figs. S13 and S15). Overall, the weak but generally positive correlation is consistent with findings from other *O. niloticus* tissues (Mehta et al. 2023) and from mammalian systems, where accessible promoters can mark active, repressed, or poised states (Shlyueva et al. 2014). In the five cichlids, the same multifaceted relationship between accessibility and transcription appears to have been utilized during diversification, allowing similar regulatory architectures to support both conserved and lineage-specific expression programs.

To dissect regulatory modes, we categorized genes based on peak accessibility—high (HA) or medium/low (MA)—and by expression level—high (HE) or medium/low (ME; see “*Methods*”). Of all identified nonredundant peaks, 5,222 to 68,245 (12% to 28%) mapped to 1,864 to 5,404 genes (8% to 20% of coding genes per genome; Figs. S18 to S22, Table S16). In retinal tissue, where adaptive regulatory divergence is marked (Fig. 4), most peak–gene relationships (37% to 41%) fell into the HA–HE category (1,408 to 4,842 peaks and 902 to 2,020 genes per species; Figs. S18 to S22, Table S16). The remainder were distributed as HA–ME (23% to 24%), MA–HE (20% to 24%), and MA–ME (15% to 16%; Fig. 5a, Figs. S18 to S22, Table S16).

**Fig. 5.**
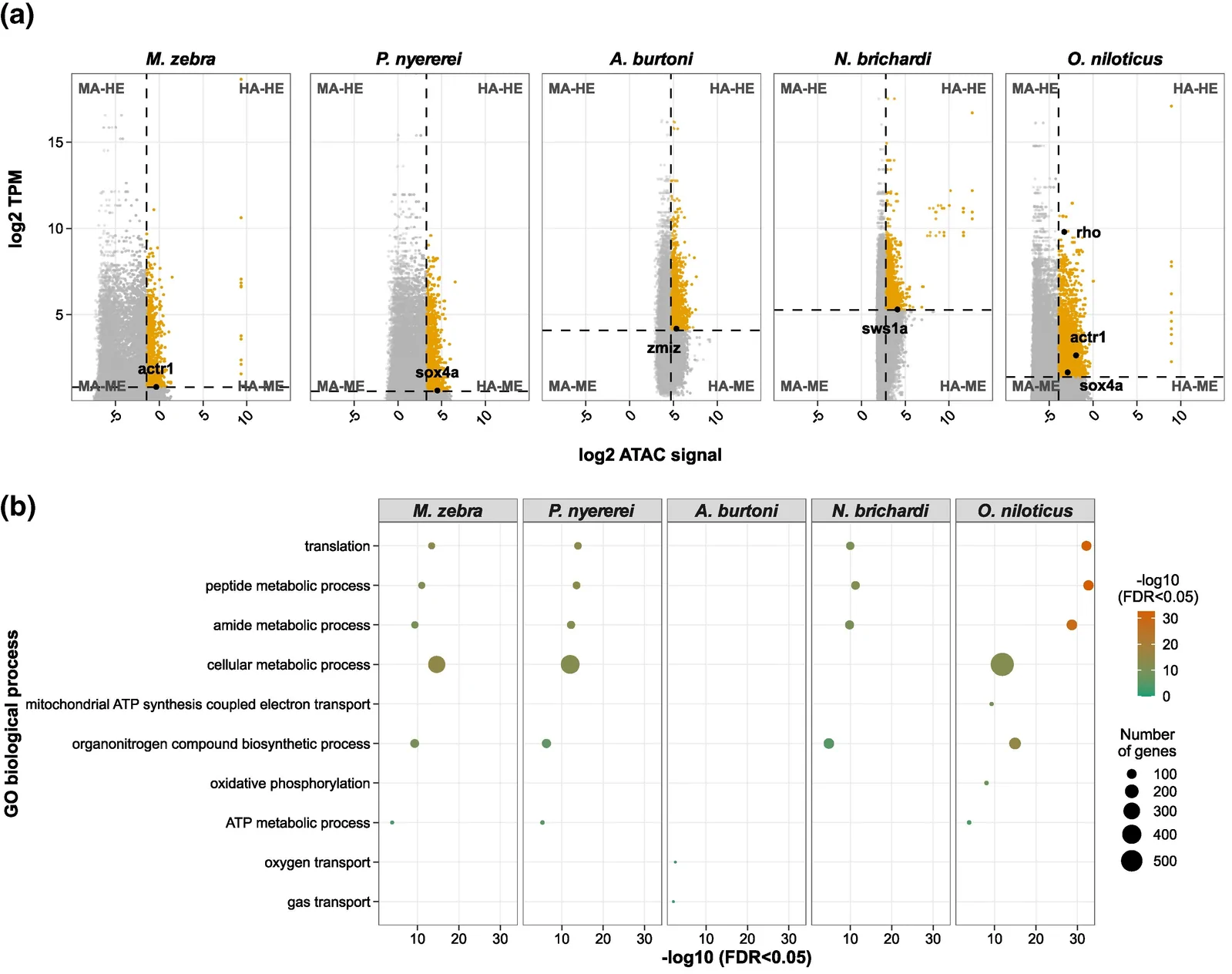
Accessible promoter peaks accounting for gene expression changes in retina tissue across five cichlid species. (A) Scatterplots of average log2 gene expression (as transcripts per million - TPM, y-axis) versus average log2 ATAC signal (up to 5 kb gene promoter peak counts, x-axis) for 1-to-1 orthologous genes. Genes are categorised by combined accessibility and expression levels into four groups: high accessibility and high expression (HA-HE; both above the 70th percentile, and shown with orange dots), high accessibility and medium-low expression (HA-ME; accessibility above 70th percentile and expression below 50th percentile), medium-low accessibility and high expression (MA-HE; accessibility below 50th percentile and expression above 70th), and medium-low accessibility and medium-low expression (MA-ME; both below 50th percentile). Representative candidate genes are labelled within each plot. (B) Gene ontology (GO) biological process enrichment for HA-HE genes across species. Ten selected enriched GO terms (–log10 FDR > 2.1; full set in Supplementary Fig. S23), with circles sized by number of enriched genes and positioned according to GO term (y-axis) and enrichment significance (–log10 FDR<0.05, x-axis and heatmap).

Examining the HA–HE group in retina tissue revealed significant positive correlations in *M. zebra* (*R* = 0.1, *P* < 2.2e−16), *N. brichardi* (*R* = 0.08, *P* = 6.9e−08), and *O. niloticus* (*R* = 0.2, *P* < 2.2e−16), while *P. nyererei* (*R* = −0.03, *P* = 8.5e−04) showed a weak negative correlation, and in *A. burtoni*, no correlation (Figs. S18 to S22), paralleling trends in other tissue regulatory categories (Figs. S19 and S20). Such patterns, as also found in *O. niloticus* gill (Mehta et al. 2023), indicate that HA−HE genes are consistent with activated, repressed, or poised states in retinal tissue across the five species; however, this would need to be validated with relevant histone mark data. Functional enrichment (BH (Benjamini and Hochberg 1995) adjusted *P* < 0.05) among these 902 to 2,020 HA–HE retina genes identified both shared (e.g. cellular metabolic process in *M. zebra, P. nyererei,* and *O. niloticus*) and species-specific (e.g. oxygen transport in *A. burtoni*, mitochondrial ATP synthesis in *O. niloticus*) GO terms (Fig. 5c, Fig. S23), highlighting critical processes for retinal function and visual evolution in fishes (Damsgaard et al. 2020). The HA–HE regulatory group captured key vision-associated genes (Fig. 5a), such as the retinal regulator *actr1* (Whited et al. 2004) (*M. zebra, O. niloticus*), eye development gene *sox4* (Wen et al. 2015) (*P. nyererei, O. niloticus*), eye morphogenesis gene *zmiz* (Foerst-Potts and Sadler 1997) (*A. burtoni*), the dim-light vision gene *rho* (*O. niloticus*) with regulatory TFBS divergence (Mehta et al. 2022) (Fig. 4e), and the visual opsin gene *sws1* (*N. brichardi*), linked to ecological GRN rewiring in cichlids (Mehta et al. 2021).

In summary, conserved and lineage-specific patterns of promoter accessibility are closely associated with the transcriptional activity of genes linked to adaptive traits, especially in the visual system. These results support a general view in which changes in chromatin state at key regulatory regions provide an important axis along which gene expression and complex phenotypes can evolve within a vertebrate radiation (Lowe et al. 2011; Ogawa et al. 2025).

### Motif-supported Regulatory Networks Reveal Pervasive, Tissue-dependent Rewiring Across Cichlid Species

As previous studies have demonstrated that rapid evolution of regulatory elements (Brawand et al. 2014) and GRN rewiring (Mehta et al. 2022) may contribute to cichlid diversification, we built on this—integrating the matched chromatin accessibility and transcriptional data—to infer tissue- and species-specific GRNs that could dissect regulatory relationships underlying adaptive divergence across the five cichlid species (see “*Methods*”). GRNs were first reconstructed using GENIE3 (Huynh-Thu et al. 2010), a random forest-based method that frames GRN inference as a set of regression tasks to predict each gene's expression from all others, yielding ranked TF–target gene edges by variable importance, without requiring prior knowledge of interactions. We selected GENIE3 for its strong performance on benchmark datasets (Huynh-Thu et al. 2010) and then incorporated motif footprints from accessible promoter regions as an orthogonal filter for regulatory support.

Using expression data alone, large tissue-specific networks were reconstructed (ranging from ∼117 k to 11.6 M edges per species-tissue combination; Table S17a), but the top 12,000 edges per network—the minimum number allowing fair comparison across all datasets without exhausting smaller “filtered” networks below—yielded mostly species-specific structure (Fig. S24a). GRN rewiring analysis (see “*Methods*”) identified that, of the nonredundant top edges, 97.6% to 99.7% (47,708 to 59,373 edges) were species-specific across tissues, 0.3% to 2.4% (146 to 1,380 edges) shared in subsets of species, and none conserved across all five species (Fig. 6a). To test whether this reflects genuine specificity rather than an artefact of the top-12,000 cutoff, we performed a sensitivity analysis: across a 4,000 to 50,000 edge threshold range, the species-specific fraction remained high (93.4% to 99.9%), and for edges classified as species-specific at 12,000, only 0.3% to 2.5% ranked just outside the cutoff (12,001 to 20,000) in another species' full network, with the majority either absent from or ranked beyond the top 50,000 edges of every other species' network (Fig. S24b; Table S17c and d; see “*Methods*”). Based on gene expression edges only, Jaccard similarity matrices confirmed low GRN top-edge overlap between species (all <0.03; Fig. S25), while multidimensional scaling (MDS) ordinations revealed tissue-dependent separation in GRN space, with mostly different species clustering across tissues (forebrain–*M. zebra/A. burtoni/N. brichardi*, retina–*M. zebra/P. nyererei*, liver–*M. zebra/A. burtoni*, testis– *M. zebra/A. burtoni*; Fig. S26).

**Fig. 6.**
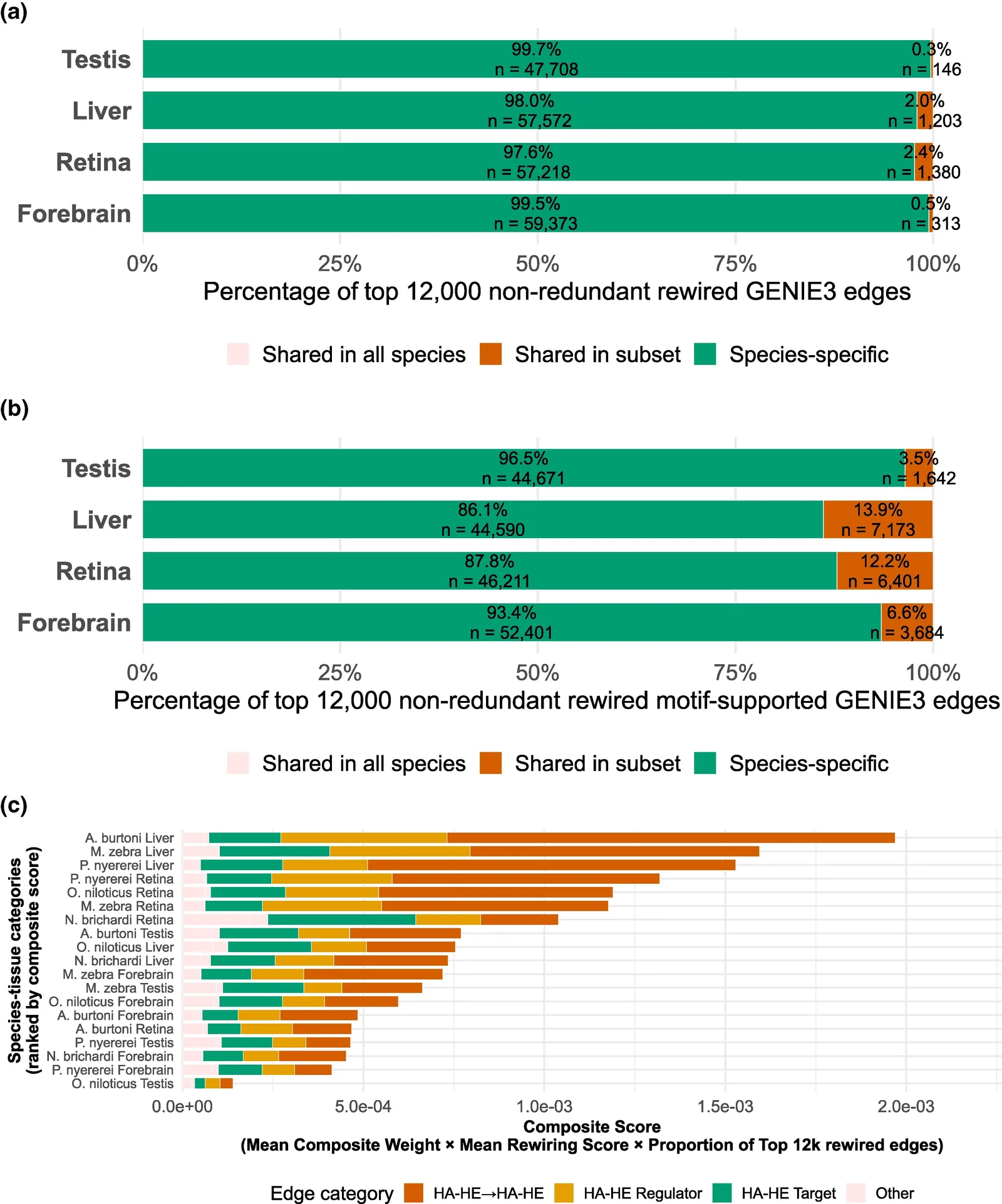
Species-specific and HA-HE-associated GRN rewiring across tissues and ranked composite scores in the five cichlids. (A) Distribution of the top 12,000 non-redundant GENIE3 rewired edges across Forebrain, Retina, Liver and Testis, showing the proportion of species-specific edges (green) and the smaller fractions shared in subsets of species (orange) or conserved across all species (pink). (B) Distribution of the top 12,000 non-redundant motif-supported GENIE3 rewired edges across the same four tissues, highlighting the same enrichment for species-specific rewiring (green) and the limited share of edges shared in subsets (orange) or across all species (pink). (C) Ranked composite score plot of species-tissue categories (y-axis) based on mean composite weight × mean rewiring score × proportion of top 12k rewired edges (x-axis), showing that the highest-ranked categories are dominated by HA-HE-associated edges. The stacked bars distinguish HA-HE → HA-HE (orange), HA-HE regulator (yellow), HA-HE target (green), and other (pink) categories, and the ranking is shown for all 96 species-tissue categories.

Refining networks by motif footprints—normalizing maximum orthologous motif bit scores (BSs) and computing composite weights (GENIE3 importance × normalized motif BS, see “*Methods*”)—identified ∼14k to 1.5 M edges across species tissues, yielding sparser (7 to 11-fold reduction), motif-supported GRNs compared to expression-only networks (Table S17b). Jaccard similarity (all <0.13; Fig. S27) and MDS ordinations identified clearer phylogenetic patterns of conserved regulatory edges (e.g. liver–*M. zebra/P. nyererei/A. burtoni*) and tissue-specific divergence (e.g. *P. nyererei* and *O. niloticus* are isolated in forebrain and testis GRN space, while *A. burtoni, N. brichardi* and *O. niloticus* are separated in retina; Fig. S28). GRN rewiring analysis showed a 5- to 12-fold increase (compared to expression only GRNs) in shared-subset (1,642 to 7,173) edges across tissues; however, most nonredundant top edges, 86.1% to 96.5% (46,314 to 56,085 edges), are species-specific (Fig. 6b); this is supported by ranking rewired edges by species-specificity, weight delta, and rank advantage relative to orthologs (see “*Methods*”; Figs. S29 to S32).

To identify candidate edges associated with regulatory divergence, we classified GRN orthologs by chromatin accessibility (high [HA]/medium-low [MA]) and expression (high [HE]/medium-low [ME]) status in each species-tissue context, then tested for enrichment among all rewired edges (see “*Methods*”). Edges where both regulator and target exhibit high accessibility and expression (HA–HE → HA–HE) were lowly enriched in only two samples (1.1-fold in *A. burtoni* forebrain, BH (Benjamini and Hochberg 1995) adjusted *P* = 1.4 × 10^−26^; 1.4-fold in *O. niloticus* testis, BH (Benjamini and Hochberg 1995) adjusted *P* = 8.4 × 10^−90^), and MA–ME-only edges in only five samples (1 to 1.3-fold, BH (Benjamini and Hochberg 1995) adjusted *P* < 0.01) (Table S18; Fig. S33). Meanwhile, HA–HE regulator and HA–HE Target edges also showed no to low enrichment in 11 (1- to 1.4-fold, BH (Benjamini and Hochberg 1995) adjusted *P* < 10^−4^) and 12 (1- to 1.3-fold, BH (Benjamini and Hochberg 1995) adjusted *P* < 0.04) samples, respectively, with the highest mean enrichment in HA–HE target edges of retina tissue (1.2-fold across four species, BH (Benjamini and Hochberg 1995) adjusted *P* < 5.4 × 10^−8^) (Table S18; Fig. S33). However, all HA–HE associated edges (as either or both regulator and target) consistently occupy the upper tails of composite weights and rewiring scores across all rewired edges (Figs. S34 to S37), and when ranking based on a composite of these scores and proportion of top 12,000 species-tissue rewired edges occupied by category, the top 30 (out of 96 species-tissue categories) are all HA–HE associated edges (Fig. 6c; Table S19). The overall top 10 species-tissue categories are HA–HE associated edges in liver (*A. burtoni, P. nyererei, M. zebra*), retina (*P. nyererei*, *O. niloticus*, *M. zebra*, and *N. brichardi*), and forebrain (*M. zebra*) (Fig. 6c; Table S19), highlighting the importance of dual high accessibility/expression signals in motif-supported GRNs and their potential rewiring for functions of these tissues.

All rewired HA–HE → HA–HE edges were then prioritized to identify the top 50 candidates by composite weight × rewiring score, species-specificity (all unique to one species-tissue) and strong motif support (mean motif bitscore = 14.4, range 11 to 23). Within this set, dominated by only retina (27 out of 50) and liver (23 out of 50) edges, we identified candidate rewired edges that could be associated with regulatory divergence of tissue-specific traits of the five species, including *O. niloticus* retina CEBPA-*mgme1* (composite weight = 0.052, rewiring score = 13.8) which could be linked to retinal morphology (Milenkovic et al. 2022) and *A. burtoni* liver HNF1A*-foxk1* (composite weight = 0.068, rewiring score = 8.7) that could be linked to liver metabolic processes (Gong et al. 2004) (Table S20).

Overall, motif-supported GRNs exhibit rapid regulatory evolution, with 86% to 97% of top edges being species-specific and thus, rewired along the phylogeny, yet tissue-dependent phylogenetic signal is retained in shared edges. High accessibility and expression (HA–HE) edges—integrating chromatin accessibility, transcriptional activity, and motif support—are consistently rewired across tissues, identifying a focused set of candidate edges that could be linked to tissue function across the species. Building on the prior observation that chromatin accessibility patterns predict transcriptional divergence in adaptive trait genes, these GRN results prioritize regulatory relationships that could contribute to ecological adaptations at the network level.

### Functional Variation at Active TF Binding Sites Tracks Phylogeny and Ecology Within the Cichlid Radiation

To interrogate functional regulatory divergence, we genotyped TFBS variants of HA–HE and GRN rewired genes across the five cichlid species in deep phylogenies of related riverine and lacustrine species. Our aim was to identify orthologous gene promoter variants that reflect niche- or clade-specific adaptation. To prioritize 1-to-1 orthologous genes, we ranked TFBSs in promoter regions using high tag counts (TC, number of reads) and BSs, selecting statistically significant sites above the species-tissue mean (BH (Benjamini and Hochberg 1995) adjusted *P* < 0.0001; see “*Methods*”).

In retina tissue, we identified 896,634 to 2,247,267 unique TFBSs in 93,139 to 226,058 promoter footprints, spanning 13,774 to 21,553 genes across the five species. Of these, 108,193 to 354,906 (10% to 20%) TFBSs in 18,726 to 59,883 (15% to 32%) footprints within 6,844 to 13,886 (41% to 76%) genes exhibited above-mean TC and BS (Fig. 7a, Table S21), comparable to findings in *O. niloticus* gill (Mehta et al. 2023). Within this candidate set, we focused on TF–target gene (TF–TG) relationships relevant to vision and ecological adaptation (Carleton 2009), especially where TFBS variants correlated with transcriptional divergence, e.g. *O. niloticus* RUNX1–*rho*, *P. nyererei* USF2–*pitpn*, and *N. brichardi* NR2F6–*sws1* (Fig. 7a).

**Fig. 7.**
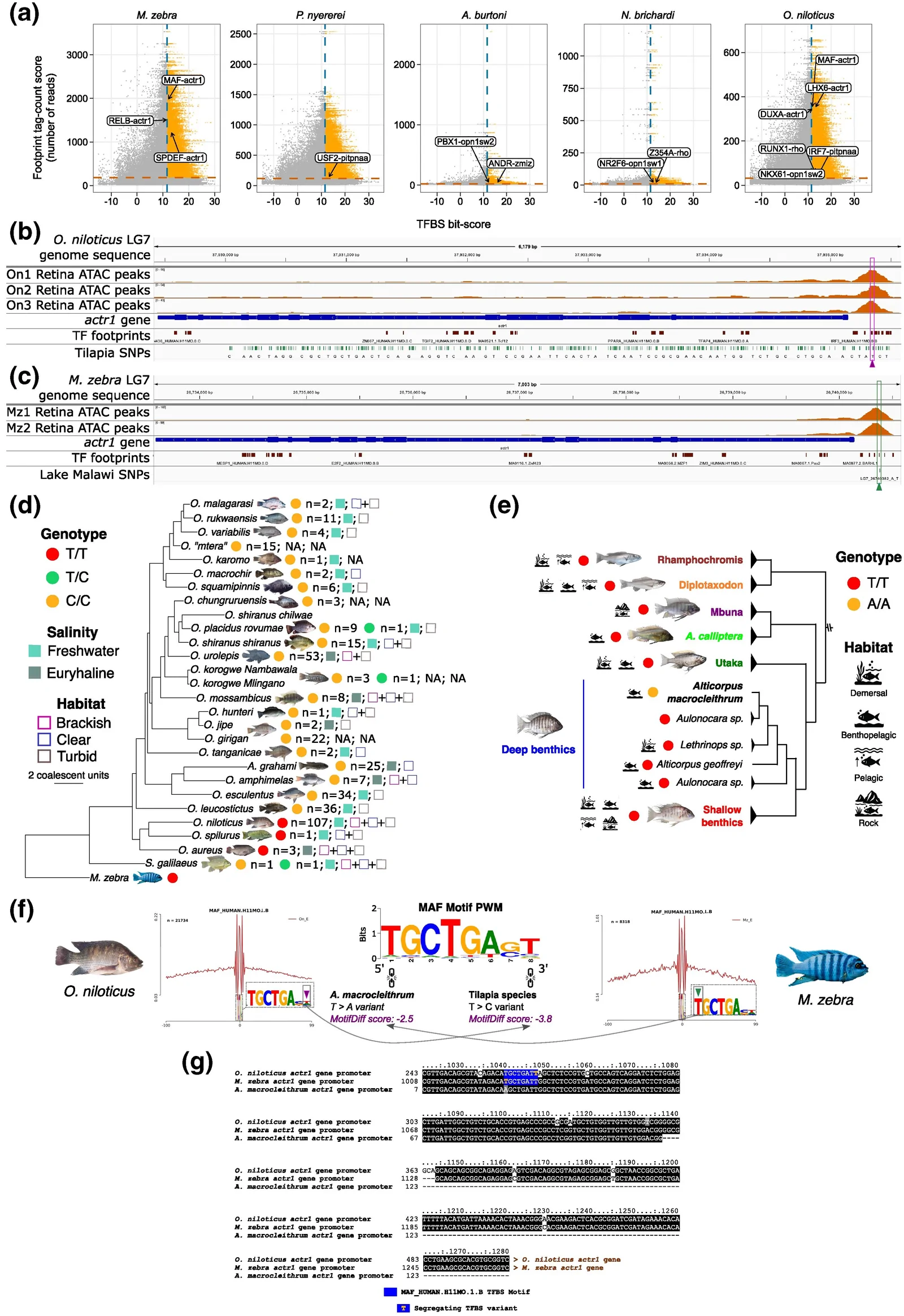
Chromatin accessibility and MAF transcription factor motif variation in the actr1 promoter of *O. niloticus* and *M. zebra*. (A) Scatterplot of tag-count (TC) scores of transcription factor (TF) footprints (y-axis) versus motif bit-scores of predicted TF binding sites (x-axis) detected in accessible retina chromatin. TFBSs exceeding the mean TC (orange dotted line) and mean bit-score (blue dotted line) are shown in orange, while those below these thresholds are shown in grey. Representative TF–target gene candidates are highlighted with arrows. (B) Genome track of *O. niloticus actr1* on LG7 with gene annotations (blue), accessible chromatin peaks across three retina replicates (orange), TF footprints (maroon), tilapia SNPs (green), and a polymorphic MAF motif site (pink box/arrow). (C) Corresponding *M. zebra* track showing two retina replicates (orange), TF footprints (maroon), Lake Malawi cichlid SNPs (green), and polymorphic MAF motif site (green box/arrow). (D) SNP genotypes at the site in (B) mapped onto the tilapia phylogeny Ciezarek et al. 2024  Mehta et al. 2023b: homozygous reference (red), alternate (green), and heterozygous (yellow). Species’ habitats and salinity adaptations annotated. (E) SNP genotypes at the site in (C) across the Lake Malawi phylogeny Malinsky et al. 2018  Blumer et al. 2025: homozygous reference (red) and alternate (yellow), with habitats indicated. Expanded genotype-phenotype-ecotype phylogeny in Supplementary Fig. S38. (F) MAF footprint profiles in *O. niloticus* (left) and *M. zebr*a (right), showing signal intensity (red line) centered on footprint (position 0, x-axis). Sequence variation from panels A (purple arrow) and B (green arrow) indicated, with MotifDiff score of these variants indicated on input position weight matrices (PWM) of MAF motif used for prediction shown centrally. (G) Predicted MAF motifs (blue shading) and polymorphic sites (orange text) in *O. niloticus* and *M. zebra actr1* gene promoter. Orthologous alignment with *A. macrocleithrum* shown below with corresponding *M. zebra* MAF site substitution shown in light grey shading. Additional alignments across other Lake Malawi cichlid genomes are shown in Supplementary Fig. S39.

To assess variant segregation across deeper cichlid phylogenies, we overlapped pairwise variants in highly accessible (HA) TFBSs in promoters of highly expressed (HE) genes of the five cichlids, with population SNP data from 73 Lake Malawi species (134 individuals) (Malinsky et al. 2018) and 21 other tilapia species (575 indviduals) (Ciezarek et al. 2024), respectively (see “*Methods*”). Notable divergent TF–TG pairs include MAF–*actr1*, SPDEF–*actr1*, and RELB–*actr1* in Lake Malawi's *M. zebra*; PBX1–*sws2* in the riverine and Lake Tanganyika species *A. burtoni*; and RUNX1–*rho*, MAF–*actr1*, LHX6–*actr1*, and DUXA–*actr1* in *O. niloticus*. Focusing on the photoreceptor gene *actr1*, which is under divergent selection in Lake Malawi's twilight zone (Hahn et al. 2017) and predicted to be regulated by the lens-associated (Anand et al. 2018) TF MAF, we found that the active MAF binding site is positionally conserved in *actr1* promoters of *O. niloticus* (Fig. 7b) and *M. zebra* (Fig. 7c), yet exhibits distinct segregating variants: within tilapia, the last motif position harbors a T/T allele conserved in *O. spilurus* and *O. aureus* but heterozygous or C/C segregating in 21 ecologically diverse tilapia species (Fig. 7d); while in Lake Malawi cichlids, the first motif position shows T/T conservation in 66 species but a homozygous A/A variant segregates in the deep benthic *Alticorpus macrocleithrum* (Fig. 7e–g, Fig. S39; see “Supplementary information”), a species occupying a specialized ecological niche, with unique sensory and morphological adaptations (Snoeks and Carvalho 2004).

Both variants are predicted to disrupt MAF binding (MotifDiff scores: tilapia −3.8, *A. macrocleithrum* −2.5; Fig. 7f; Tables S22 and S23), reducing the likelihood of canonical MAF regulation in these lineages. Conversely, closely related tilapias (*O. niloticus*, *O. spilurus*, and *O. aureus*) and most Lake Malawi species retain the ancestral motif and bind MAF, supporting conserved MAF-*actr1* photoreceptor regulation across habitats. The observed patterns support niche-specific divergence, whereby TFBS variants in *actr1* are associated with either clade-level conservation or ecological specialization within the cichlid radiation. Phylogenetic independent contrast (PIC) analysis shows minimal correlation change after accounting for phylogeny (Fig. S40; see “Methods”), reinforcing that segregating variants could reflect true lineage and niche adaptation.

In summary, functionally active TFBSs in visual system genes segregate by both phylogeny and ecological niche across radiating and nonradiating cichlid lineages, establishing active TFBSs as strong candidates for linking genomic variation to adaptive traits in natural populations. Notably, distinct TFBS variants within conserved regulatory elements—such as at the *actr1* promoter—recur in ecologically specialized species across a broad taxonomic sample (73 Lake Malawi species, 21 tilapias; Fig. 7d–f, Figs. S38 and S39), suggesting that regulatory variation could be a key contributor to adaptive innovations and rapid phenotypic diversification in East African cichlids.

## Discussion

East African cichlids provide a uniquely powerful system in which to investigate how genome-wide regulatory evolution shapes phenotypic diversification within a natural vertebrate radiation. By combining multi-tissue ATAC-seq, TF footprinting, comparative sequence analyses, and motif-supported GRNs across five species, this study builds on previous genomic, transcriptomic, and epigenomic work in cichlids (El Taher et al. 2021; Mehta et al. 2021, 2022, 2023; Vernaz et al. 2021, 2022), to reveal how chromatin accessibility, nucleotide changes at active regulatory elements and TF binding landscapes jointly evolve across genomes, tissues, and lineages. These findings demonstrate that the processes often inferred from locus-specific studies—cis-regulatory sequence evolution, TFBS turnover, and GRN rewiring—operate pervasively at the genome scale during rapid diversification in this radiation, providing examples of how evolution of regulatory architecture can underpin lineage-specific phenotypes.

Evolutionary change in noncoding regulatory sequences is increasingly recognized as a central driver of phenotypic diversification, particularly during rapid radiations (Kratochwil et al. 2018; Mehta et al. 2021, 2022; Urban et al. 2021; Vernaz et al. 2021, 2022). Our findings support and extend this view in cichlids, revealing a conserved regulatory core alongside pronounced lineage- and tissue-specific innovations. Thousands of promoter-associated accessible sites are conserved across all five species and four tissues, yet over half of these shared promoters exhibit signatures of accelerated nucleotide evolution, indicating that even constrained elements are frequently modified or co-opted in some lineages—paralleling observed patterns in other vertebrates (Dukler et al. 2020; Kuderna et al. 2024; McDonald and Reed 2024). In contrast, a smaller subset of promoter peaks and associated genes, especially in sensory tissues such as the retina, display strong lineage-specific accessibility and functional enrichment for visual system processes, suggesting that tissue-specific regulatory innovations at a flexible periphery are tightly linked to key adaptive traits in this radiation (Salzburger 2018).

### TFBS Turnover and GRN Rewiring as Mechanisms of Adaptive Evolution

Most TFBSs in promoters of 1-to-1 orthologous genes are not perfectly conserved in sequence or position, showing high turnover along the phylogeny. The *actr1* photoreceptor gene illustrates this with independent substitutions in different phylogenetic groups altering predicted MAF TF binding probabilities in patterns consistent with niche-specific shifts. Such variation offers a mechanism for GRN rewiring, whereby changes in regulatory connectivity, i.e. TF–TG relationships, alter transcriptional outputs and potentially adaptive phenotypes, consistent with previous functional studies in cichlids (Kratochwil et al. 2018, 2022; Mehta et al. 2021, 2022; Urban et al. 2021). Because TFBS annotations here rely on experimental footprinting of vertebrate motifs from several reference databases (Kulakovskiy et al. 2013; Hume et al. 2015; Yevshin et al. 2017; Rauluseviciute et al. 2024), turnover patterns imply potential evolutionary novelty that now needs functional validation; complementary mobility shift (Mehta et al. 2021) and high-throughput reporter (D'Elia and Bass 2025) assays will be essential to confirm their regulatory significance. No ChIP-seq data exist for these TFs in any of the five cichlid species; our footprint calls are supported instead by the Tn5-bias-corrected benchmarking against ChIP-seq in human and mouse (Li et al. 2019), prior EMSA validation of the NR2C2-*sws1* interaction in this system (Mehta et al. 2021), and, more broadly, by evidence from nonvertebrate systems that convergent phenotypes can arise from largely nonconserved underlying regulatory sequence (Van Belleghem et al. 2023), consistent with the pervasive TFBS turnover reported here. Together, these observations indicate that evolution of TF binding landscapes at active promoters provides a modular mechanism for altering regulatory connectivity and gene expression in cichlids.

Integrating chromatin and transcriptomic profiles, we find that gene promoter accessibility correlates significantly—though often weakly—with gene expression in most within species–tissue combinations, consistent with previous work in *O. niloticus* gill tissue (Mehta et al. 2023). This is expected, as accessible regions can mark not only active but also repressed or poised genes (Ackermann et al. 2016; Starks et al. 2019); confirmation of specific regulatory states will require complementary histone mark data (e.g. H3K27me3, H3K4 methylation) (Daugherty et al. 2017). Our analyses are necessarily promoter-centric: intronic and distal peaks show broadly similar cross-species accessibility patterns, but assignment to specific target genes is currently lower resolution and will benefit from future 3D chromatin-contact maps. Even so, HA promoters of HE genes are enriched for functions such as retinal physiology, metabolism, and oxygen transport—processes linked to coordinated evolution of visual systems and ecology in Lake Malawi species (Garcia et al. 2025)—linking chromatin divergence not only to molecular evolution but also to observed ecological and morphological diversification at the level of candidate regulatory networks.

Our GRN analyses extend these observations by showing that motif-supported network inference captures a more resolved view of regulatory divergence. Compared with expression-only networks, incorporating promoter TF footprints reduced network complexity and increased the interpretability of species-specific rewiring while retaining tissue-dependent patterns of conservation and divergence. This approach prioritized candidate TF–target relationships supported by both transcriptional evidence and accessible chromatin, rather than coexpression alone, providing a stronger basis for identifying direct regulatory changes likely to underlie adaptive phenotypes. In particular, the enrichment of rewired high-accessibility, high-expression edges in all tissues suggests that network-level regulatory changes are associated with ecological and functional diversification in cichlids e.g. retinal morphology (Carleton 2009) and liver metabolic processes (Vernaz et al. 2021). These findings build on our earlier coexpression (Mehta et al. 2021) and *in silico* motif prediction (Mehta et al. 2021, 2022)-based GRN studies by adding empirical epigenetic support for inference of regulatory interactions, highlighting how promoter accessibility and TFBS turnover could jointly shape lineage- and tissue-specific GRN evolution in a complex vertebrate system.

While retina tissue was prioritized here because of its well-characterized role in cichlid sensory adaptation and clear ecological light gradients (Carleton 2009), parallel analyses across forebrain, liver, and testis datasets reveal broadly comparable patterns of tissue-specific divergence and chromatin–expression relationships. These datasets now provide a basis for analogous, trait-focused studies in other tissues, for example examining whether divergence in forebrain regulatory regions predicts differences in brain structure and behavior (Patil et al. 2021), or whether liver regulatory changes track dietary shifts (Fages et al. 2026). In this way, the multitissue atlas established here can anchor future work on how tissue-specific regulatory evolution contributes to ecological and phenotypic novelty across the cichlid radiation.

### Implications for Studying Regulatory Evolution in Natural Vertebrate Populations

Although our study focuses on five focal species and four tissues, the framework we establish—combining multitissue chromatin accessibility, TFBS mapping and motif-supported GRNs in a natural radiation—can be readily extended to additional cichlid lineages and other vertebrate groups. Despite presenting the most comprehensive cross-species view of chromatin accessibility and regulatory connection evolution in cichlids to date, some caveats remain. To maximize physiological equivalence across species for these cross-species comparisons, we used lab-reared individuals and, where possible, controlled developmental stage by selecting sexually mature adults in comparable breeding condition, dissected and processed using an identical protocol, with replicates from the same family and similar age. Differences in maturation timing and coloration among species make precise matching difficult, and we cannot exclude effects of environment or captive breeding on genome dynamics, particularly in forebrain and reproductive tissues. However, all five species were reared under standardized conditions comparable to those used in other major genomic and transcriptomic studies in the same taxa (Brawand et al. 2014; Mehta et al. 2021, 2022). With these established protocols, future work can now expand taxon sampling beyond the five focal species, which were selected based on genomic resources (Brawand et al. 2014; Conte and Kocher 2015; Conte et al. 2019), phylogenetic coverage as representative lacustrine and riverine species (Brawand et al. 2014), and ecological interest (Salzburger 2018), as well as include both sexes and key developmental stages to track regulatory dynamics over time. Although sampling bias was minimized through careful tissue matching, high cell numbers (∼50,000 nuclei per replicate), and stringent peak reproducibility (IDR<0.1), undetected subpopulation differences are possible. In this respect, single-cell joint RNA and chromatin profiling (Ma et al. 2020), combined with emerging models of cis*-*regulatory syntax (Pampari et al. 2024), will be important for resolving cell type-specific GRNs and their evolutionary trajectories across lineages.

Further, we acknowledge that some open chromatin peaks classified as species-specific may reflect unresolved alignment of orthologous regulatory regions, local assembly gaps, or lineage-specific indels rather than true gain or loss of regulatory activity. While 1-to-1 ortholog filtering, IDR-based peak calling, and peak-overlap thresholds can reduce these effects, they cannot fully exclude them, so species-specific peaks should be viewed as strong candidates for regulatory novelty rather than unambiguous proof of it. Consistent with this caveat, our open chromatin peak calling versus assembly-quality analysis (see “*Methods*”) showed that species with higher-N50 assemblies, notably *O. niloticus* (Conte et al. 2017, 2019) and *M. zebra* (Conte and Kocher 2015; Conte et al. 2019), had fewer gap-associated peaks in the raw comparison (Fig. S41), but the adjusted analysis suggested that this pattern largely reflects lower gap burden in the more contiguous assemblies rather than a strong N50 effect independent of gaps (Fig. S42). Importantly, the absolute proportion of peaks overlapping gap-masked sequence was low overall, indicating that for most peaks in this study, assembly state is unlikely to be a major driver of the observed lineage-specific chromatin landscape; nevertheless, these results underscore the importance of telomere-to-telomere assemblies for representative cichlid species to further reduce ambiguity in interpreting regulatory novelty. This is especially relevant in rapidly evolving noncoding regions, where sequence divergence can obscure orthology even when regulatory function is conserved, as shown in recent vertebrate CRE comparisons (Phan et al. 2025).

Since matching species and tissues are unavailable in published methylomes (Vernaz et al. 2021, 2022) and transcriptomes (El Taher et al. 2021), we have been unable to integrate our atlas with these datasets reliably. Future integration of comparable data could nevertheless enable preliminary testing for anticorrelated marks associated with gene repression, while mapping transposable element insertions within accessible chromatin could help assess their contribution to regulatory novelty (Almeida et al. 2025). Expanding this framework across Lake Malawi cichlid clades, including *A. calliptera*, mbuna, benthics, *Diplotaxodon*, and *Rhamphochromis*, now supported by chromosome-level genome assemblies (Blumer et al. 2025), will enable testing of whether GRN innovations identified here and elsewhere (Mehta et al. 2021, 2022), together with segregating structural changes (Penso-Dolfin et al. 2020; Blumer et al. 2025), particularly of sensory systems (Blumer et al. 2025), could underpin fine-scale ecological diversification.

Overall, our results demonstrate the patterns of promoter accessibility, accelerated sequence evolution, and TFBS turnover that likely shape phenotypic innovations in East African cichlids. By moving beyond cataloging sequence change to directly measure and annotate regulatory activity, albeit primarily in gene promoter regions, we provide a mechanistic link between cis*-*regulatory evolution and genes associated with adaptive traits in both radiating and nonradiating cichlid lineages. This framework sets the stage for future work disentangling adaptive change from relaxed evolutionary constraint, and for functional dissection—using, for example, high-throughput reporter (D'Elia and Bass 2025) and CRISPR assays (Clark et al. 2023)—of how specific regulatory changes drive phenotypic outcomes. More broadly, it illustrates how integrative regulatory genomics can be used to connect noncoding variation to adaptive diversification in natural vertebrate populations.

## Materials and Methods

### Tissue Dissection

All animal procedures were approved by the relevant university and carried out in accordance with approved guidelines. Male *M. zebra, P. nyererei* (Makobe)*, A. burtoni*, *Neolamprologous brichardi*, and *Oreochromis niloticus* individuals (Table S1) were killed according to either Home Office (UK) or cantonal veterinary permit nr. 2317 (Switzerland) schedule 1 killing using overdose of MS-222 (tricaine) at The University of Hull, UK; University of Stirling, UK; and University of Basel, Switzerland. Four tissues (brain—forebrain specifically, eye—retina specifically, liver and testis) were dissected from each individual for DNA and RNA extraction. Selection of the four tissues enabled the study of tissue-specific associated traits under natural and/or sexual selection in cichlids: forebrain (behavior, learning and memory, and social interaction); retina (adaptive water depth/turbidity vision); liver (hepatic function associated with dietary differences); and testis (sexual systems associated with behavior).

### Cell Preparation

From a pool of all tissue cells, around 50,000 random cells were harvested, nuclei counted and then spun at 500×g for 5 min at 4 °C. Cells were washed once with 50 μL of ice-cold 1× PBS buffer and pelleted at 500×g for 5 min at 4 °C. Cell pellets were gently resuspended in 50 μL of 0.05% cold lysis buffer (10 mM Tris-HCl pH7.4, 10 mM NaCl, 3 mM MgCl_2_, 0.05% IGEPAL CA-630), and immediately pelleted at 500×g for 10 min at 4 °C and then stored on ice.

### Transposition Reaction and Purification

Pelleted nuclei were gently resuspended in a 50 μL transposition reaction mix composed of 25 μL 2× TD Buffer (Illumina), 2.5 μL Tn5 Transposes (Illumina), and 22.5 μL nuclease-free H_2_O. Transposition reaction was incubated at 37 °C for 30 min and purified using the PCR purification MinElute Kit (QIAGEN), according to manufacturer's protocol. Transposed DNA was eluted in 10 μL Elution Buffer (10 mM Tris buffer, pH 8) and stored at −20 °C.

### PCR Amplification

Transposed DNA was amplified in a final volume of 50 μL composed of 10 μL transposed DNA, 10 μL nuclease-free H_2_O, 2.5 μL 25μM P7 adapter, 2.5 μL 25μM P5 adapter, and 25 μL NEBNext high-fidelity 2 × PCR Master Mix (NEB). Transposed DNA was amplified for 5 min at 72 °C, 30 s at 98 °C, and 11 cycles of 10 s at 98 °C, 30 s at 63 °C, and 1 min at 72 °C in a PCR thermocycler. Amplified transposed DNA was purified using the PCR purification MinElute Kit (QIAGEN) and eluted in 20 μL Elution Buffer (10 mM Tris buffer, pH 8), according to manufacturer's protocol. To remove excess primers for final ATAC libraries, an additional 1× Agencourt AMPure XP bead (Beckman Coulter*)* clean-up was performed and eluted in 0.1× filtered TE. ATAC libraries were quantified on the Qubit 4 fluorometer (Invitrogen) and size distribution assessed on Agilent Tapestation and/or Bioanalyser.

### DNA Extraction

DNA was purified from the pool of all cells of each tissue using the DNeasy Blood and Tissue kit (QIAGEN) according to manufacturer's protocol. DNA was quantified on the Nanodrop 2000 (Thermo Scientific) and Qubit 4 fluorometer (Invitrogen) and used 1 ng for DNA library preparation using the Nextera XT DNA Library Preparation kit (Illumina), according to manufacturer's protocol. Library size distribution was assessed on Agilent Tapestation and/or Bioanalyser. DNA libraries, obtained from naked DNA, were used as internal controls to determine background levels of genomic DNA accessibility and Tn5 transposase sequence cleavage bias.

### ATAC and Control DNA Sequencing

51 ATAC and 51 corresponding naked DNA control libraries (Table S1) were equimolar pooled and 50 bp paired-end sequenced at Earlham Institute on two lanes of the Illumina NovaSeq 6000 platform using an S2 flow cell, generating an average of 61 million (ATAC-seq) and 16 million (naked DNA control) reads per library. Each species-tissue combination was profiled using independent biological replicates (distinct individuals; no technical replicates): two for *M. zebra, A. burtoni* and *N. brichardi*; three for *O. niloticus*; and three (retina) or four (forebrain, liver, and testis) for *P. nyererei* (Table S1). The variation in biological replicate number across species was owing to animal availability; this does not compromise the comparability of the resulting peak sets or downstream analyses. Two independent biological replicates are the minimum required by, and consistent with, the ENCODE ATAC-seq standard (https://www.encodeproject.org/atac-seq/) for IDR-based reproducibility filtering, which we applied uniformly to every species-tissue combination regardless of replicate number.

### ATAC-seq and Control DNA Processing

Sequence adaptors were removed and trimmed for quality from all raw paired-end reads using Trim Galore! (v0.5.0; https://github.com/FelixKrueger/TrimGalore) and FastQC (v0.11.9) (Andrews 2010) using default parameters. Read alignment, postalignment filtering, and ATAC peak calling were performed according to the ENCODE projects “ATAC-seq Data Standards and Processing Pipeline” for replicated data (https://www.encodeproject.org/atac-seq/). To minimize false inflation of species-specific regulatory peaks arising from divergent or repetitive sequence, we stringently mapped trimmed reads to their respective genome—*M. zebra* UMD2a (Conte and Kocher 2015; Conte et al. 2019); *P. nyererei* v1 (Brawand et al. 2014); *A. burtoni* v1 (Brawand et al. 2014); *N. brichardi* v1 (Brawand et al. 2014); *O. niloticus* UMD_NMBU (Conte et al. 2017, 2019) using bowtie2 (v2.2.6) (Langmead and Salzberg 2012), with parameters “–k 4 –X2000 –mm”, retained only high-confidence, properly paired mappings following the ENCODE ATAC-seq pipeline, and outputted in BAM format using SAMtools (v1.9) (Danecek et al. 2021). Since ATAC-seq can generate a high proportion (15% to 50% in a typical experiment) of mitochondrial-mapped reads, any reads mapping to their respective mitochondrial genomes were identified using BLAST (v2.3.0) (Camacho et al. 2009) and removed from the BAM file using SAMtools (v1.9) (Danecek et al. 2021). The resulting BAM files were sorted, and duplicated reads were marked using Sambamba v0.6.5 (Tarasov et al. 2015). Duplicated, unmapped, nonprimary alignment, and failing platform QC reads were filtered using SAMtools (v1.9) (Danecek et al. 2021), retaining reads mapped as proper pairs, and fragment length distributions were plotted using Picard (v1.140; https://github.com/broadinstitute/picard/). At each step, the recommended parameters from the ENCODE pipeline were applied. BAM files were converted to *tagalign* files using Bedtools (v2.30.0) (Quinlan and Hall 2010), and Tn5 shifting of ATAC mappings were carried out prior to peak calling. Peaks were identified using macs2 (v2.1.1) (Zhang et al. 2008; Feng et al. 2012) with the shifted tag as test and corresponding control DNA as input with parameters “-f BED -p 0.05 --nomodel --shift −75 --extsize 150”. Narrow peaks were used to create coverage tracks using *bedClip* and *bedToBigBed* in the UCSC tools package (v333; http://hgdownload.cse.ucsc.edu/admin/exe/). Following the ENCODE pipeline, irreproducible discovery rate (IDR) peaks of true replicates were flagged as either true (<0.1) or false (≥0.1) using idr v2.0.4 (https://github.com/kundajelab/idr). The fraction of reads in peaks (FRiP) was calculated using Bedtools (v2.30.0) (Quinlan and Hall 2010) and demarcated as pass (>0.3) or acceptable (>0.2) according to ENCODE guidelines. FRiP was not used as a QC measure, and instead, TSS enrichment was calculated using ATACseqQC (v1.18.0) (Ou et al. 2018), with the TSS enrichment QC requirement of a significant signal value at the center of the distribution being applied. All samples passed this criterion for further analysis, with TSS enrichment scores (mean depth within 1 kb of TSSs relative to flanking 100 bp regions) of 8.94 to 14.36 across all 51 libraries, within the ENCODE “ideal” range (>7; per-library scores in Table S1), and fragment-length distributions showing the expected nucleosome-associated periodicity (nucleosome-free, ∼<100 bp; mono-, ∼180 to 250 bp; di-, ∼350 to 450 bp; tri-nucleosome, ∼550 to 650 bp fragments, recurring at ∼180 to 200 bp intervals; representative examples in Fig. S1b). To avoid naïve approaches of merging peaks across replicates (Newell et al. 2021), for each species sample, a consensus set of peaks across all biological replicates was created based on reproducible (IDR true) peaks, where intersecting peaks are also ranked based on MACS2 peak significance score (Newell et al. 2021). Consensus peaks were used for subsequent analyses.

### Assessing Peak Calling Bias Versus Assembly Quality

We assessed whether open chromatin peak calling was biased by the aforementioned genome assembly quality by summarizing peak-level statistics across the four tissues for each species. Peaks were counted as gap-associated in genome contigs/scaffolds only if at least 10% of the peak bases were overlapping “N/n” gap regions, and as scaffold-end proximal if they fell within 100 bp of either scaffold end. For each species, these fractions were averaged across tissues, then merged with species-level assembly contiguity (scaffold/contig N50) (Krauss et al. 1991; Brawand et al. 2014; Conte and Kocher 2015; Conte et al. 2017) and genome-wide “gap fraction”, calculated as the proportion of all bases in the genome FASTA that were N/n's. We then tested the association between contiguity and peak bias using Spearman correlation and repeated the analysis with a partial Spearman correlation controlling for “gap fraction” to assess whether any N50 effect was independent of overall gap burden.

### Multiple Genome Alignment of Five Cichlid Species

A reference-free multiple genome alignment (MGA) of the five cichlid genomes (same versions as above) was created using Cactus (v2.0.3) (Armstrong et al. 2020) and outputted in multiple alignment format (MAF). This reference-free approach allowed us to define orthologous genomic regions across species, so that cross-species peak comparisons were made only within homologous loci rather than by single-reference coordinate transfer. Species-specific peaks were therefore called only after orthology-aware comparison of aligned regions, reducing the likelihood that apparent species specificity was driven solely by local assembly or mapping artefacts.

### Identifying CNEs

A neutral substitution model was created using the *phyloFit* function of PHAST (v1.5) (Hubisz et al. 2011) by fitting a time-reversible substitution “REV” model and parameters “—tree ‘((((Metriaclima_zebra,Pundamilia_nyererei), Astatotilapia_burtoni), Neolamprologus_brichardi), Oreochromis_niloticus)’ --subst-mod REV”. The five cichlid MGA was split by *O. niloticus* chromosome/scaffold using the *mafSplit* function in the UCSC-tools package (v333; http://hgdownload.cse.ucsc.edu/admin/exe/).

The neutral substitution model and MGAs of each *O. niloticus* reference chromosome/scaffold were used as input to predict conserved noncoding elements (CNEs) using the *phastCons* function of PHAST (v1.5) (Hubisz et al. 2011) with parameters “--target-coverage 0.3 --expected-length 30 --most-conserved --estimate-trees --msa-format MAF”. We used the conservation score output to define hCNEs with sequence identity ≥90% over ≥30 bp and pseudo accelerate/diverged CNEs (pseudo aCNEs) with sequence identity <90% over ≥30 bp. The evolutionary conservation-acceleration (CONACC) score of each pseudo aCNE was predicted by running *phyloP* (Pollard et al. 2010) of the PHAST (v1.5) package (Hubisz et al. 2011) using the likelihood ratio test (LRT) “--method LRT” on the CNE --features with their corresponding neutral substitution model in “--mode CONACC”. Pseudo aCNEs with a negative CONACC score, likelihood ratio <0.05, and significant divergence (altsubscale >1) were defined as significantly deviating from the neutral model, and therefore as true aCNEs. Since CNEs were defined by their position in *O. niloticus* chromosomes as a reference, corresponding CNE coordinates in the other species were defined using a combination of the *mafsinRegion* function of the UCSC-tools package (v333; http://hgdownload.cse.ucsc.edu/admin/exe/) and Bedtools (v2.30.0) (Quinlan and Hall 2010).

### Peak Annotation and Orthology

Up to 5 kb gene promoter regions were annotated in our previous study (Mehta et al. 2021) for *P. nyererei* v1, *A. burtoni* v1, and *N. brichardi* v1, with the same method being applied to the *M. zebra* UMD2a and *O. niloticus* UMD1 genomes in this study. We defined ±100 bp of TSS as the “core promoter” and up to 5 kb upstream as the “extended promoter” based on previously observing a plateau of predicted TFBSs from 5 to 20 kb upstream of the TSS in the same five species (see Fig. S22 in Additional File S1 (Mehta et al. 2021)). Narrow peaks either overlapping or most proximal to all annotated features in each genome were mapped using the *intersect* function of Bedtools (v2.30.0) (Quinlan and Hall 2010). Peaks not overlapping any gene's promoter region were excluded from this promoter-focused analysis (Fig. 1b, Table S2), which is distinct from the genome-wide, single-dominant-feature annotation of all consensus peaks—regardless of promoter overlap—presented separately (Fig. 1c, Fig. S2, Table S3). A presence–absence matrix was generated based on peaks overlapping in the up to 5 kb gene promoter regions for each species tissue, and each peak was assigned to a gene accordingly. Gene family relationships/orthologs of all genes in the five species were derived using OMA (v2.3.0) (Altenhoff et al. 2019) with Ensembl gene annotations corresponding to the genome versions provided above as input. A total of 38,445 orthologous genes were identified across the five species, with 13,889 1-to-1 orthologous genes found in all five species. Each “gene assigned peak” was then assigned to its corresponding orthologous gene, and for each species tissue, a single representative “best” peak (over all replicates) was assigned to each orthologous gene based on being (i) a True IDR peak; (ii) closest to the gene TSS; and (iii) most significant merged peak false discovery rate (FDR)-corrected *P-value* (*q-value*). Peak orthology in each species tissue was then determined using the above-derived orthologous genes and used for peak conservation or uniqueness, as well as gain or loss analysis below.

### Peak Gain or Loss and Genetic Diversity

Up to 5 kb gene promoter regions of species genes in all 38,445 orthologous genes were aligned using MAFFT v7.271 (Katoh and Standley 2013, 2016). Species peak positions were adjusted according to multiple alignment gaps, and overlapping peaks were determined using Bedtools *intersect* (v2.30.0) (Quinlan and Hall 2010). All pairwise overlapping peak regions, defined as “conserved” peaks, were required to encompass both species peaks summit, and nonoverlapping “unique” peaks as not, for analysis of conservation or uniqueness, gain or loss, and subsequent analysis of genetic diversity. For subsequent analyses, we only include *O. niloticus* as the reference and not the comparative species for loss since it is the base of our phylogenetic tree. To avoid any biases based on difference in genome quality and annotation, multiple alignments of 13,889 1-to-1 orthologous genes were used to calculate pairwise sequence similarity of “conserved” overlapping peak regions using an in-house python script (https://github.com/tmehta12/ATAC_bioinformatics) and categorized into bins of 0% to 1% (highly conserved), >1% to 5% (moderately conserved), >5% to 10% (less conserved) and >10% (variable) genetic diversity. These sequence-similarity thresholds reflect the spectrum of selective pressures observed across the five species' genomes, ranging from highly conserved coding regions (95 to 99.7% identity) to more variable whole-genome sequences (85 to 89% identity) (Mehta et al. 2022). For each reference peak within these sets, any pairwise absence of peak overlap is recorded as a loss. For example, a reference peak in *M. zebra* with overlapping peaks and summits in *P. nyererei* and *N. brichardi*, but not *A. burtoni* is recorded as presence between *M. zebra—P. nyererei* and *M. zebra—N. brichardi*, but a loss between *M. zebra—A. burtoni*. To determine species-specific peak gain in *M. zebra, P. nyererei*, *A. burtoni*, or *N. brichardi*, we calculated based on two sets of data: (i) a stringent criterion of only zero pairwise peak overlap; and (ii) where there is zero pairwise peak overlap or where either one or both species peak summits are not encompassed within the peak overlap region. Gains or losses and genetic diversity of peak overlap were then collated and counted for each tissue.

Genetic diversity in peak-adjacent regions across five species was quantified using a custom Python script (https://github.com/tmehta12/ATAC_bioinformatics) applied to 1-to-1 orthologous promoter alignments spanning ±500 bp from tissue-specific peak boundaries, enabling comparison of diversity in peak versus peak-adjacent regions. Diversity estimates were obtained through resampling, with 100 random lines drawn 50 times for peak-adjacent regions of each species tissue, and mean percentage nucleotide identity was calculated per iteration.

### Measuring Evolutionary Rate of Peaks

The evolutionary conservation-acceleration (CONACC) of each annotated *O. niloticus* peak was predicted by running *phyloP* (Pollard et al. 2010) in the PHAST (v1.5) package (Hubisz et al. 2011) using the likelihood ratio test (LRT) “--method LRT” on the peak --features with their corresponding neutral substitution model in “--mode CONACC”.

### Testing the Significance of CONACC Scores of Peaks Overlapping Genomic Regions

The significance of accelerated CONACC scores of annotated *O. niloticus* peaks in each genomic region was tested (Wilcoxon rank sum) against one another for (i) all peaks and (ii) within each pairwise comparison. The *P-*value was adjusted using Benjamini–Hochberg (BH) (Benjamini and Hochberg 1995) correction and recorded as either having a significant (Wilcoxon rank sum test, adjusted *P-*value <0.05) or insignificant (Wilcoxon rank sum test, adjusted *P-*value > 0.05) difference in accelerated CONACC scores for peaks in each region.

### Gene Ontology (GO) Enrichment

GO enrichment analyses of peaks (from 1-to-1 orthologous genes) localized to the up to 5 kb gene promoters for each tissue collated by species were conducted using the “g:GOst” module of g:Profiler (https://biit.cs.ut.ee/gprofiler/gost) (Raudvere et al. 2019), version e111_eg58_p18_f463989d (2024), using the species-specific databases. We use the FDR corrected hypergeometric *P* value with Benjamini–Hochberg (BH) (Benjamini and Hochberg 1995) correction to assess enrichment of GO terms, with a statistical cutoff of FDR <0.05.

### TF Footprinting, Enrichment, and Clustering

TF footprints were characterized using HINT-ATAC in the Regulatory Genomic Toolbox (v0.13.0) (Li et al. 2019) using a stringent false positive rate of 0.0001, with both species-specific and cichlid-wide PWMs as defined in our previous study (Mehta et al. 2021), as well as vertebrate PWMs from JASPAR (v9.0) (Castro-Mondragon et al. 2022; Rauluseviciute et al. 2024), HOCOMOCO (Kulakovskiy et al. 2013), GTRD (Yevshin et al. 2017), and UniPROBE (Hume et al. 2015).

The enrichment of accessible peaks in genomic regions was tested using the Genome Association Tester (GAT) tool (Heger et al. 2013). The accessible peaks were provided as segments of interest to test, with a bed file of annotations to test against, and workspace as the length of each scaffold/chromosome. GAT was ran using the following parameters: *–verbose = 5, −-counter = segment-overlap, −-ignore-segment-tracks, −-qvalue-method = BH –pvalue-method = norm*. We use the Benjamini–Hochberg (Benjamini and Hochberg 1995) FDR to assess enrichment of peaks in annotated regions, with a statistical cutoff of FDR < 0.05. Similarly, we use the FDR-corrected hypergeometric *P*-value (*q-*value) test to assess localized enrichment of TF footprint motifs in open chromatin peaks overlapping gene promoter regions. Enrichment is tested using a set-based approach whereby TF footprints in active gene promoter regions are compared against a background of TF footprints identified in open chromatin peaks in the whole genome.

For clustering TF activities, the mean of each TF's BS of motif match (as a measure of activity) in all gene promoters for all species and tissues was calculated. Using the mean bit score of each motif match, the within-group sums of squares and silhouette coefficient, as a measure of clustering homogeneity, for *k* = 1 to 20 clusters were calculated and plotted in R (v4.4.2). K-means clustering was selected over hierarchical clustering due to the volume of the data and to observe cluster differences between species, as opposed to a nested tree. The most suitable “k” for clustering was selected based on coherence between the two methods where (i) the sum of squared distances falls suddenly (“elbow” method) and (ii) the highest average silhouette width that is above 0.25, representative of structured data (Kaufman and Rousseeuw 1990). The bit scores were Z-score transformed across rows, and heatmap was plotted in R (v4.4.2) using the ComplexHeatmap package (v3.6) (Gu et al. 2016). TF footprint line plots were generated with the “differential analysis” module of HINT-ATAC in the Regulatory Genomic Toolbox (v0.13.0) package (Li et al. 2019) using bias-corrected signals.

### Nucleotide Variation in TF Motifs

Using a similar method for detecting peak genetic diversity above, TF motif positions were adjusted according to multiple alignment gaps of gene promoter regions, and overlapping TF motifs between *O. niloticus* (as a reference) and the other four species were determined using Bedtools *intersect* (v2.30.0) (Quinlan and Hall 2010). Overlapping TF motifs were classified as either 100% overlap or partial overlap, and whether they were the same or different TF motifs. An in-house python script (https://github.com/tmehta12/ATAC_bioinformatics) was used to identify all pairwise polymorphic sites in gene promoter alignments and using Bedtools *intersect* (v2.30.0) (Quinlan and Hall 2010), polymorphic sites overlapping *O. niloticus* TF motifs were identified with genomic coordinates of reference or alternate alleles recorded.

### RNA Extraction and Sequencing

RNA was purified from the remaining random pool of cells of each tissue using the RNeasy Plus Mini kit (QIAGEN) according to manufacturer's protocol. RNA and DNA content were quantified on the Qubit 4 fluorometer (Invitrogen) and integrity assessed on Agilent Tapestation and/or Bioanalyser, taking samples with RIN≥7 and <15% genomic DNA. A total of 48/51 (94%) samples passed these criteria for selection (Table S1). A total of 48 stranded RNA libraries were prepared using the NEBNext Ultra II Direction RNA-seq kit according to manufacturer's protocol. All stranded RNA-seq libraries were equimolar pooled and 150 bp paired-end sequenced at Earlham Institute on 1 lane of the Illumina NovaSeq 6000 platform using an S4 v1.5 flow cell, generating an average of 70 million reads per library.

### RNA-seq Processing

Read quality was assessed using FastQC (v 0.11.9) (Andrews 2010), and Trim Galore! (v 0.6.5; https://github.com/FelixKrueger/TrimGalore) was used to remove adapters and trim low-quality paired-end reads using default settings. All reads were then mapped to their respective genome—*M. zebra* UMD2a (Conte and Kocher 2015); *P. nyererei* v1 (Brawand et al. 2014); *A. burtoni* v1 (Brawand et al. 2014); *N. brichardi* v1 (Brawand et al. 2014); *O. niloticus* UMD_NMBU (Conte et al. 2017) using HISAT2 (v 2.2.1) (Kim et al. 2019) with default parameters. Mapping QC was carried out using QualiMap (v 2.2.1) (Okonechnikov et al. 2016) with default “rnaseq” parameters. The final BAM file was sorted using samtools (v1.16.1) (Danecek et al. 2021), and transcript abundance was calculated using “htseq-count” in the HTSeq (v 2.0.2) (Anders et al. 2015) package.

### Identifying ATAC Peak and Gene Expression Associations

To prioritize peak–gene relationships, we devised an approach to identify peaks that could regulate their target genes expression. For gene expression, we used an approach applied previously (Li and Dewey 2011; Mehta et al. 2023) to calculate transcript per million (TPM) values by first normalizing transcript counts by gene length, as calculated using GTFtools (Li et al. 2022), followed by the library size, as calculated using QualiMap (v 2.2.1) (Okonechnikov et al. 2016), and then *log2*(x + 1) transforming (i) each genes TPM in each replicate and (ii) the mean TPM for each gene across biological replicates. Each genes mean TPM across biological replicates was used to assess correlations with ATAC signal. The ATAC signal was processed using an approach applied previously (Sanghi et al. 2021; Mehta et al. 2023) to obtain the number of independent Tn5 insertions in collated gene promoter peaks. The number of insertion sites, i.e. peak counts, was counted in collated narrow peaks against a merged BAM file of all biological replicates using Bedtools (v2.30.0) *coverage* (Quinlan and Hall 2010). A peak count matrix was created and normalized using edgeR (Robinson et al. 2010) counts per million (CPM) “log = TRUE, prior.count=5”, followed by a quantile normalization using the preprocessCore (https://github.com/bmbolstad/preprocessCore) *normalize.quantiles* module in R (v 4.4.2). After merging the ATAC signal of biological replicates using the *log2* average from the normalized count matrices, the average *log2*-TPM and *log2*-ATACSignal for each gene was plotted with the correlation coefficient (*r*) calculated in R (v 4.4.2), using Spearman's rank correlation throughout, as it is robust to outliers and does not assume a linear relationship between accessibility and expression.

Using an approach devised previously (Starks et al. 2019; Mehta et al. 2023b), putative activated, repressed, or poised genes were categorized based on assigning each peak–gene relationship to one of four groups according to gene promoter accessibility (*log2*-ATACSignal) and gene expression (*log2*-TPM) values that are less than the 50th (medium-low) or more than the 70th (high) percentiles of all peaks counts or genes expression values in the same species tissue. The four groups are (1) HA–HE (high accessibility and high expression); (2) HA–ME (high accessibility and medium–low expression); (3) MA–ME (medium–low accessibility and medium–low expression); or (4) MA–HE (medium–low accessibility and high expression). To maintain stringent gene groups, any genes not falling into any of the four groups were discarded.

### Inference and Comparative Analysis of Tissue-specific Gene Regulatory Networks

GRNs were inferred for each species-tissue combination using GENIE3 (v1.30.0) (Huynh-Thu et al. 2010) on orthologous gene expression data, yielding TF–TG edges ranked by importance weight. To integrate ATAC-seq footprinting evidence, we filtered GENIE3 networks to retain only edges supported by orthologous motif footprints, extracting maximum motif BSs. Composite edge weights were calculated by first calculating normalized motif BSs (to match GENIE3's 0 to 1 range) followed by composite weight calculation:


$$\mathrm{Motif}\;\mathrm{Bit}\;\mathrm{Scorenorm}=\frac{\mathrm{Bit}\;\mathrm{Score}\;-\;\mathrm{Bit}\;\mathrm{Scor}e_{\min}}{\mathrm{Bit}\;\mathrm{Scor}e_{\max}\;-\;\mathrm{Bit}\;\mathrm{Scor}e_{\min}}$$

$$\begin{array}{rl} & \mathrm{CompositeWeight}\\ & =\text{GENIE3 Weight}{\;}^{\prime }\mathrm{MotifBitScor}e_{\mathrm{normalised}} \end{array}$$

This produced ranked edge lists for each of the species-tissue networks across the five species.

Comparative GRN analysis was performed in R (v4.5.0). For each tissue, the top 12,000 composite-weighted edges per species were selected for Jaccard similarity analysis across species pairs, generating tissue-specific similarity matrices and MDS ordinations. Rewiring was quantified per species by computing a composite score combining (i) species-specificity (binary: edge absent in other species), (ii) composite weight delta relative to orthologous edges, and (iii) rank advantage relative to orthologous edges, with top 100 rewired edges identified per species-tissue. All GRN regulators and targets were then classified into accessibility/expression categories (HA–HE, MA–HE, HA–ME, and MA–ME), and category enrichment was assessed using one-sided Fisher's exact tests against the complete set of rewired edges as background, with Benjamini–Hochberg (Benjamini and Hochberg 1995) FDR correction applied within each species-tissue across the four edge classes (FDR < 0.05). For downstream prioritization, the full distribution of composite weights and rewiring scores across edge categories was examined, and ranked HA–HE candidate edges by a combined score calculated as CompositeWeight × rewiring score.

### Identification of Segregating Variants in Binding Sites

Pairwise variants of *M. zebra* and *O. niloticus,* derived from gene promoter alignments above, were overlapped with SNPs in either Lake Malawi (Malinsky et al. 2018; Blumer et al. 2025) or other tilapia (Mehta et al. 2023b; Ciezarek et al. 2024) species respectively, using Bedtools (v2.30.0) *intersect* (Quinlan and Hall 2010). Variants were prioritized based on ordering TF–TG candidates where (i) the TG is HA–HE and a TFBS in the promoter has variation between species; (ii) a TFBS has variation between species; or (iii) the TG is HA–HE; and then ordering variants in TFBSs based on ranked reads (TC) in the footprint and BS of the motif match.

### Quantifying Variant Effect on Binding Site Disruption

The *M. zebra* UMD2a (Conte and Kocher 2015) and *O. niloticus* UMD_NMBU (Conte et al. 2017) genomes, HOCOMOCO v11 human motifs (in meme format), and chromosome-separated vcf files of Lake Malawi species (Malinsky et al. 2018; Blumer et al. 2025) or tilapia SNPs (Mehta et al. 2023b; Ciezarek et al. 2024) were inputted into *MotifDiff* (Hosseini et al. 2025) to obtain normalized scores of variant effect to either decrease (score of <0) or increase (score of >0) binding probability of all predicted motifs along each chromosome.

### Phylogenetic Independent Contrasts

PICs were carried out to test the effect of fitting species phylogenies to the covariance of segregating TFBSs and species ecologic traits derived from previous studies (habitat (Hofmann et al. 2009; Froese and Pauly 2026) and foraging habit/diet (Hofmann et al. 2009)). This involved (i) coding the genotypes of segregating regulatory sites and ecologic measurements for all species and (ii) using the ape package (v5.8.1) in R (v4.5.0) to apply the PICs test (Felsenstein 1985) on all correlations with the binding site genotype (genotype vs. habitat and genotype vs. foraging habit/diet). PICs assume a linear relationship and Brownian motion process between traits, and thus, for each combination of data, scatterplots were first generated. To test for any correlation changes owing to phylogenetic signal, the regression model was compared between the relationships both excluding and including the respective input species phylogeny.

## Supplementary Material

evag247_Supplementary_Data

## Data Availability

The data underlying this article are available in the article, its online supplementary material, or uploaded to Zenodo (https://doi.org/10.5281/zenodo.19821867), organized and named by species, tissue, and analysis stage to allow direct cross-referencing with new datasets. All code is available on GitHub at: https://github.com/tmehta12/ATAC_bioinformatics.
