# Supplementary material for: Genome-Wide Evolution of Lineage-Specific Chromatin Accessibility Links Regulatory Changes to Cichlid Phenotypic Diversity
Source: Genome Biol Evol. 2026 Oct 8;18(10):evag247. doi: 10.1093/gbe/evag247 (PMC13691606; doi:10.1093/gbe/evag247)
Supplement: Supplementary file 1 [file evag247_supplementary_data.zip › CichlidATAC_manuscript_SuppInfoFigs.pdf]

Supplementary Information

Results

Chromatin accessibility maps conserved and lineage-specific regulatory architecture across tissues

Across all species and tissues, an average of 78–99% of open chromatin peaks occur within 5 kb of a gene's transcription start site (TSS), with 10–20% located in the core promoter region (±100 bp of the TSS) (Fig. 1b, Supplementary Fig. S1, Supplementary Table S2). More specifically, peaks are most strongly enriched in 5′ UTR sequences across all species (fold enrichment = 1.8–4.1, adjusted $P < 0.05$; Fig. 1c). Genome wide, across all species and tissues, open chromatin peaks span several genomic features, overlapping intronic regions (40%), exons (23%), and intergenic regions (17%), with the remainder distributed among promoter regions within 5 kb upstream of the TSS (10%), 5′ UTRs (7%), 3′ UTRs (3%), and conserved noncoding elements (CNEs; 0.04%) (Supplementary Fig. S2, Supplementary Table S3).

Core regulatory elements underpin shared and tissue-specific functions

Using the 13,997 1-to-1 orthologous genes across the five species (see '*Materials and Methods*'), we identified 13,889 orthologous genes (99%) with an associated open chromatin peak in the gene promoter region (Fig. 1d). These orthologous genes are enriched for common biological processes such as cell differentiation and epithelium development in each of the four tissues, as well as unique processes such as synapse organisation (in forebrain tissue), axon extension (in retina tissue),

myotube differentiation (in liver tissue) and dephosphorylation (in testis tissue) (Supplementary Fig. S3).

In the 13,889 orthologous genes, a total of 5,536 (40%) unique orthologous genes (202-4321 orthologous genes in each tissue) have conserved gene promoter peaks in orthologous genes of all five species (*Anc1* node, Fig. 1d). Examples of which include in forebrain tissue (4321 orthologous genes), *irx2*, a gene associated with neural differentiation and behavioural helper care in a cooperatively breeding cichlid [1]. Examples in the retina tissue (202 orthologous genes) include *atxn1b*, a gene important for visual function [2], and with an overall gain of TFBSs along the same phylogeny [3], has significantly rewired GRNs in cichlids [4]. Along the phylogeny, 1,473 unique orthologous genes (65-1253 orthologous genes in each tissue) have shared peaks in gene promoters of the haplochromines (Anc3, Fig. 1d); in liver tissue (231 orthologous genes), this includes *foxm1,* a gene associated with cell cycle stimulation during liver regeneration [5]. Notably, compared to ancestral nodes, there are fewer (39-188) unique orthologous genes with species-specific peaks in their gene promoters, but variability (36-1762) of orthologous genes with peaks at the per tissue level across species (Fig. 1d); in forebrain tissue (71-513 orthologous genes), this includes *lrrtm1* in *A. burtoni* and *vldlr* in *P. nyererei* associated with modulating synaptic cell adhesion [6] and neural development [7] respectively; in retina tissue (36-1762 orthologous genes), this includes *fgf10* in *M. zebra* and *sws1* in *N. brichardi* that are associated with eye morphogenesis [8] and short wavelength sensitive visual function in cichlids [9] respectively; in liver tissue (73-380 orthologous genes), *cxcr3* in *M. zebra* and *cxcr1* in *O. niloticus,* genes associated with hepatic immune response [10,11]; and in testis tissue (88-439 orthologous genes), *plag1* in *P. nyererei* associated

with spermatogenesis [12], and *prdm9* in *A. burtoni* associated with meiotic recombination [13].

Conserved and species-specific promoter accessibility reveals tissue-specific regulatory variation

We then focus on the species-specific level where, compared to ancestral nodes (1018-5536 orthologous genes), there are fewer unique orthologous genes (39-188), but tissue-specific variability (36-1762 orthologous genes) of species-specific peaks in gene promoters (Fig. 1d). The number of gene promoter peaks in unique orthologous genes varies between species (Mz = 192-1762 orthologous genes; Pn = 73-413 orthologous genes; Ab = 36-439 orthologous genes; Mz = 88-344 orthologous genes; Mz = 304-513 orthologous genes) and tissues (Forebrain = 1302 orthologous genes; Retina = 2747 orthologous genes; Liver = 1124 orthologous genes; Testis = 1562 orthologous genes).

By calculating the standard deviation (SD), mean, and coefficient of variation (CV) of both 'conserved' ancestral (set 1) and species-specific (set 2) gene promoter peaks in orthologous genes, we identified that all tissues show higher variability of orthologous genes in set 1 (forebrain CV=169%; retina CV=162%, liver CV=155%; testis CV=66%) compared to their counterparts in set 2 (forebrain CV=71%; retina CV=122%, liver CV=50%; testis CV=44%) (Fig. 2a). However, peaks in gene promoters of retina tissue exhibit high variability in both sets, indicating dynamic evolution of chromatin accessibility in retina-related genes both ancestrally and at a species-specific level. In forebrain and liver, there is high variability of orthologous genes at ancestral nodes, but low-moderate variability at the species level, suggesting more dynamic evolutionary processes in forebrain-related and liver-

related genes at ancestral levels, but more conserved patterns of chromatin-accessibility at the species level. Testis on the other hand shows the lowest variability of orthologous genes amongst all tissues in ancestral nodes and relatively low variability at species-specific nodes, indicating more consistent evolutionary patterns of chromatin accessibility in testis-related genes across both ancestral and species-specific contexts.

This identified peaks in individual tissues of gene promoters potentially relevant to species-specific function; in forebrain tissue (71-513 orthologous genes), this includes *lrrtm1* in *A. burtoni* and *vldlr* in *P. nyererei* associated with modulating synaptic cell adhesion [6] and neural development [7] respectively; in retina tissue (36-1762 orthologous genes), this includes *fgf10* in *M. zebra* and *sws1* in *N. brichardi* that are associated with eye morphogenesis [8] and short wavelength sensitive visual function in cichlids [9] respectively; in liver tissue (73-380 orthologous genes), *cxcr3* in *M. zebra* and *cxcr1* in *O. niloticus,* genes associated with hepatic immune response [10,11]; and in testis tissue (88-439 orthologous genes), *plag1* in *P. nyererei* associated with spermatogenesis [12], and *prdm9* in *A. burtoni* associated with meiotic recombination [13].

Accelerated evolution of active gene promoters highlights hotspots of regulatory innovation in cichlids

To study the divergence of open chromatin peaks, whether conserved or diverged across tissues and species, we calculated the rate of nucleotide substitutions of each *O. niloticus* peak as a reference against the four other species, compared to a neutral model using phyloP [14] (see '*Materials and Methods*'). Within each annotation,

61-85% of *O. niloticus* open chromatin peaks are conserved with the other four species, whereas 7-35% exhibit accelerated evolution (Supplementary Fig. S5-S6 and Supplementary Table S6). Consistent with our previous findings of regulatory divergence in cichlid gene promoter regions [4,15], the highest proportion (35%) of open chromatin peaks in *O. niloticus* tissues exhibiting accelerated evolution along the phylogeny, are found to overlap gene promoter regions (Supplementary Fig. S5-S6 and Supplementary Table S6). Notably, the proportion of accelerated peaks are significantly different (Wilcoxon rank sum test, (Benjamini-Hochberg adjusted *p-value* <0.05) between annotations, especially between comparisons of gene promoter regions with the other annotations (5' UTR, Exon, Intron and 3' UTR) both within *O. niloticus*, and pairwise between *O. niloticus* and the other species (Supplementary Table S7). Within the 13,889 orthologous genes, 12,922 have *O. niloticus* open chromatin peaks that are localised to gene promoter regions and of which, 7,615 (59%) exhibit accelerated evolution in at least one lineage along the phylogeny. A total of 5,536 unique orthologous genes (202-4321 orthologous genes in each tissue) have open chromatin peaks in orthologous gene promoter regions of all five species (Anc1, Fig. 1d) of which, 3,061 (55%) exhibit accelerated evolution along the phylogeny. Overall, this lends further support to focus on open chromatin peaks overlapping gene promoter regions of 1-to-1 orthologous genes to 1) characterise divergence of tissue-specific peaks, including gains or losses and genetic diversity along the phylogeny and 2) study any open chromatin and transcriptional divergence correlations.

To examine the divergence of gene promoter peaks in the phylogeny, gene promoter peak gain and loss for each lineage was quantified and categorised according to

corresponding pairwise genetic diversity (Fig. 2e, Supplementary Table S8 and S9). Since there is no previous knowledge of expected divergence at active gene promoter sites in cichlid species, we use *O. niloticus* peaks as a reference where peak summits overlap in all pairwise comparisons (Supplementary Table S8, see *Materials and Methods*). In all comparisons against *O. niloticus*, *N. brichardi* has the most peak gains in retina (52% of all 13,889 1-to-1 orthologous gene promoter regions), liver (44%), and testis (48%) tissue. This is not biased by genome completeness or annotation quality, since *N. brichardi* has one of the fewest annotated protein-coding genes (23,568 – compared to 23,044-28,142 in the other four species [16-18]) and lowest genome contig N50 (13.2 kb – compared to 20kb - 3.1 Mb in the other four species [16-18]), whereas *O. niloticus*, used as the reference here for an unbiased approach, has the most complete genome (3.1 Mb) and highest protein coding gene count (28,142 protein-coding genes) [17].

In forebrain tissue, the most peak gains (45% of 1-to-1 orthologous genes) are instead observed in *M. zebra,* with an enrichment of tissue function, e.g., neurogenesis (Benjamini-Hochberg [19] adjusted *p-value* <0.05), with the fewest peak gains in general found in *A. burtoni,* e.g., 15% in forebrain (Supplementary Table S8), with unique functional enrichments, e.g., monatomic transmembrane transport (Benjamini-Hochberg [19] adjusted *p-value* <0.05), and possibly relevant to the *A. burtoni* model species for behaviour and social neuroscience [20]. Across all four tissues and pairwise comparisons, a total of 27-40% (*M. zebra*), 36-47% (*P. nyererei*), 19-38% (*A. burtoni*), and 30-47% (*N. brichardi*) of the 13,889 1-to-1 orthologous genes exhibit a loss of at least one gene promoter peak (Fig. 2e, Supplementary Table S8). Overall, peak loss in all species could be attributed to sequence divergence as 76-91% of peak loss regions present more than 10%

pairwise sequence divergence (Fig. 2e, Supplementary Table S8). Consistent with previous findings of regulatory site divergence [4,15], more than 10% sequence divergence over a mean peak size of 236 to 419 bp across all samples, could be indicative of the gain or loss of regulatory sites, e.g., TFBSs (mean size of 13 bp across all five species). We therefore conclude that increased nucleotide divergence at active gene promoter regions is associated with chromatin accessibility peak loss and could drive novel divergence of functionally active regulatory sites associated with tissue-specific traits e.g., the visual systems.

Transcription factor binding landscapes are extensively reshaped at active regulatory sites

To identify conserved and species- or clade-specific binding patterns, tissue-specific TF activity was clustered into co-active groups within each species (see *Materials and Methods*). We identified 13 (forebrain, n=1427 unique TF motifs; Supplementary Fig. S9), 10 (retina, n=1424 unique TF motifs; Fig. 3c), 8 (liver, n=1430 unique TF motifs; Supplementary Fig. S10), and 11 (testis, n=1427 unique TF motifs; Supplementary Fig. S11) clusters of binding patterns, with varying levels of orthologous TF binding activity observed across the five species. For example, in forebrain tissue, *prox1* associated with zebrafish neurogenesis [21], is highly activated in cluster 10 of *M. zebra* (Z-score of 1.7) but has low activity in the other species (Z-score of -0.8 to -0.2), whereas *otx2* associated with brain morphogenesis [22], is highly activated in cluster 1 of *A. burtoni* (Z-score of 1.8) but has low activity in the other species (Z-score of -0.6 to -0.3) (Supplementary Fig. S9). Varying activity is also observed in liver tissue, where *p73* associated with liver fuel metabolism [23] is highly activated in cluster 6 of *N. brichardi* (Z-score of 1.8 versus -0.7 to -0.2 in the other

species), and *runx3* associated with liver development [24] is highly activated in cluster 3 of *O. niloticus* (Z-score of 1.6 versus -1 to 0.2 in the other species) (Supplementary Fig. S10). This varying activity is also observed in testis tissue, where *pbx1* associated with testis-determination [25] is highly active in cluster 10 of *P. nyererei* (Z-score of 1.8 versus -0.6 to -0.3 in the other species) (Supplementary Fig. S8).

Dynamic regulatory site divergence is associated with regulatory rewiring in cichlids

Across the four tissues and species, 35-66% of pairwise polymorphic sites (in the promoter regions of 6842 – 8200 1-to-1 orthologous genes) map to *O. niloticus* TF motifs (Fig. 4b, Supplementary Table S14b) and thus, could impact TF binding activity. Of these, we define a 'principal set' where 1-5% polymorphic sites (1385-1439 non-redundant TF motifs in the promoter regions of 749-2747 genes) are in partially (< 100%) overlapping TF motifs between each species tissue and *O. niloticus,* where the majority (99.9%) of overlapping TF motifs are different (Fig. 4c, Supplementary Table S14c). Further, 6-13% of these polymorphic sites (175-1361 non-redundant TF motifs in the promoter regions of 18-618 1-to-1 orthologous genes) are in 'accelerated peaks' (Supplementary Table S14c), indicative of accelerated nucleotide divergence in actively bound TFs of the comparison species, relative to neutrally evolving sites in *O. niloticus*.

We observed concrete examples where motif-altering variants result in predicted switching of TF binding sites associated with genes linked to adaptive traits. In the accelerated set (of 24-769 genes), we identified examples of both discrete, e.g., one variant changing an IRF7 site to USF2 in the retinal gene, *pitpn* [26] (Fig. 4g), in *P.*

nyererei, and indiscrete nucleotide variation, e.g., seven variants changing a STAT1 site to TEAD4 in the liver development gene [27], *foxa2*, in *N. brichardi.* Altogether, both subtle and significant genetic variations in motifs of actively bound TFs have likely contributed to motif divergence and GRN rewiring of several genes linked to adaptive traits across the five species.

Regulatory divergence in chromatin accessibility associates with transcriptional changes in tissue-specific and adaptive genes

Upon investigating the correlation between accessible gene promoter regions, including divergent TF binding affinities, and transcriptional changes across species and tissues, we identified that in all five species retina tissue, peak-gene expression relationships were most abundant in the HA-HE category (37-41% of all categorised relationships), with 1408-4842 non-redundant peaks and 902-2020 non-redundant genes (Fig. 5a, Fig. S18-S22, and Supplementary Table S16). In the same tissue, the remainder are categorised as either HA-ME (929-3069 peaks and 588-1325 non-redundant genes: 23-24% of all relationships), MA-HE (1212-5862 peaks and 885-2487 non-redundant genes: 20-24% of all relationships), or MA-ME (790-4290 peaks and 542-1812 non-redundant genes: 15-16% of all relationships) (Fig. 5a, Fig. S18-S22, and Supplementary Table S16).

Functional variation at active TF binding sites tracks phylogeny and ecology within the cichlid radiation

We hypothesise that genetic variation between the five cichlid species and their respective lake or riverine species counterparts can identify functional variation in TFBSs of orthologous genes associated with niche- and/or clade- specific adaptive

traits that differ between the species. To prioritise 1-to-1 orthologous genes, we ranked TFBSs in promoter regions using the best described classification method [28], by selecting TFBSs with tag counts (TC, number of reads) and bit-score (BS) of motif match above the means for all statistically significant predicted TFBSs in each species tissue (Benjamini-Hochberg [19] adjusted *p-value* <0.0001) (see '*Materials and Methods*'). We started with 896,634-2,247,267 positionally non-redundant TFBSs in 93,139-226,058 footprints, characterised in the promoter regions of 13,774-21,553 genes of the five species retina tissues. In this set, we identified 108,193-354,906 (10-20%) predicted TFBSs in 18726-59883 (15-32%) footprints in the promoter regions of 6844-13886 (41-76%) genes above the mean TC and BS of all genes (Fig. 7a; Supplementary Table S21). This is comparable to that identified in *O. niloticus* gill tissue previously [29] and amongst these, we identified candidate transcription factor (TF) – target gene (TG) relationships where dynamic divergence of regulatory sites correlates with transcriptional divergence, concentrating on well-defined cichlid visual system genes that also strongly associate with niche diversity [30]. We then filtered for 13,997 1-to-1 orthologous genes, and then categorised TF-TG candidates where 1) the TG is HA-HE and a TFBS in the promoter has variation between species e.g., *O. niloticus* RUNX1-*rho*; 2) a TFBS has variation between species e.g., *P. nyererei* USF2-*pitpn*; or 3) the TG is HA-HE e.g., *N. brichardi* NR2F6-*sws1* (Fig. 7a).

We examined the photoreceptor gene, *actr1*, under divergent selection in Lake Malawi cichlids from the twilight zone [31], and predicted to be regulated by MAF, a transcription factor involved in lens development [32]. The active MAF binding site is conserved in the orthologous *actr1* gene promoter of *O. niloticus* (Fig. 7b) and *M.*

zebra (Fig. 7c) with segregating variants in the last (tilapia species, Fig. 7d) and first (a Lake Malawi species*,* Fig. 7e, Supplementary Fig. S38) positions of the binding site (Fig. 7f-g). The homozygous variant (T/T) in the last position is 1) conserved in the same clade species, *O. spilurus* and *O. aureus*; but 2) heterozygous or homozygous segregating in all 21 distantly related tilapia species with diverse salinity and habitat adaptations, for example, *O. urolepis* and *O. mossambicus* (both C/C) (Fig. 7d). Conversely, the homozygous variant (T/T) in the first position is 1) conserved with all 66 other Lake Malawi species; but 2) homozygous segregating in the deep benthic, *Atlicorpus macrocleithrum* (A/A, Fig. 7e and 6g, Supplementary Fig. S39), that occupies a niche utilising enlarged sensory pores on the lower head and a uniquely adapted elongated cleithrum on the chest [33]. This variant was confirmed by identifying the 1) *A. macrocleithrum* segregating variant in multiple reads [34,35] (Fig. 7g, Supplementary Fig. S39); and 2) corresponding *actr1* gene promoter and MAF TFBS region in chromosome-level genomes of representative Lake Malawi caldes (*A. calliptera, Tropheops* sp. 'mauve'*, Aulonocara stuartgranti,* and *Ramphochromis* sp. 'Chilingali') genomes [35] (Supplementary Fig. S39).

Both MAF TFBS variants are predicted to be disruptive, where MotifDiff scores of less than 0, indicate a significant decrease of binding probability [36] of MAF to the *actr1* gene promoter of 1) 21 tilapia species with diverse salinity and habitat adaptations (Fig. 7f, *MotifDiff* score of -3.8); and 2) the Lake Malawi deep benthic species, *A. macrocleithrum* (Fig. 7f, *MotifDiff* score of -2.5). Overall, this suggests that MAF 1) could be a core regulator of *actr1* in the closely related clade of *O. niloticus*, *O. spilurus* and *O. aureus* (Fig. 7d)*,* as well as most Lake Malawi species (Fig. 7e) where photoreception in diverse habitats, influencing feeding, social, and reproductive behaviour is vital for overall fitness; but 2) is unlikely to regulate *actr1* in

the rest of the tilapia phylogeny, as well as the uniquely adapted LM deep benthic species, *A. macrocleithrum* (Fig. 7), possibly indicating niche-specific divergence of photoreceptor regulation across cichlid phylogenies. Further, phylogenetic independent contrast (PIC) analysis [37] of the *actr1* (Supplementary Fig. S40) genotype against ecology of each of the 73 Lake Malawi species, highlights very little change in correlation once the phylogeny is taken into account and a regression model fitted (*see Materials and Methods*).

In summary, we identified active TF binding sites of visual system genes that segregate according to phylogeny and ecology of radiating and non-radiating cichlid fish species. The presence of distinct variants at two sites within a positionally conserved TFBS, segregating independently across both a non-radiating tilapia lineage and the rapidly radiating Lake Malawi cichlids, suggests that independent selective pressures may be acting on different positions within conserved regulatory elements in each lineage - indicative of lineage-specific adaptation even at conserved regulatory motifs. This highlights the role of regulatory variation TFBSs as a key contributor to adaptive innovations across East African cichlids.

References

1. Kasper, C., Hebert, F.O., Aubin-Horth, N. & Taborsky, B. Divergent brain gene expression profiles between alternative behavioural helper types in a cooperative breeder. *Molecular Ecology* **27**, 4136–4151 (2018).
2. Nishiguchi, K.M., Aoki, M., Nakazawa, T. & Abe, T. Macular degeneration as a common cause of visual loss in spinocerebellar ataxia type 1 (SCA1) patients. *Ophthalmic Genetics* **40**, 49–53 (2019).
3. Mehta, T.K. *et al.* Evolution of miRNA binding sites and regulatory networks in cichlids. *bioRxiv*, 2021.12.14.472604–2021.12.14.472604 (2021).
4. Mehta, T.K. *et al.* Evolution of regulatory networks associated with traits under selection in cichlids. *Genome Biology* **22**, 25–25 (2021).
5. Wang, X., Kiyokawa, H., Dennewitz, M.B. & Costa, R.H. The Forkhead Box m1b transcription factor is essential for hepatocyte DNA replication and mitosis during mouse liver regeneration. *Proceedings of the National Academy of Sciences* **99**, 16881–16886 (2002).
6. Soler-Llavina, G.J., Fuccillo, M.V., Ko, J., Südhof, T.C. & Malenka, R.C. The neurexin ligands, neuroligins and leucine-rich repeat transmembrane proteins, perform convergent and divergent synaptic functions in vivo. *Proceedings of the National Academy of Sciences* **108**, 16502–16509 (2011).
7. Tissir, F. & Goffinet, A.M. Reelin and brain development. *Nat Rev Neurosci* **4**, 496–505 (2003).
8. Govindarajan, V., Ito, M., Makarenkova, H.P., Lang, R.A. & Overbeek, P.A. Endogenous and ectopic gland induction by FGF-10. *Dev Biol* **225**, 188–200 (2000).
9. Spady, T.C. *et al.* Evolution of the cichlid visual palette through ontogenetic subfunctionalization of the opsin gene arrays. *Mol Biol Evol* **23**, 1538–47 (2006).
10. Khanam, A. *et al.* Blockade of Neutrophil's Chemokine Receptors CXCR1/2 Abrogate Liver Damage in Acute-on-Chronic Liver Failure. *Front Immunol* **8**, 464 (2017).
11. Zaldivar, M.M. *et al.* The chemokine receptor CXCR3 limits injury after acute toxic liver damage. *Lab Invest* **92**, 724–34 (2012).
12. Juma, A.R. *et al.* PLAG1 deficiency impairs spermatogenesis and sperm motility in mice. *Sci Rep* **7**, 5317 (2017).
13. Paigen, K. & Petkov, P.M. PRDM9 and Its Role in Genetic Recombination. *Trends Genet* **34**, 291–300 (2018).
14. Pollard, K.S., Hubisz, M.J., Rosenbloom, K.R. & Siepel, A. Detection of nonneutral substitution rates on mammalian phylogenies. *Genome research* **20**, 110–21 (2010).
15. Mehta, T.K. *et al.* Evolution of miRNA-Binding Sites and Regulatory Networks in Cichlids. *Mol Biol Evol* **39**, msac146 (2022).
16. Brawand, D. *et al.* The genomic substrate for adaptive radiation in African cichlid fish. *Nature* **2**, 17–19 (2014).
17. Conte, M.A., Gammerdinger, W.J., Bartie, K.L., Penman, D.J. & Kocher, T.D. A high quality assembly of the Nile Tilapia (Oreochromis niloticus) genome reveals the structure of two sex determination regions. *BMC Genomics* **18**, 341–341 (2017).

18. Conte, M.A. *et al.* Chromosome-scale assemblies reveal the structural evolution of African cichlid genomes. *GigaScience* **8**, giz030–giz030 (2019).
19. Benjamini, Y. & Hochberg, Y. Benjamini Y, Hochberg Y. Controlling the false discovery rate: a practical and powerful approach to multiple testing. *Journal of the Royal Statistical Society B* **57**, 289–300 (1995).
20. Hofmann, H.A. Functional genomics of neural and behavioral plasticity. *J Neurobiol* **54**, 272–82 (2003).
21. Pistocchi, A. *et al.* Crucial role of zebrafish prox1 in hypothalamic catecholaminergic neurons development. *BMC Developmental Biology* **8**, 27 (2008).
22. Acampora, D., Avantaggiato, V., Tuorto, F. & Simeone, A. Genetic control of brain morphogenesis through Otx gene dosage requirement. *Development* **124**, 3639–3650 (1997).
23. He, Z., Agostini, M., Liu, H., Melino, G. & Simon, H.U. p73 regulates basal and starvation-induced liver metabolism in vivo. *Oncotarget* **6**, 33178–90 (2015).
24. Lee, J.M., Lee, D.J., Bae, S.C. & Jung, H.S. Abnormal liver differentiation and excessive angiogenesis in mice lacking Runx3. *Histochem Cell Biol* **139**, 751–8 (2013).
25. Eozenou, C. *et al.* The TALE homeodomain of PBX1 is involved in human primary testis-determination. *Hum Mutat* **40**, 1071–1076 (2019).
26. Ile, K.E. *et al.* Zebrafish class 1 phosphatidylinositol transfer proteins: PITPbeta and double cone cell outer segment integrity in retina. *Traffic* **11**, 1151–67 (2010).
27. Lee, C.S., Friedman, J.R., Fulmer, J.T. & Kaestner, K.H. The initiation of liver development is dependent on Foxa transcription factors. *Nature* **435**, 944–947 (2005).
28. Gusmao, E.G., Allhoff, M., Zenke, M. & Costa, I.G. Analysis of computational footprinting methods for DNase sequencing experiments. *Nat Methods* **13**, 303–9 (2016).
29. Mehta, T.K. *et al.* Chromatin accessibility in gill tissue identifies candidate genes and loci associated with aquaculture relevant traits in tilapia. *Genomics* **115**, 110633 (2023).
30. Carleton, K. Cichlid fish visual systems: mechanisms of spectral tuning. *Integrative zoology* **4**, 75–86 (2009).
31. Hahn, C., Genner, M.J., Turner, G.F. & Joyce, D.A. The genomic basis of cichlid fish adaptation within the deepwater “twilight zone” of Lake Malawi. *Evolution Letters* **1**, 184–198 (2017).
32. Anand, D., Agrawal, S.A., Slavotinek, A. & Lachke, S.A. Mutation update of transcription factor genes FOXE3, HSF4, MAF, and PITX3 causing cataracts and other developmental ocular defects. *Human Mutation* **39**, 471–494 (2018).
33. Snoeks, J., Carvalho, G.R. & Project, S.G.L.M.N.B.C. *The cichlid diversity of Lake Malawi/Nyasa/Niassa : identification, distribution, and taxonomy*, 360 p. : ill. (some col.), maps (some col.) (Cichlid Press, El Paso, Tex, 2004).
34. Malinsky, M. *et al.* Whole-genome sequences of Malawi cichlids reveal multiple radiations interconnected by gene flow. *Nature Ecology & Evolution* **2**, 1940–1955 (2018).
35. Blumer, L.M. *et al.* Introgression dynamics of sex-linked chromosomal inversions shape the Malawi cichlid radiation. *Science* **388**, eadr9961 (2025).

36. Hosseini, R., Balci, A.T., Kostka, D., Clark, N. & Chikina, M. Ultra-fast variant effect prediction using biophysical transcription factor binding models. *bioRxiv*, 2024.06.26.600873 (2025).
37. Felsenstein, J. Phylogenies and the Comparative Method. *The American Naturalist* **125**, 1–15 (1985).

Supplementary Figures

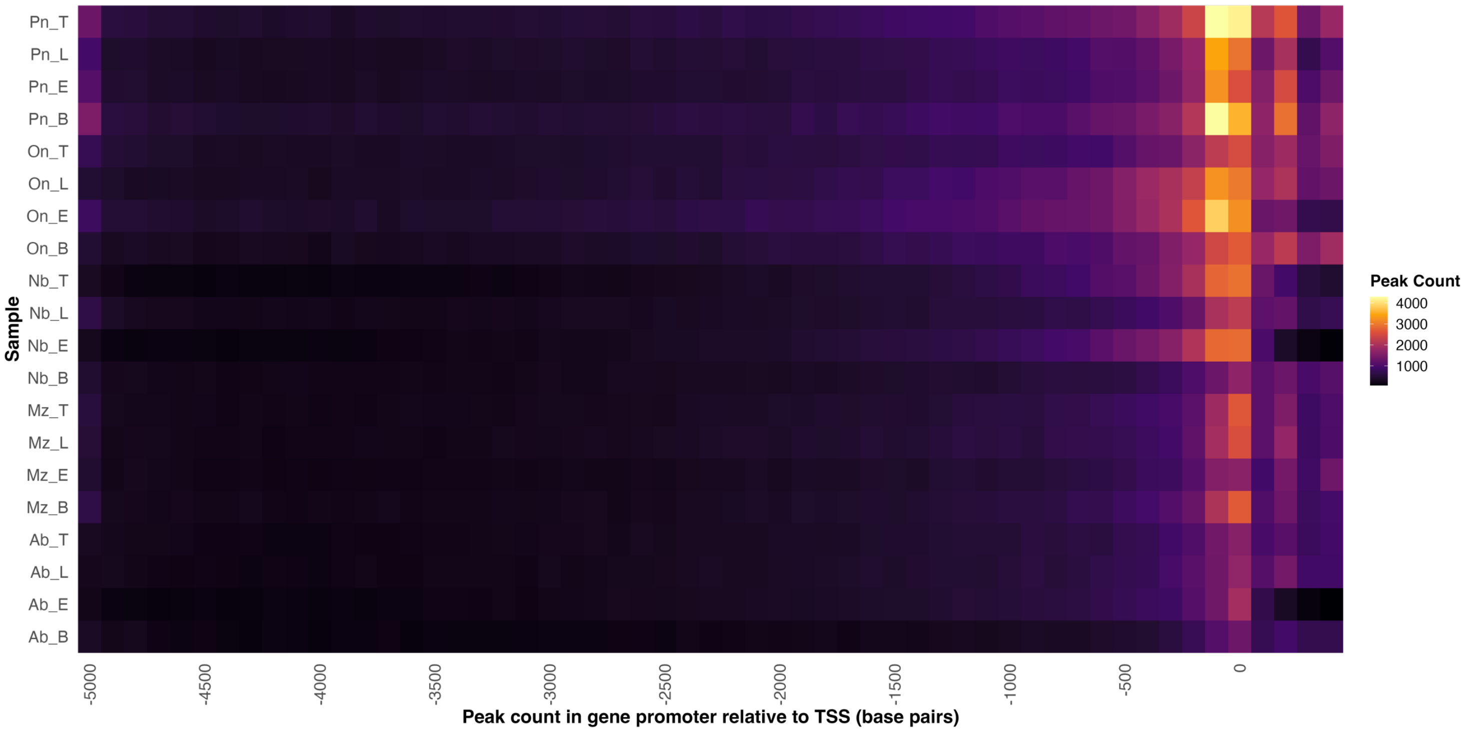

Fig. S1 – Distribution of narrow open-chromatin peaks relative to TSS. Heatmap showing the distribution of peak summits relative to the transcription start site (TSS) for sample replicates. Peak counts were calculated using a 100-bp sliding window from the TSS (at 0 bp) along the 5 kb gene promoter region. The colour scale indicates the number of peaks within each window. Species: Mz = *M. zebra*; Pn = *P. nyererei*; Ab = *A. burtoni*; Nb = *N. brichardi*; On = *O. niloticus*; Tissues: B = Forebrain; E = Retina; L = Liver; T = Testis.

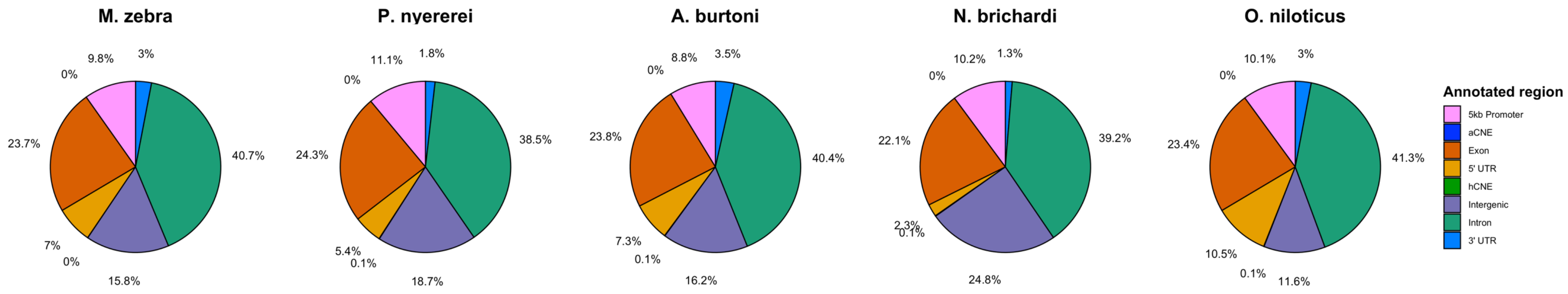

Fig. S2 – Distribution of narrow open-chromatin peaks in annotated features of four tissues in five cichlids. Collated percentage of narrow open-chromatin peaks in all four tissue samples of five cichlids shown outside of each annotated feature with legend of annotations to right.

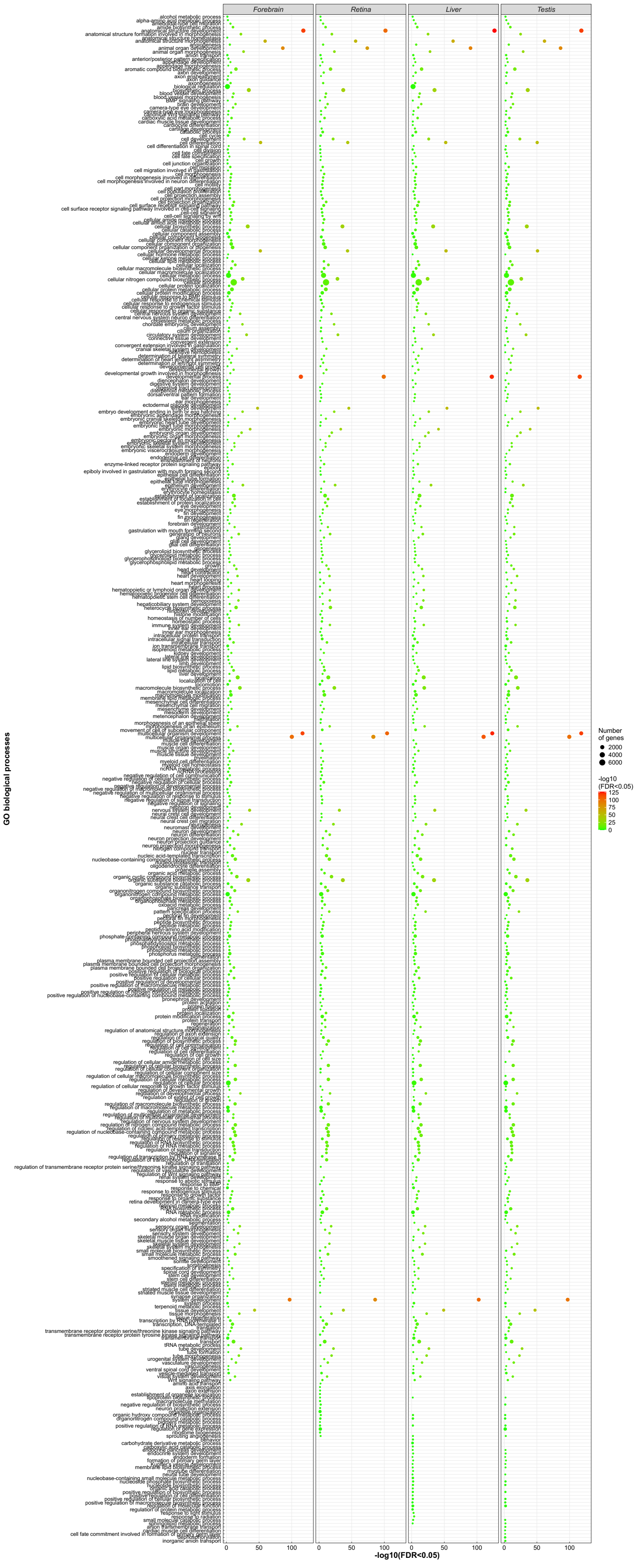

Fig. S3 – Gene Ontology (GO) enrichment of peaks localised to 5 kb gene promoters of 1-to-1 orthologs collated by tissue. GO biological processes enrichment of peaks localised to the up to 5 kb gene promoter regions of 13,889 1-to-1 orthologs collated for all five species by tissue. Circles show enriched biological processes (y-axis) of significance ($-log_{10}$ FDR <0.05, heatmap to right) and $-log_{10}$ FDR <0.05 (x-axis) values of each term across each tissue. Number of enriched genes for each term shown by size of each circle.

(A)

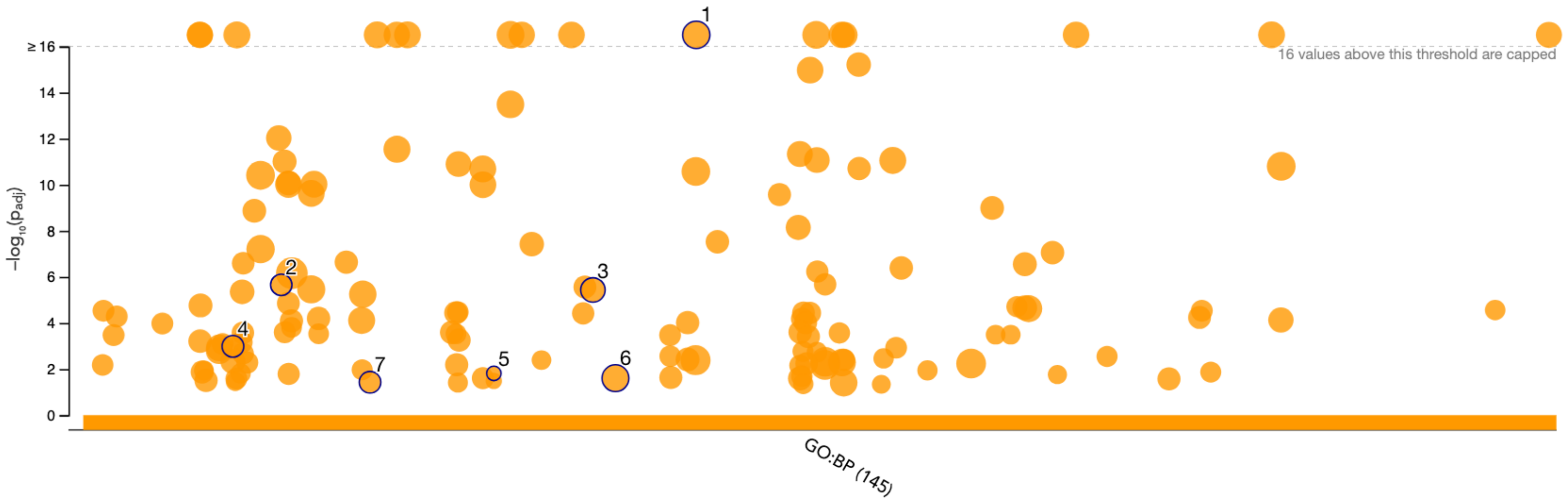

ID	Source	Term ID	Term Name	p_{adj} (query_1)
1	GO:BP	GO:0044271	cellular nitrogen compound biosynthetic process	8.034×10^{-28}
2	GO:BP	GO:0009719	response to endogenous stimulus	2.214×10^{-6}
3	GO:BP	GO:0035556	intracellular signal transduction	3.706×10^{-6}
4	GO:BP	GO:0007167	enzyme-linked receptor protein signaling path...	1.027×10^{-3}
5	GO:BP	GO:0032007	negative regulation of TOR signaling	1.541×10^{-2}
6	GO:BP	GO:0036211	protein modification process	2.482×10^{-2}
7	GO:BP	GO:0016477	cell migration	3.737×10^{-2}

(B)

GO:BP		stats
Term name	Term ID	p_{adj}
developmental process	GO:0032502	2.812×10^{-17}
anatomical structure development	GO:0048856	4.821×10^{-17}
multicellular organism development	GO:0007275	3.826×10^{-16}
system development	GO:0048731	1.410×10^{-13}
cell differentiation	GO:0030154	6.645×10^{-10}
cellular developmental process	GO:0048869	7.070×10^{-10}
multicellular organismal process	GO:0032501	5.077×10^{-9}
anatomical structure morphogenesis	GO:0009653	8.017×10^{-9}
nervous system development	GO:0007399	1.771×10^{-8}
neurogenesis	GO:0022008	6.599×10^{-8}
animal organ development	GO:0048513	1.504×10^{-7}
generation of neurons	GO:0048699	6.864×10^{-7}
tissue development	GO:0009888	1.088×10^{-6}
neuron differentiation	GO:0030182	1.311×10^{-6}
epithelium development	GO:0060429	2.479×10^{-5}
embryo development	GO:0009790	1.067×10^{-4}
cell development	GO:0048468	3.320×10^{-4}
embryonic morphogenesis	GO:0048598	5.015×10^{-4}
central nervous system development	GO:0007417	6.624×10^{-3}
animal organ morphogenesis	GO:0009887	7.899×10^{-3}
sensory system development	GO:0048880	1.122×10^{-2}
regulation of cell differentiation	GO:0045595	1.396×10^{-2}
plasma membrane bounded cell projection morphogene...	GO:0120039	1.500×10^{-2}
neuron projection morphogenesis	GO:0048812	1.500×10^{-2}
cell projection morphogenesis	GO:0048858	1.500×10^{-2}
cell surface receptor protein tyrosine kinase signaling p...	GO:0007169	1.733×10^{-2}
positive regulation of cellular process	GO:0048522	2.126×10^{-2}
regulation of RNA metabolic process	GO:0051252	2.768×10^{-2}
regulation of developmental process	GO:0050793	2.856×10^{-2}
neuron projection development	GO:0031175	2.967×10^{-2}
regulation of nucleobase-containing compound metabo...	GO:0019219	3.233×10^{-2}
regulation of RNA biosynthetic process	GO:2001141	3.874×10^{-2}
regulation of DNA-templated transcription	GO:0006355	3.874×10^{-2}
neuron development	GO:0048666	3.961×10^{-2}
cell morphogenesis	GO:0000902	4.032×10^{-2}

Fig. S4 – Gene Ontology (GO) enrichment of peaks localised to ancestral nodes. GO enrichment of all tissue peaks at **A)** 5536 1-to-1 ortholog gene promoters positionally conserved across all five species (Anc1); and **B)** 1473 1-to-1 ortholog gene promoters positionally conserved across haplochromine species. Circles and bars show enriched biological processes (x-axis and table) of significance (-*log₁₀* FDR <0.05, y-axis) of each term in Supplementary Table S4. In (A) table of displayed driver terms (demarcated with numbers on plot) calculated by the g:GOSt module of g:Profiler (https://biit.cs.ut.ee/gprofiler/gost) based on a two-stage algorithm for filtering GO enrichment results by grouping into sub-ontologies (stage 1) and identifying connected components using a greedy search strategy (stage 2).

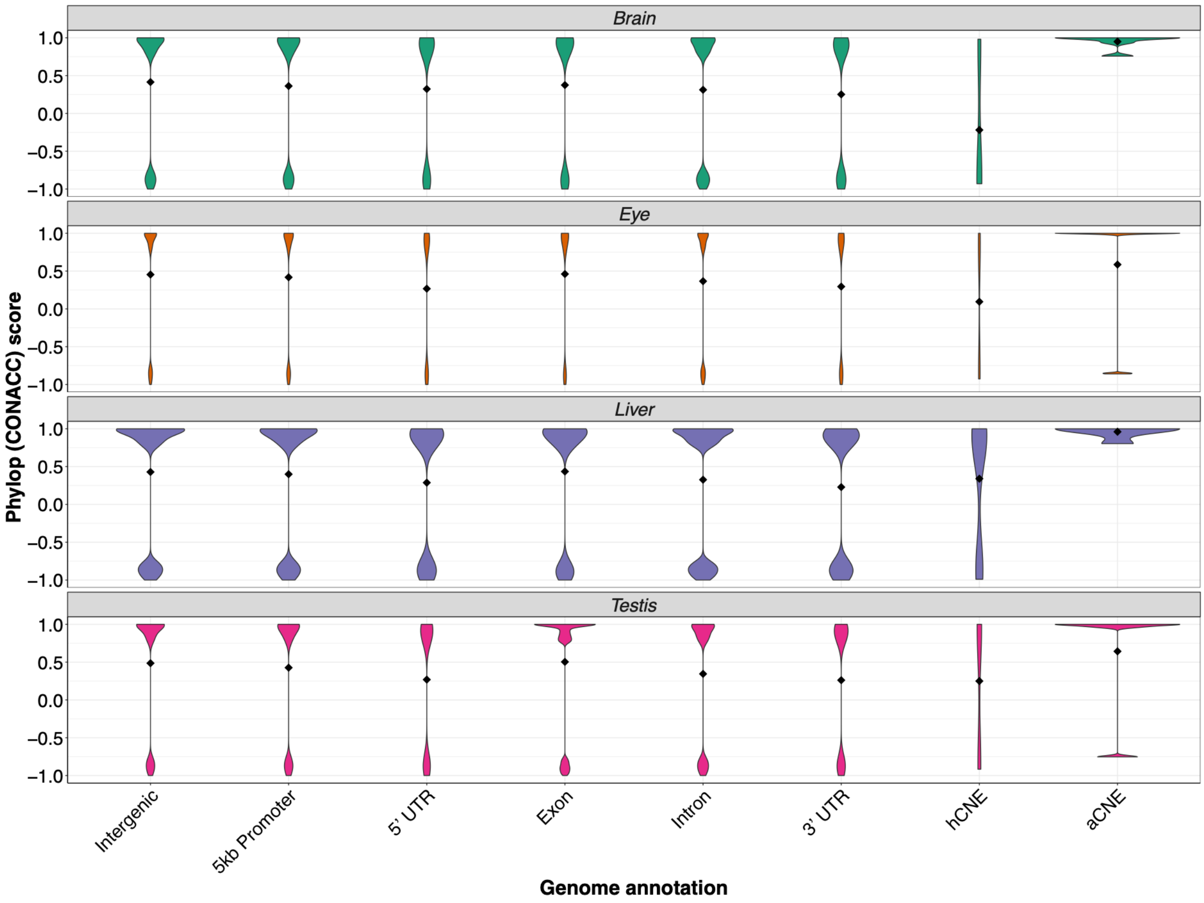

Fig. S5 – PhyloP conservation-acceleration scores of *O. niloticus* open-chromatin peaks collated by tissues and genomic annotations. Violin plot of collated pairwise phyloP conservation-acceleration (CONACC) scores (y-axis) of *O. niloticus* open-chromatin peaks in genomic annotations (x-axis) of four tissues (Brain = Forebrain and Eye = Retina). PhyloP scores were calculated using *O. niloticus* as a reference against all species pairwise comparisons and collated; positive scores indicate pairwise evolutionary conservation, whereas negative scores indicate evolutionary acceleration against a neutral substitution model.

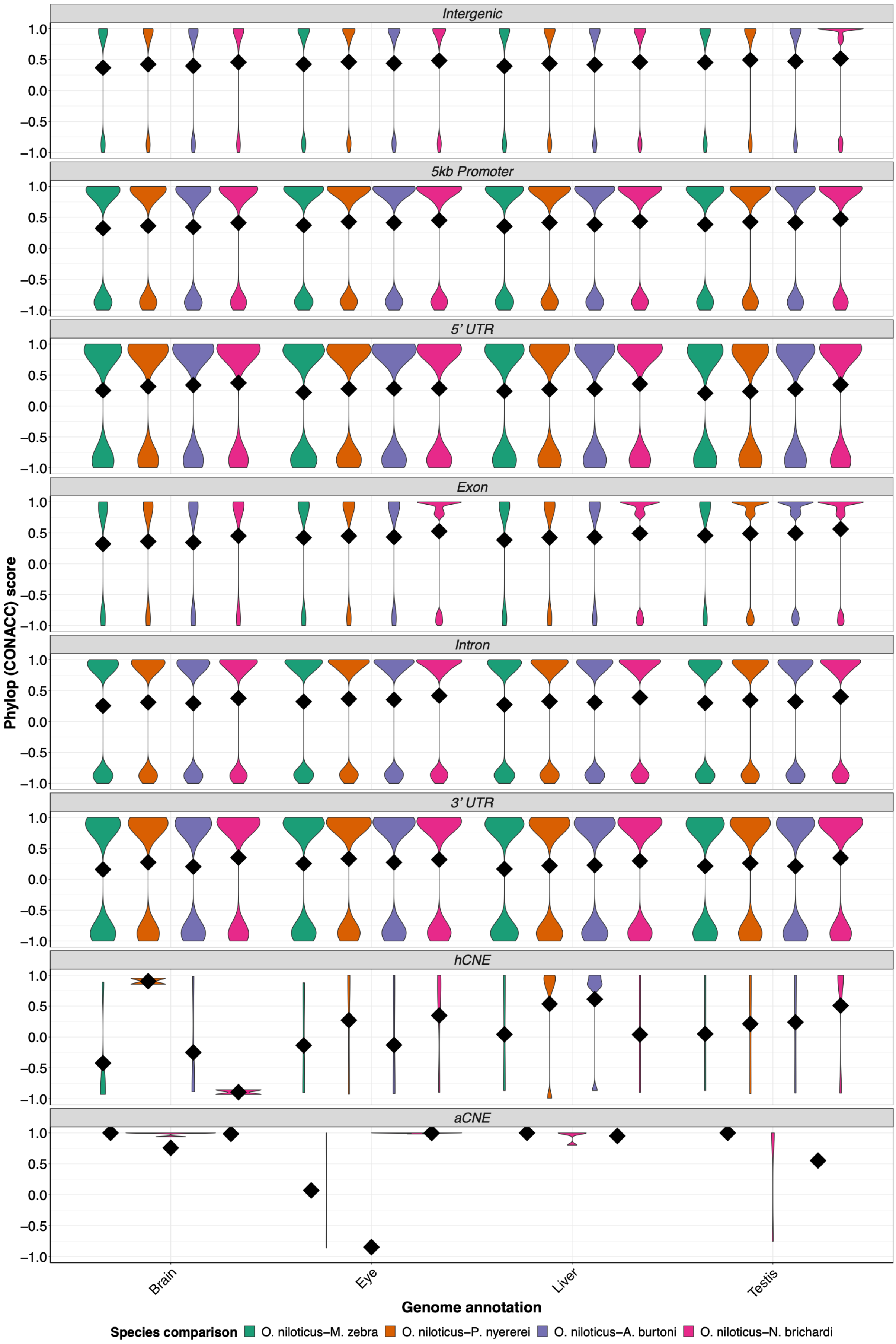
Intergenic
5kb Promoter
5' UTR
Exon
Intron
3' UTR
hCNE
aCNE
1.0
0.5
0.0
−0.5
−1.0
Phylop (CONACC) score
Brain
Eye
Liver
Testis
Genome annotation
Species comparison
O. niloticus−M. zebra
O. niloticus−P. nyererei
O. niloticus−A. burtoni
O. niloticus−N. brichardi

Fig. S6 – PhyloP conservation-acceleration scores of *O. niloticus* open-chromatin peaks in pairwise species comparisons of tissues and genomic annotations. Violin plot of all pairwise phyloP conservation-acceleration (CONACC) scores (y-axis) of *O. niloticus* open-chromatin peaks in genomic annotations of four tissues (x-axis). PhyloP scores were calculated using *O. niloticus* as a reference against all species pairwise comparisons and individually plotted; positive scores indicate pairwise evolutionary conservation, whereas negative scores indicate evolutionary acceleration against a neutral substitution model.

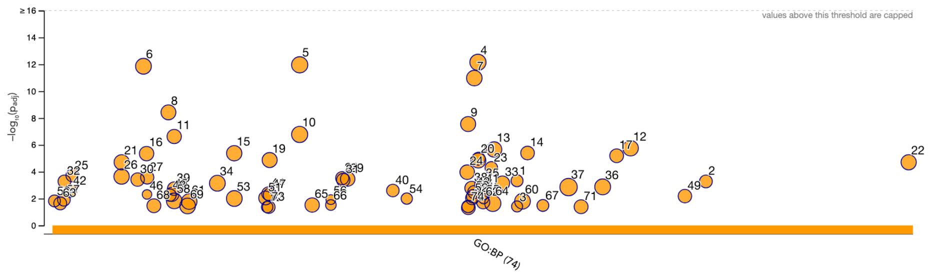

ID	Source	Term ID	Term Name	p_{adj} (query_1)
1	GO:BP	GO:0060041	retina development in camera-type eye	4.703×10^{-4}
2	GO:BP	GO:0150063	visual system development	5.246×10^{-4}
3	GO:BP	GO:0060042	retina morphogenesis in camera-type eye	3.835×10^{-2}
4	GO:BP	GO:0048856	anatomical structure development	7.112×10^{-13}
5	GO:BP	GO:0032502	developmental process	1.093×10^{-12}
6	GO:BP	GO:0007275	multicellular organism development	1.401×10^{-12}
7	GO:BP	GO:0048731	system development	1.065×10^{-11}
8	GO:BP	GO:0009653	anatomical structure morphogenesis	3.800×10^{-9}
9	GO:BP	GO:0048513	animal organ development	2.817×10^{-8}
10	GO:BP	GO:0032501	multicellular organismal process	1.678×10^{-7}
11	GO:BP	GO:0009888	tissue development	2.376×10^{-7}
12	GO:BP	GO:0080090	regulation of primary metabolic process	1.771×10^{-6}
13	GO:BP	GO:0051252	regulation of RNA metabolic process	2.193×10^{-6}
14	GO:BP	GO:0060429	epithelium development	4.052×10^{-6}
15	GO:BP	GO:0019219	regulation of nucleobase-containing compoun...	4.216×10^{-6}
16	GO:BP	GO:0007399	nervous system development	4.479×10^{-6}
17	GO:BP	GO:0072359	circulatory system development	6.457×10^{-6}
18	GO:BP	GO:0048880	sensory system development	1.072×10^{-5}
19	GO:BP	GO:0030154	cell differentiation	1.348×10^{-5}
20	GO:BP	GO:0048869	cellular developmental process	1.462×10^{-5}
21	GO:BP	GO:0006355	regulation of DNA-templated transcription	1.977×10^{-5}
22	GO:BP	GO:2001141	regulation of RNA biosynthetic process	1.977×10^{-5}
23	GO:BP	GO:0051128	regulation of cellular component organization	5.196×10^{-5}
24	GO:BP	GO:0048468	cell development	1.083×10^{-4}
25	GO:BP	GO:0002009	morphogenesis of an epithelium	1.893×10^{-4}
26	GO:BP	GO:0006351	DNA-templated transcription	2.271×10^{-4}
27	GO:BP	GO:0007423	sensory organ development	2.650×10^{-4}
28	GO:BP	GO:0035239	tube morphogenesis	2.979×10^{-4}
29	GO:BP	GO:0035556	intracellular signal transduction	3.478×10^{-4}
30	GO:BP	GO:0007010	cytoskeleton organization	3.843×10^{-4}
31	GO:BP	GO:0035295	tube development	4.142×10^{-4}
32	GO:BP	GO:0001654	eye development	5.246×10^{-4}
33	GO:BP	GO:0051641	cellular localization	7.085×10^{-4}
34	GO:BP	GO:0016043	cellular component organization	7.189×10^{-4}
35	GO:BP	GO:0050794	regulation of cellular process	1.042×10^{-3}
36	GO:BP	GO:0071840	cellular component organization or biogenesis	1.341×10^{-3}
37	GO:BP	GO:0065007	biological regulation	1.390×10^{-3}
38	GO:BP	GO:0048646	anatomical structure formation involved in mor...	1.664×10^{-3}
39	GO:BP	GO:0009887	animal organ morphogenesis	1.814×10^{-3}
40	GO:BP	GO:0043010	camera-type eye development	2.515×10^{-3}
41	GO:BP	GO:0050789	regulation of biological process	2.746×10^{-3}
42	GO:BP	GO:0001944	vasculature development	2.765×10^{-3}
43	GO:BP	GO:0048729	tissue morphogenesis	3.295×10^{-3}
44	GO:BP	GO:0030030	cell projection organization	4.256×10^{-3}
45	GO:BP	GO:0009790	embryo development	4.862×10^{-3}
46	GO:BP	GO:0007416	synapse assembly	4.913×10^{-3}
47	GO:BP	GO:0030182	neuron differentiation	5.245×10^{-3}
48	GO:BP	GO:0009719	response to endogenous stimulus	5.497×10^{-3}
49	GO:BP	GO:0120036	plasma membrane bounded cell projection org...	6.547×10^{-3}
50	GO:BP	GO:0048699	generation of neurons	6.740×10^{-3}
51	GO:BP	GO:0022008	neurogenesis	9.003×10^{-3}
52	GO:BP	GO:0048666	neuron development	9.270×10^{-3}
53	GO:BP	GO:0019222	regulation of metabolic process	9.861×10^{-3}
54	GO:BP	GO:0044087	regulation of cellular component biogenesis	1.001×10^{-2}
55	GO:BP	GO:0050808	synapse organization	1.023×10^{-2}
56	GO:BP	GO:0034329	cell junction assembly	1.202×10^{-2}
57	GO:BP	GO:0001568	blood vessel development	1.228×10^{-2}
58	GO:BP	GO:0009889	regulation of biosynthetic process	1.494×10^{-2}
59	GO:BP	GO:0000226	microtubule cytoskeleton organization	1.498×10^{-2}
60	GO:BP	GO:0060255	regulation of macromolecule metabolic process	1.570×10^{-2}
61	GO:BP	GO:0010556	regulation of macromolecule biosynthetic proc...	1.666×10^{-2}
62	GO:BP	GO:0050793	regulation of developmental process	1.872×10^{-2}
63	GO:BP	GO:0000902	cell morphogenesis	2.279×10^{-2}
64	GO:BP	GO:0051179	localization	2.285×10^{-2}
65	GO:BP	GO:0033036	macromolecule localization	3.007×10^{-2}
66	GO:BP	GO:0034330	cell junction organization	3.016×10^{-2}
67	GO:BP	GO:0060972	left/right pattern formation	3.250×10^{-2}
68	GO:BP	GO:0008104	protein localization	3.393×10^{-2}
69	GO:BP	GO:0010468	regulation of gene expression	3.550×10^{-2}
70	GO:BP	GO:0048514	blood vessel morphogenesis	3.850×10^{-2}
71	GO:BP	GO:0070727	cellular macromolecule localization	4.030×10^{-2}
72	GO:BP	GO:0022603	regulation of anatomical structure morphogene...	4.246×10^{-2}
73	GO:BP	GO:0030036	actin cytoskeleton organization	4.338×10^{-2}
74	GO:BP	GO:0048522	positive regulation of cellular process	4.792×10^{-2}

Fig. S7 – Gene Ontology (GO) enrichment of positionally conserved and accelerated peaks localised to 3061 1-to-1 orthologous gene promoters along the five species phylogeny. Circles show enriched biological processes (x-axis and table) of significance (-log_{10} FDR <0.05, y-axis) of each Nile tilapia term. Table of terms (demarcated with numbers on plot) calculated by the g:GOSt module of g:Profiler (https://biit.cs.ut.ee/gprofiler/gost).

(A) *M. zebra* Forebrain

GO:BP		stats	
Term name	Term ID	p_{adj}	$-\log_{10}(p_{adj})$ (0 – ≤16)
developmental process	GO:0032502	7.121×10^{-55}	
anatomical structure development	GO:0048856	4.322×10^{-53}	
multicellular organism development	GO:0007275	8.851×10^{-49}	
system development	GO:0048731	6.015×10^{-40}	
multicellular organismal process	GO:0032501	4.352×10^{-31}	
cellular developmental process	GO:0048869	5.781×10^{-30}	
cell differentiation	GO:0030154	9.826×10^{-30}	
animal organ development	GO:0048513	1.515×10^{-27}	
anatomical structure morphogenesis	GO:0009653	3.855×10^{-26}	
nervous system development	GO:0007399	1.000×10^{-24}	
tissue development	GO:0009888	2.031×10^{-20}	
transport	GO:0006810	4.073×10^{-18}	
neurogenesis	GO:0022008	1.968×10^{-16}	
localization	GO:0051179	4.528×10^{-16}	
generation of neurons	GO:0048699	6.116×10^{-16}	
establishment of localization	GO:0051234	6.299×10^{-16}	
regulation of cellular process	GO:0050794	8.868×10^{-16}	
biological regulation	GO:0065007	1.606×10^{-15}	
regulation of biological process	GO:0050789	3.912×10^{-15}	
embryo development	GO:0009790	2.899×10^{-14}	
neuron differentiation	GO:0030182	5.190×10^{-14}	
transmembrane transport	GO:0055085	1.171×10^{-13}	
central nervous system development	GO:0007417	3.961×10^{-13}	
cell development	GO:0048468	2.238×10^{-12}	
epithelium development	GO:0060429	5.389×10^{-12}	
regulation of primary metabolic process	GO:0080090	1.201×10^{-11}	
DNA-templated transcription	GO:0006351	1.387×10^{-11}	
regulation of RNA biosynthetic process	GO:2001141	1.782×10^{-11}	
regulation of DNA-templated transcription	GO:0006355	1.782×10^{-11}	
regulation of RNA metabolic process	GO:0051252	2.678×10^{-11}	
regulation of nucleobase-containing compound metabo...	GO:0019219	4.064×10^{-11}	
anatomical structure formation involved in morphogene...	GO:0048646	5.329×10^{-10}	
regulation of developmental process	GO:0050793	5.503×10^{-10}	
embryonic morphogenesis	GO:0048598	2.075×10^{-9}	
animal organ morphogenesis	GO:0009887	1.582×10^{-8}	
monoatomic ion transmembrane transport	GO:0034220	1.681×10^{-8}	
monoatomic ion transport	GO:0006811	1.921×10^{-8}	
sensory organ development	GO:0007423	2.333×10^{-8}	
sensory system development	GO:0048880	2.884×10^{-8}	
cell communication	GO:0007154	8.026×10^{-8}	
signaling	GO:0023052	2.253×10^{-7}	
gland development	GO:0048732	2.751×10^{-7}	
tube development	GO:0035295	3.638×10^{-7}	
enzyme-linked receptor protein signaling pathway	GO:0007167	3.993×10^{-7}	
cellular process	GO:0009987	6.732×10^{-7}	
cell fate commitment	GO:0045165	9.771×10^{-7}	
neuron development	GO:0048666	9.879×10^{-7}	
cell morphogenesis	GO:0000902	1.626×10^{-6}	
pattern specification process	GO:0007389	1.647×10^{-6}	
axon development	GO:0061564	1.734×10^{-6}	
regulation of cell communication	GO:0010646	2.364×10^{-6}	
regulation of signaling	GO:0023051	2.451×10^{-6}	
signal transduction	GO:0007165	3.887×10^{-6}	
cell projection morphogenesis	GO:0048858	4.090×10^{-6}	
neuron projection morphogenesis	GO:0048812	4.090×10^{-6}	
plasma membrane bounded cell projection morphogene...	GO:0120039	4.090×10^{-6}	
inorganic ion transmembrane transport	GO:0098660	4.263×10^{-6}	
head development	GO:0060322	5.364×10^{-6}	
tissue morphogenesis	GO:0048729	5.409×10^{-6}	
brain development	GO:0007420	7.106×10^{-6}	
cell morphogenesis involved in neuron differentiation	GO:0048667	7.266×10^{-6}	
regionalization	GO:0003002	9.512×10^{-6}	
axonogenesis	GO:0007409	9.729×10^{-6}	
chordate embryonic development	GO:0043009	1.244×10^{-5}	
embryo development ending in birth or egg hatching	GO:0009792	1.244×10^{-5}	
regulation of signal transduction	GO:0009966	1.294×10^{-5}	
circulatory system development	GO:0072359	2.165×10^{-5}	
cell surface receptor protein tyrosine kinase signaling p...	GO:0007169	2.257×10^{-5}	
response to endogenous stimulus	GO:0009719	3.547×10^{-5}	
gastrulation	GO:0007369	4.102×10^{-5}	
neuron projection guidance	GO:0097485	4.206×10^{-5}	
axon guidance	GO:0007411	4.206×10^{-5}	
vasculature development	GO:0001944	4.943×10^{-5}	
cellular response to endogenous stimulus	GO:0071495	4.943×10^{-5}	
metal ion transport	GO:0030001	5.691×10^{-5}	
neuron projection development	GO:0031175	5.953×10^{-5}	
hepaticobiliary system development	GO:0061008	6.270×10^{-5}	
liver development	GO:0001889	8.259×10^{-5}	
cell fate specification	GO:0001708	9.528×10^{-5}	
morphogenesis of an epithelium	GO:0002009	9.591×10^{-5}	
eye development	GO:0001654	1.526×10^{-4}	
visual system development	GO:0150063	1.526×10^{-4}	
plasma membrane bounded cell projection organization	GO:0120036	1.546×10^{-4}	
regulation of cell differentiation	GO:0045595	1.688×10^{-4}	
tube morphogenesis	GO:0035239	2.168×10^{-4}	
blood vessel development	GO:0001568	2.932×10^{-4}	
negative regulation of developmental process	GO:0051093	3.138×10^{-4}	
regulation of transcription by RNA polymerase II	GO:0006357	3.159×10^{-4}	
regulation of biological quality	GO:0065008	3.722×10^{-4}	
regulation of multicellular organismal development	GO:2000026	4.047×10^{-4}	
transcription by RNA polymerase II	GO:0006366	4.728×10^{-4}	
sensory organ morphogenesis	GO:0090596	4.939×10^{-4}	
positive regulation of cellular process	GO:0048522	5.625×10^{-4}	
somitogenesis	GO:0001756	6.691×10^{-4}	
cell projection organization	GO:0030030	8.504×10^{-4}	
monoatomic anion transport	GO:0006820	8.953×10^{-4}	
synaptic signaling	GO:0099536	9.334×10^{-4}	
segmentation	GO:0035282	1.052×10^{-3}	
inorganic cation transmembrane transport	GO:0098662	1.284×10^{-3}	
cell-cell signaling	GO:0007267	1.583×10^{-3}	

(B) *P. nyererei* Forebrain

GO:BP		stats	
Term name	Term ID	p_{adj}	$-\log_{10}(p_{adj})$ (0 – ≤16)
anatomical structure development	GO:0048856	6.677×10^{-43}	
developmental process	GO:0032502	6.287×10^{-42}	
multicellular organism development	GO:0007275	5.180×10^{-39}	
system development	GO:0048731	1.020×10^{-32}	
animal organ development	GO:0048513	3.289×10^{-22}	
anatomical structure morphogenesis	GO:0009653	8.222×10^{-22}	
cellular developmental process	GO:0048869	8.162×10^{-21}	
cell differentiation	GO:0030154	1.339×10^{-20}	
multicellular organismal process	GO:0032501	1.221×10^{-18}	
nervous system development	GO:0007399	2.468×10^{-16}	
transport	GO:0006810	1.904×10^{-14}	
tissue development	GO:0009888	4.050×10^{-14}	
neuron differentiation	GO:0030182	7.455×10^{-14}	
generation of neurons	GO:0048699	1.112×10^{-13}	
localization	GO:0051179	7.386×10^{-13}	
cell development	GO:0048468	1.119×10^{-12}	
establishment of localization	GO:0051234	2.294×10^{-12}	
neurogenesis	GO:0022008	4.383×10^{-12}	
epithelium development	GO:0060429	8.531×10^{-12}	
tube development	GO:0035295	2.112×10^{-10}	
embryo development	GO:0009790	1.579×10^{-9}	
neuron development	GO:0048666	3.015×10^{-9}	
anatomical structure formation involved in morphogene...	GO:0048646	3.841×10^{-9}	
cell morphogenesis	GO:0000902	6.270×10^{-9}	
Wnt signaling pathway	GO:0016055	8.283×10^{-9}	
cell morphogenesis involved in neuron differentiation	GO:0048667	1.108×10^{-8}	
biological regulation	GO:0065007	1.109×10^{-8}	
axon development	GO:0061564	1.410×10^{-8}	
neuron projection development	GO:0031175	1.866×10^{-8}	
regulation of developmental process	GO:0050793	2.469×10^{-8}	
regulation of cellular process	GO:0050794	2.492×10^{-8}	
neuron projection morphogenesis	GO:0048812	3.483×10^{-8}	
plasma membrane bounded cell projection morphogene...	GO:0120039	3.483×10^{-8}	
cell projection morphogenesis	GO:0048858	3.483×10^{-8}	
axonogenesis	GO:0007409	5.017×10^{-8}	
circulatory system development	GO:0072359	7.662×10^{-8}	
regulation of biological process	GO:0050789	1.510×10^{-7}	
cellular response to endogenous stimulus	GO:0071495	2.735×10^{-7}	
response to endogenous stimulus	GO:0009719	3.692×10^{-7}	
regulation of primary metabolic process	GO:0080090	8.037×10^{-7}	
sensory organ development	GO:0007423	1.419×10^{-6}	
phosphate-containing compound metabolic process	GO:0006796	2.794×10^{-6}	
phosphorus metabolic process	GO:0006793	2.794×10^{-6}	
tube morphogenesis	GO:0035239	3.115×10^{-6}	
regulation of RNA metabolic process	GO:0051252	6.061×10^{-6}	
transmembrane transport	GO:0055085	9.876×10^{-6}	
regulation of signaling	GO:0023051	9.966×10^{-6}	
regulation of signal transduction	GO:0009966	1.069×10^{-5}	
regulation of cell communication	GO:0010646	1.096×10^{-5}	
sensory system development	GO:0048880	1.281×10^{-5}	
central nervous system development	GO:0007417	1.480×10^{-5}	
cellular process	GO:0009987	1.861×10^{-5}	
regulation of nucleobase-containing compound metabo...	GO:0019219	1.929×10^{-5}	
enzyme-linked receptor protein signaling pathway	GO:0007167	2.882×10^{-5}	
vasculature development	GO:0001944	5.264×10^{-5}	
head development	GO:0060322	5.944×10^{-5}	
plasma membrane bounded cell projection organization	GO:0120036	6.066×10^{-5}	
embryonic morphogenesis	GO:0048598	6.113×10^{-5}	
neuron projection guidance	GO:0097485	8.859×10^{-5}	
axon guidance	GO:0007411	8.859×10^{-5}	
cell communication	GO:0007154	9.446×10^{-5}	
embryo development ending in birth or egg hatching	GO:0009792	9.675×10^{-5}	
chordate embryonic development	GO:0043009	9.675×10^{-5}	
blood vessel morphogenesis	GO:0048514	1.181×10^{-4}	
regulation of DNA-templated transcription	GO:0006355	1.410×10^{-4}	
regulation of RNA biosynthetic process	GO:2001141	1.410×10^{-4}	
DNA-templated transcription	GO:0006351	1.619×10^{-4}	
brain development	GO:0007420	2.156×10^{-4}	
intracellular signaling cassette	GO:0141124	2.227×10^{-4}	
angiogenesis	GO:0001525	2.333×10^{-4}	
muscle structure development	GO:0061061	3.040×10^{-4}	
cell projection organization	GO:0030030	3.291×10^{-4}	
blood vessel development	GO:0001568	3.736×10^{-4}	
regulation of multicellular organismal development	GO:2000026	5.990×10^{-4}	
vesicle-mediated transport	GO:0016192	1.121×10^{-3}	
response to growth factor	GO:0070848	1.216×10^{-3}	
cellular response to growth factor stimulus	GO:0071363	1.216×10^{-3}	
signaling	GO:0023052	1.230×10^{-3}	
monoatomic ion transport	GO:0006811	1.324×10^{-3}	
negative regulation of signaling	GO:0023057	1.448×10^{-3}	
negative regulation of cell communication	GO:0010648	1.448×10^{-3}	
signal transduction	GO:0007165	1.859×10^{-3}	
negative regulation of signal transduction	GO:0009968	2.230×10^{-3}	
morphogenesis of an epithelium	GO:0002009	2.550×10^{-3}	
positive regulation of cellular process	GO:0048522	3.528×10^{-3}	
regulation of Wnt signaling pathway	GO:0030111	3.612×10^{-3}	
epidermis development	GO:0008544	4.006×10^{-3}	
monoatomic ion transmembrane transport	GO:0034220	4.150×10^{-3}	
epithelial cell differentiation	GO:0030855	5.087×10^{-3}	
regulation of cell differentiation	GO:0045595	6.218×10^{-3}	
regulation of biological quality	GO:0065008	9.811×10^{-3}	
negative regulation of response to stimulus	GO:0048585	1.048×10^{-2}	
canonical Wnt signaling pathway	GO:0060070	1.150×10^{-2}	
protein phosphorylation	GO:0006468	1.495×10^{-2}	
organophosphate metabolic process	GO:0019637	1.835×10^{-2}	
phosphorylation	GO:0016310	1.847×10^{-2}	
eye development	GO:0001654	1.882×10^{-2}	
visual system development	GO:0150063	1.882×10^{-2}	
heart development	GO:0007507	2.142×10^{-2}	
ameboidal-type cell migration	GO:0001667	2.231×10^{-2}	

(C) *A. burtoni* Forebrain

GO:BP		stats	
Term name	Term ID	p_{adj}	$-\log_{10}(p_{adj})$ (0 … ≤16)
system development	GO:0048731	2.981×10^{-14}	
multicellular organism development	GO:0007275	9.199×10^{-13}	
anatomical structure development	GO:0048856	3.857×10^{-10}	
nervous system development	GO:0007399	4.212×10^{-10}	
developmental process	GO:0032502	1.123×10^{-9}	
cell differentiation	GO:0030154	4.099×10^{-9}	
cellular developmental process	GO:0048869	4.403×10^{-9}	
neurogenesis	GO:0022008	5.667×10^{-7}	
generation of neurons	GO:0048699	3.584×10^{-6}	
neuron differentiation	GO:0030182	4.243×10^{-6}	
transport	GO:0006810	5.412×10^{-6}	
localization	GO:0051179	6.258×10^{-6}	
establishment of localization	GO:0051234	1.285×10^{-5}	
cellular process	GO:0009987	2.558×10^{-5}	
monoatomic ion transmembrane transport	GO:0034220	3.056×10^{-5}	
multicellular organismal process	GO:0032501	3.879×10^{-5}	
transmembrane transport	GO:0055085	1.789×10^{-4}	
cell development	GO:0048468	4.402×10^{-4}	
response to hormone	GO:0009725	4.506×10^{-4}	
inorganic ion transmembrane transport	GO:0098660	6.183×10^{-4}	
response to endogenous stimulus	GO:0009719	6.717×10^{-4}	
cellular response to hormone stimulus	GO:0032870	7.278×10^{-4}	
cellular response to endogenous stimulus	GO:0071495	1.206×10^{-3}	
animal organ development	GO:0048513	2.331×10^{-3}	
enzyme-linked receptor protein signaling pathway	GO:0007167	2.350×10^{-3}	
homeostasis of number of cells	GO:0048872	4.358×10^{-3}	
regulation of cell communication	GO:0010646	4.489×10^{-3}	
monoatomic anion transmembrane transport	GO:0098656	5.269×10^{-3}	
regulation of signaling	GO:0023051	6.950×10^{-3}	
monoatomic ion transport	GO:0006811	8.812×10^{-3}	
myeloid cell differentiation	GO:0030099	9.018×10^{-3}	
cell surface receptor protein tyrosine kinase signaling p...	GO:0007169	1.276×10^{-2}	
monoatomic anion transport	GO:0006820	1.364×10^{-2}	
myeloid cell homeostasis	GO:0002262	1.586×10^{-2}	
positive regulation of cellular process	GO:0048522	1.596×10^{-2}	
sensory system development	GO:0048880	1.974×10^{-2}	
hemopoiesis	GO:0030097	2.055×10^{-2}	
regulation of signal transduction	GO:0009966	2.230×10^{-2}	
erythrocyte differentiation	GO:0030218	2.696×10^{-2}	
erythrocyte homeostasis	GO:0034101	2.696×10^{-2}	
eye development	GO:0001654	3.512×10^{-2}	
visual system development	GO:0150063	3.512×10^{-2}	
inorganic anion transport	GO:0015698	4.303×10^{-2}	
vasculature development	GO:0001944	4.650×10^{-2}	
hypothalamus development	GO:0021854	4.923×10^{-2}	
tube morphogenesis	GO:0035239	4.940×10^{-2}	

(D) *N. brichardi* Forebrain

GO:BP		stats	
Term name	Term ID	p_{adj}	$-\log_{10}(p_{adj})$ (0 … ≤16)
anatomical structure development	GO:0048856	2.646×10^{-38}	
developmental process	GO:0032502	9.440×10^{-37}	
multicellular organism development	GO:0007275	1.949×10^{-31}	
animal organ development	GO:0048513	1.429×10^{-24}	
system development	GO:0048731	5.869×10^{-24}	
cell differentiation	GO:0030154	1.333×10^{-18}	
tissue development	GO:0009888	1.545×10^{-18}	
cellular developmental process	GO:0048869	1.569×10^{-18}	
anatomical structure morphogenesis	GO:0009653	2.042×10^{-18}	
multicellular organismal process	GO:0032501	1.926×10^{-16}	
epithelium development	GO:0060429	6.885×10^{-14}	
nervous system development	GO:0007399	1.213×10^{-12}	
embryo development	GO:0009790	1.474×10^{-12}	
localization	GO:0051179	7.621×10^{-12}	
transport	GO:0006810	2.117×10^{-11}	
generation of neurons	GO:0048699	1.106×10^{-10}	
neurogenesis	GO:0022008	1.371×10^{-10}	
cell development	GO:0048468	1.767×10^{-10}	
establishment of localization	GO:0051234	2.324×10^{-10}	
neuron differentiation	GO:0030182	5.781×10^{-10}	
central nervous system development	GO:0007417	3.672×10^{-9}	
cellular process	GO:0009987	1.823×10^{-8}	
Wnt signaling pathway	GO:0016055	2.561×10^{-8}	
sensory organ development	GO:0007423	6.016×10^{-8}	
tissue morphogenesis	GO:0048729	8.467×10^{-8}	
regulation of cell communication	GO:0010646	1.353×10^{-7}	
anatomical structure formation involved in morphogene...	GO:0048646	1.574×10^{-7}	
sensory system development	GO:0048880	1.827×10^{-7}	
regulation of signaling	GO:0023051	2.053×10^{-7}	
regulation of primary metabolic process	GO:0080090	3.158×10^{-7}	
regulation of signal transduction	GO:0009966	4.110×10^{-7}	
regulation of RNA metabolic process	GO:0051252	1.351×10^{-6}	
embryonic morphogenesis	GO:0048598	1.955×10^{-6}	
biological regulation	GO:0065007	2.038×10^{-6}	
embryo development ending in birth or egg hatching	GO:0009792	4.084×10^{-6}	
chordate embryonic development	GO:0043009	4.084×10^{-6}	
regulation of nucleobase-containing compound metabo...	GO:0019219	5.128×10^{-6}	
animal organ morphogenesis	GO:0009887	6.078×10^{-6}	
tube development	GO:0035295	6.893×10^{-6}	
regulation of cellular process	GO:0050794	7.239×10^{-6}	
regulation of biological process	GO:0050789	8.928×10^{-6}	
morphogenesis of an epithelium	GO:0002009	1.141×10^{-5}	
regulation of RNA biosynthetic process	GO:2001141	2.018×10^{-5}	
regulation of DNA-templated transcription	GO:0006355	2.018×10^{-5}	
vesicle-mediated transport	GO:0016192	3.017×10^{-5}	
circulatory system development	GO:0072359	4.575×10^{-5}	
neuron development	GO:0048666	4.703×10^{-5}	
camera-type eye development	GO:0043010	5.676×10^{-5}	
DNA-templated transcription	GO:0006351	7.597×10^{-5}	
visual system development	GO:0150063	1.055×10^{-4}	
eye development	GO:0001654	1.055×10^{-4}	
head development	GO:0060322	1.733×10^{-4}	
axon development	GO:0061564	1.902×10^{-4}	
cellular localization	GO:0051641	2.928×10^{-4}	
establishment of localization in cell	GO:0051649	4.520×10^{-4}	
embryonic organ development	GO:0048568	4.781×10^{-4}	
plasma membrane bounded cell projection organization	GO:0120036	4.972×10^{-4}	
regulation of developmental process	GO:0050793	5.760×10^{-4}	
brain development	GO:0007420	6.930×10^{-4}	
cell fate commitment	GO:0045165	9.559×10^{-4}	
regionalization	GO:0003002	1.016×10^{-3}	
retina development in camera-type eye	GO:0060041	1.024×10^{-3}	
phosphate-containing compound metabolic process	GO:0006796	1.102×10^{-3}	
phosphorus metabolic process	GO:0006793	1.102×10^{-3}	
macromolecule localization	GO:0033036	1.431×10^{-3}	
cell projection morphogenesis	GO:0048858	1.620×10^{-3}	
plasma membrane bounded cell projection morphogene...	GO:0120039	1.620×10^{-3}	
neuron projection morphogenesis	GO:0048812	1.620×10^{-3}	
axonogenesis	GO:0007409	1.779×10^{-3}	
protein transport	GO:0015031	2.081×10^{-3}	
negative regulation of signal transduction	GO:0009968	2.220×10^{-3}	
pattern specification process	GO:0007389	2.494×10^{-3}	
cell morphogenesis	GO:0000902	2.530×10^{-3}	
negative regulation of cell communication	GO:0010648	2.743×10^{-3}	
negative regulation of signaling	GO:0023057	2.743×10^{-3}	
tube morphogenesis	GO:0035239	2.839×10^{-3}	
negative regulation of response to stimulus	GO:0048585	3.027×10^{-3}	
nitrogen compound transport	GO:0071705	3.136×10^{-3}	
positive regulation of cellular process	GO:0048522	3.178×10^{-3}	
cell morphogenesis involved in neuron differentiation	GO:0048667	3.914×10^{-3}	
regulation of multicellular organismal development	GO:2000026	4.855×10^{-3}	
neuron projection development	GO:0031175	4.920×10^{-3}	
regulation of transcription by RNA polymerase II	GO:0006357	5.337×10^{-3}	
growth	GO:0040007	5.428×10^{-3}	
cell-cell signaling	GO:0007267	5.917×10^{-3}	
developmental growth	GO:0048589	6.081×10^{-3}	
cell communication	GO:0007154	6.843×10^{-3}	
cell projection organization	GO:0030030	6.885×10^{-3}	
enzyme-linked receptor protein signaling pathway	GO:0007167	6.921×10^{-3}	
chemical synaptic transmission	GO:0007268	7.063×10^{-3}	
anterograde trans-synaptic signaling	GO:0098916	7.063×10^{-3}	
trans-synaptic signaling	GO:0099537	7.063×10^{-3}	
central nervous system neuron differentiation	GO:0021953	7.342×10^{-3}	
cell fate specification	GO:0001708	7.630×10^{-3}	
synaptic signaling	GO:0099536	7.685×10^{-3}	
transcription by RNA polymerase II	GO:0006366	7.819×10^{-3}	
sensory organ morphogenesis	GO:0090596	8.199×10^{-3}	
cellular response to endogenous stimulus	GO:0071495	9.549×10^{-3}	
protein modification process	GO:0036211	1.014×10^{-2}	
canonical Wnt signaling pathway	GO:0060070	1.058×10^{-2}	

Fig. S8 – Gene Ontology (GO) enrichment of gene promoter peak gains versus *O. niloticus* peaks in forebrain tissue of four species. Bar plot (with table) of significance (-log_{10} FDR <0.05, y-axis) of each Nile tilapia term shown for each species. Table of terms calculated by the g:GOSt module of g:Profiler (https://biit.cs.ut.ee/gprofiler/gost).

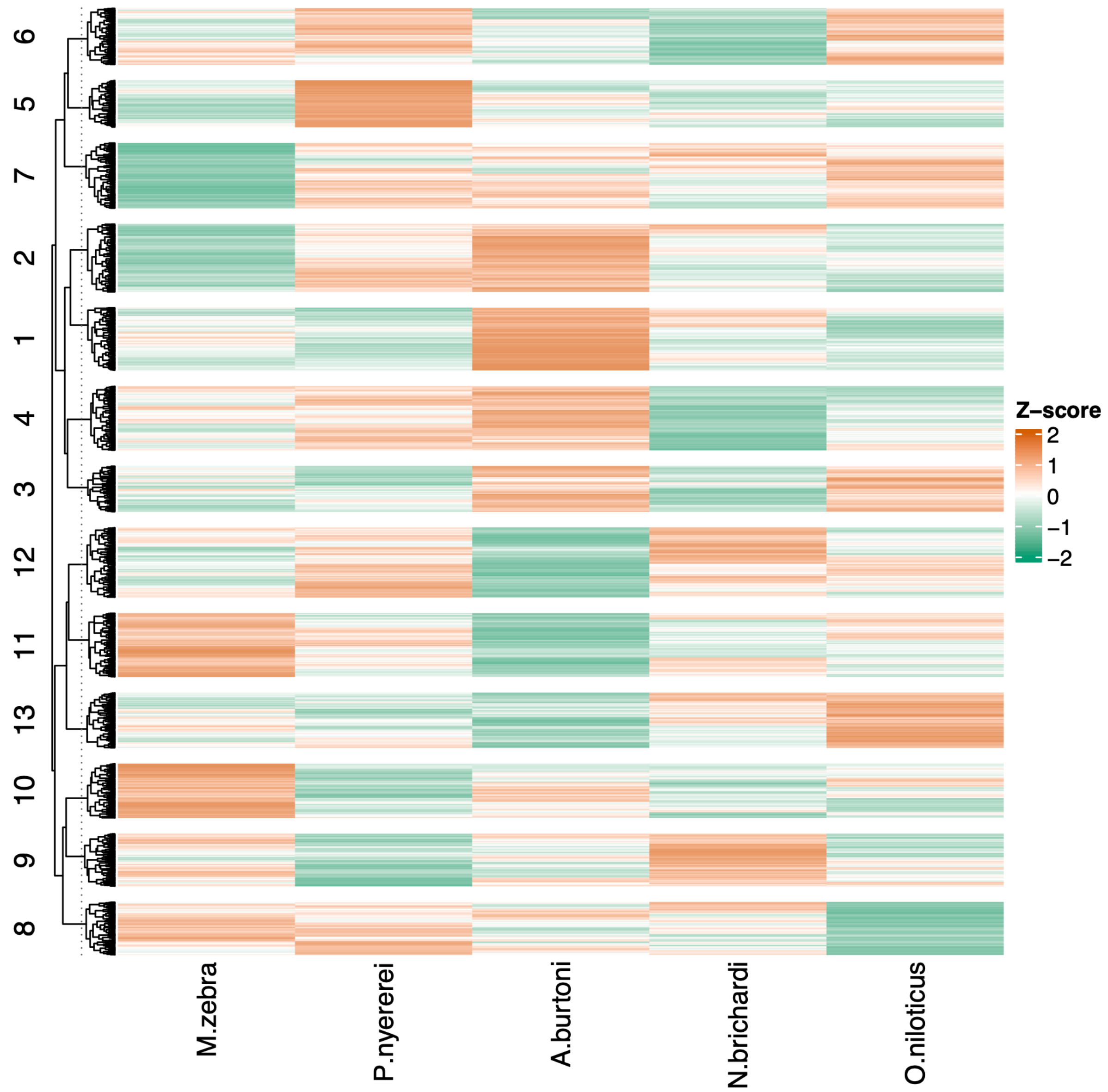

Fig. S9 – Clustering of TF activities in forebrain tissue across the five species. Each row represents a single, clustered TF, and each column a species. Mean TF bit-score of motif match across gene promoters (as a measure of activity) from HINT-ATAC [38] are *Z*-score transformed across rows. Green colour indicates low activity and orange colour indicates high activity.

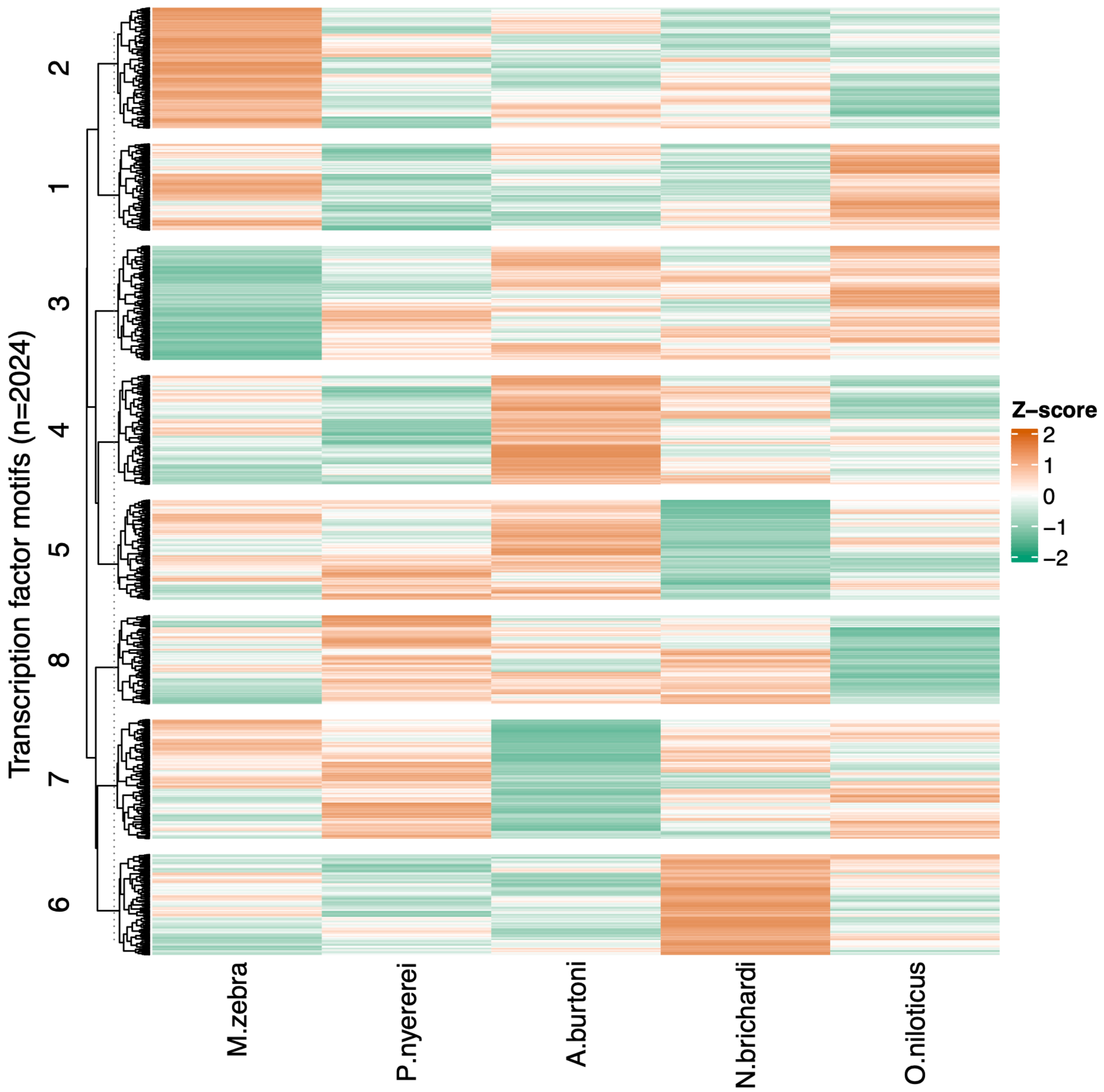

Fig. S10 – Clustering of TF activities in liver tissue across the five species.

Each row represents a single, clustered TF, and each column a species. Mean TF bit-score of motif match across gene promoters (as a measure of activity) from HINT-ATAC [38] are *Z*-score transformed across rows. Green colour indicates low activity and orange colour indicates high activity.

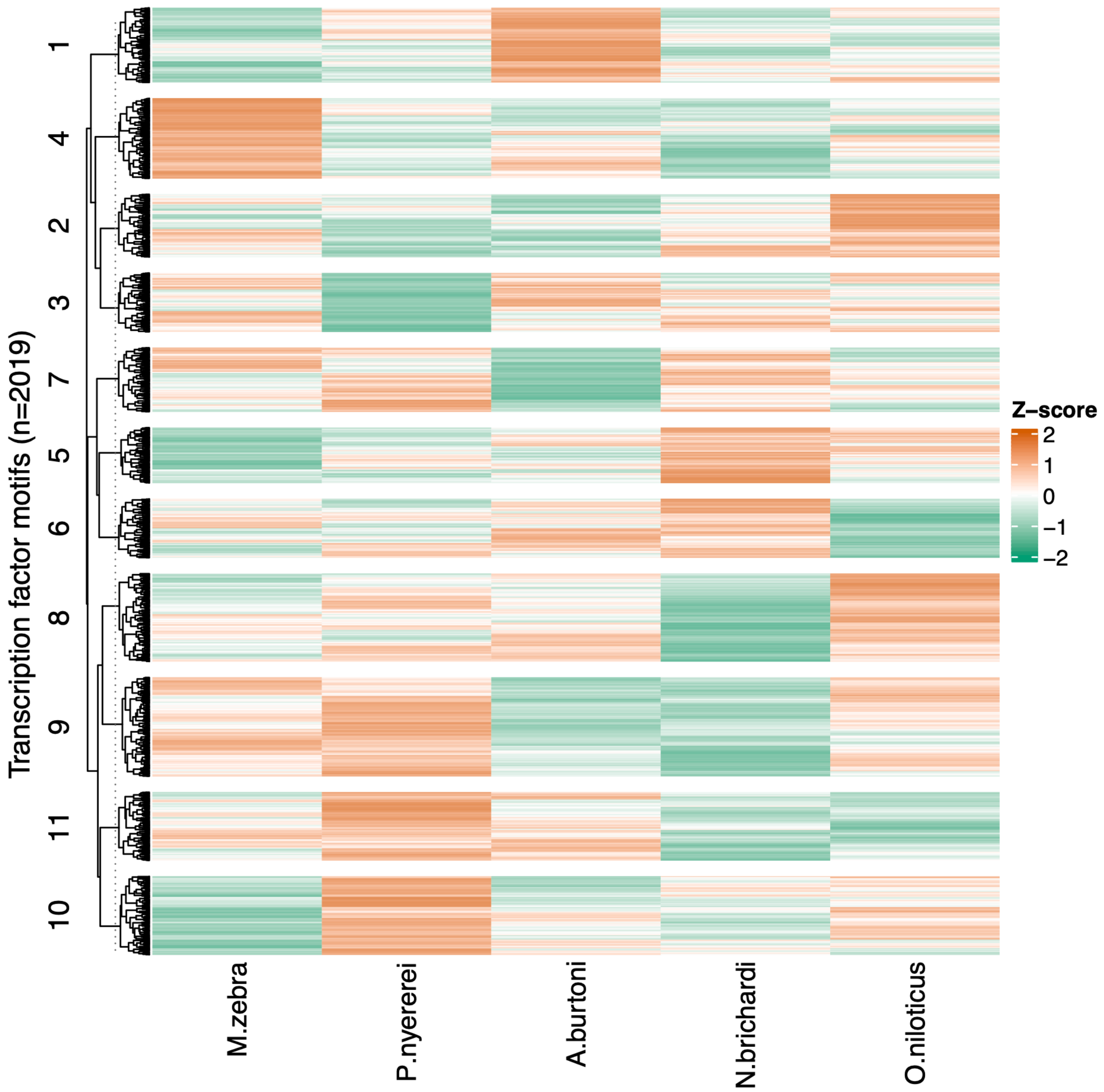

Fig. S11 – Clustering of TF activities in testis tissue across the five species. Each row represents a single, clustered TF, and each column a species. Mean TF bit-score of motif match across gene promoters (as a measure of activity) from HINT-ATAC [38] are *Z*-score transformed across rows. Green colour indicates low activity and orange colour indicates high activity.

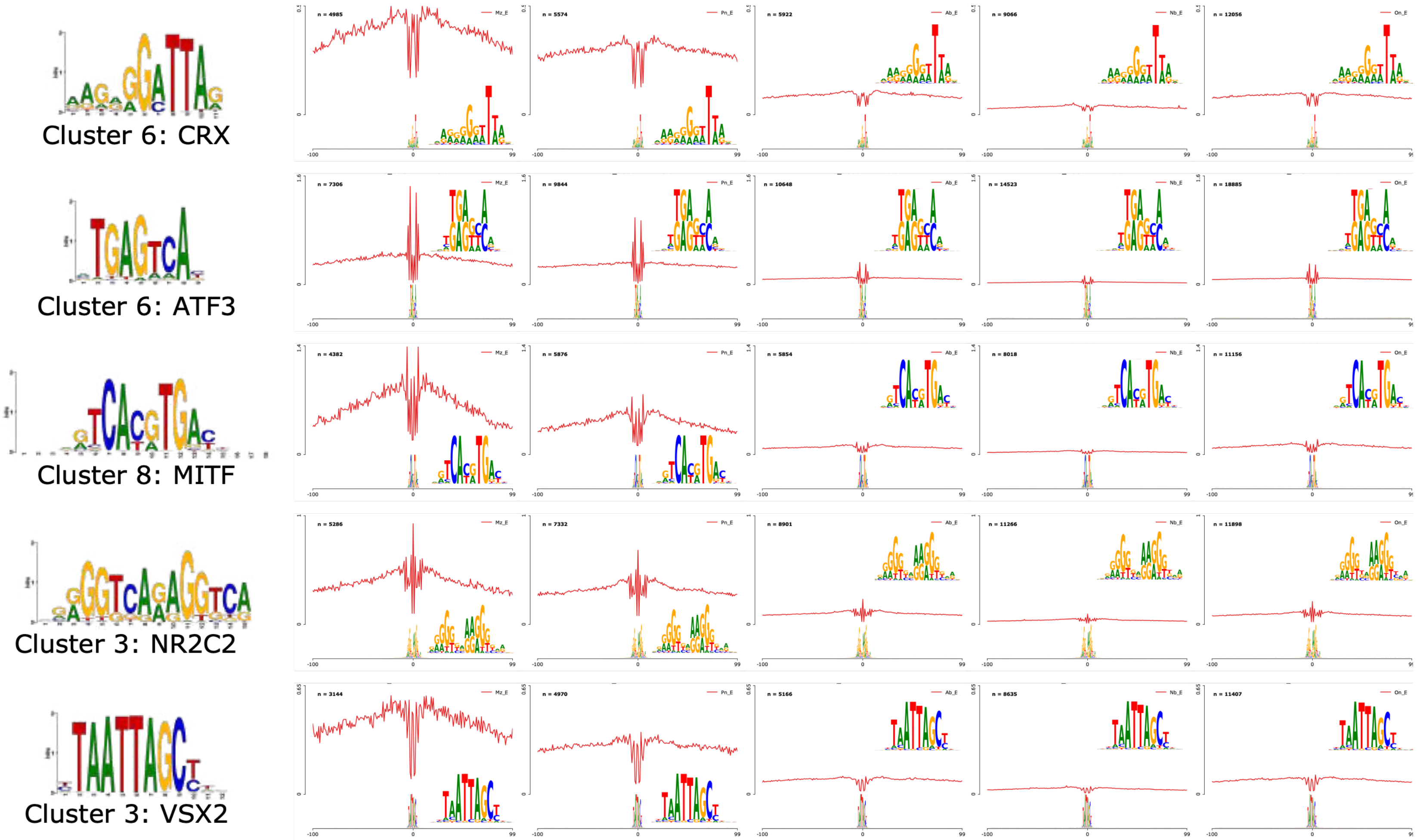

Fig. S12 – Activity profile of five eye-related TFs across retina tissue of five cichlid species. Line plot profile of the CRX, ATF3, MITF, NR2C2, and VSX2 TF footprints across retina tissue of five cichlid species (Mz – *M. zebra;* Pn – *P. nyererei;* Ab – *A.*

burtoni; Nb – *N. brichardi;* On – *O. niloticus*) gene promoter regions showing positional (x-axis) signal of activity (red line, y-axis) centred on the footprint (at position 0) with flanking sequence. Number (n=) of footprints profiled for each TF shown on top left of each plot. Expanded TF footprint shown with each line plot and original position weight matrices (PWM) of input motif used for profiling the site shown to left, with retina cluster assignment (from Fig. 2C and Fig. 2D).

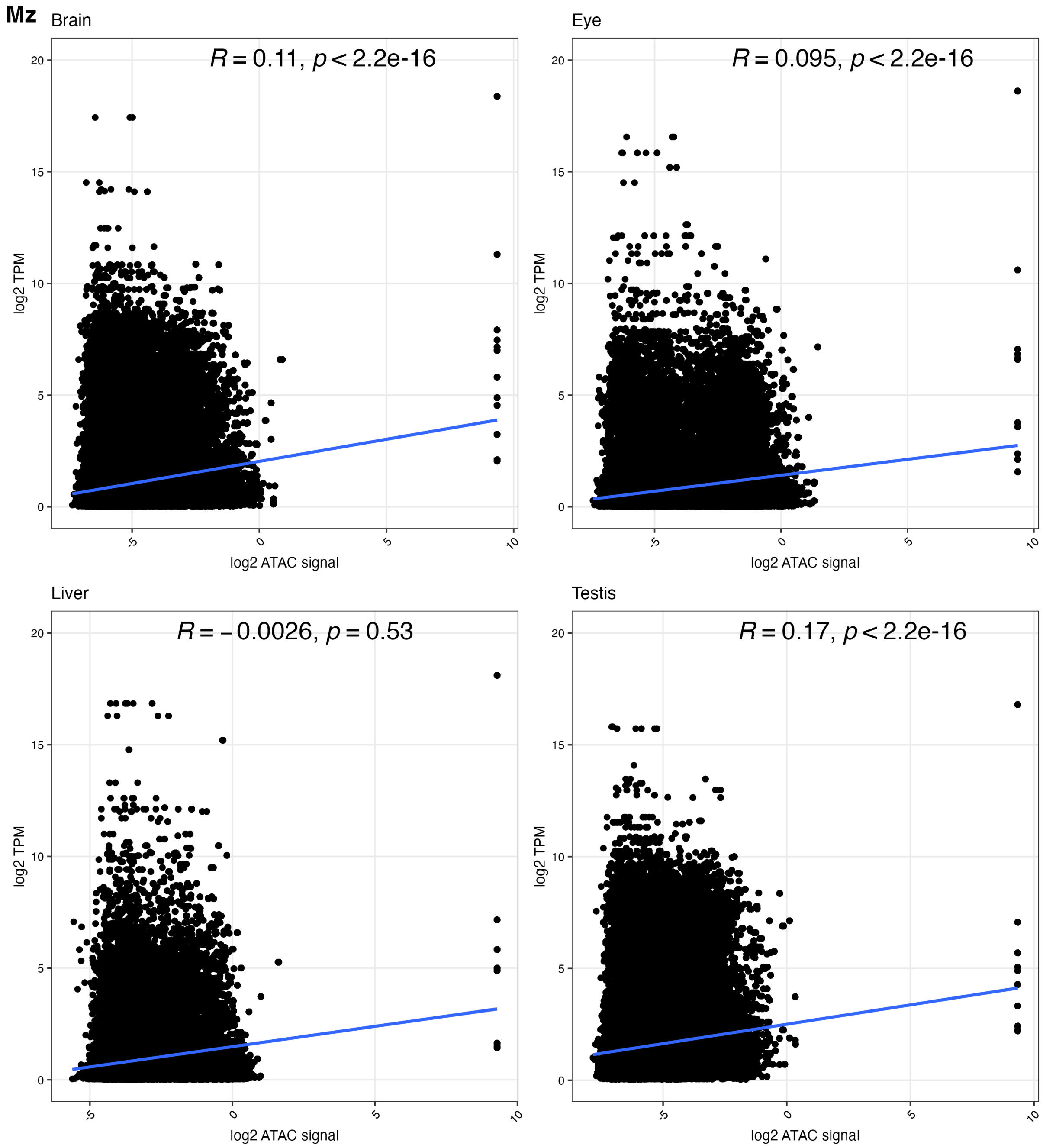

Fig. S13 – Correlation of accessible peaks accounting for gene expression change in *M. zebra* tissues. Average log2 gene expression as transcripts per million (TPM, y-axis) and average log2 ATAC signal as gene promoter peak counts (x-axis) for each gene across biological replicates, split by tissue (Brain = Forebrain and Eye = Retina). Correlation co-efficient (*R,* blue trendline) and *p-value* shown for each category.

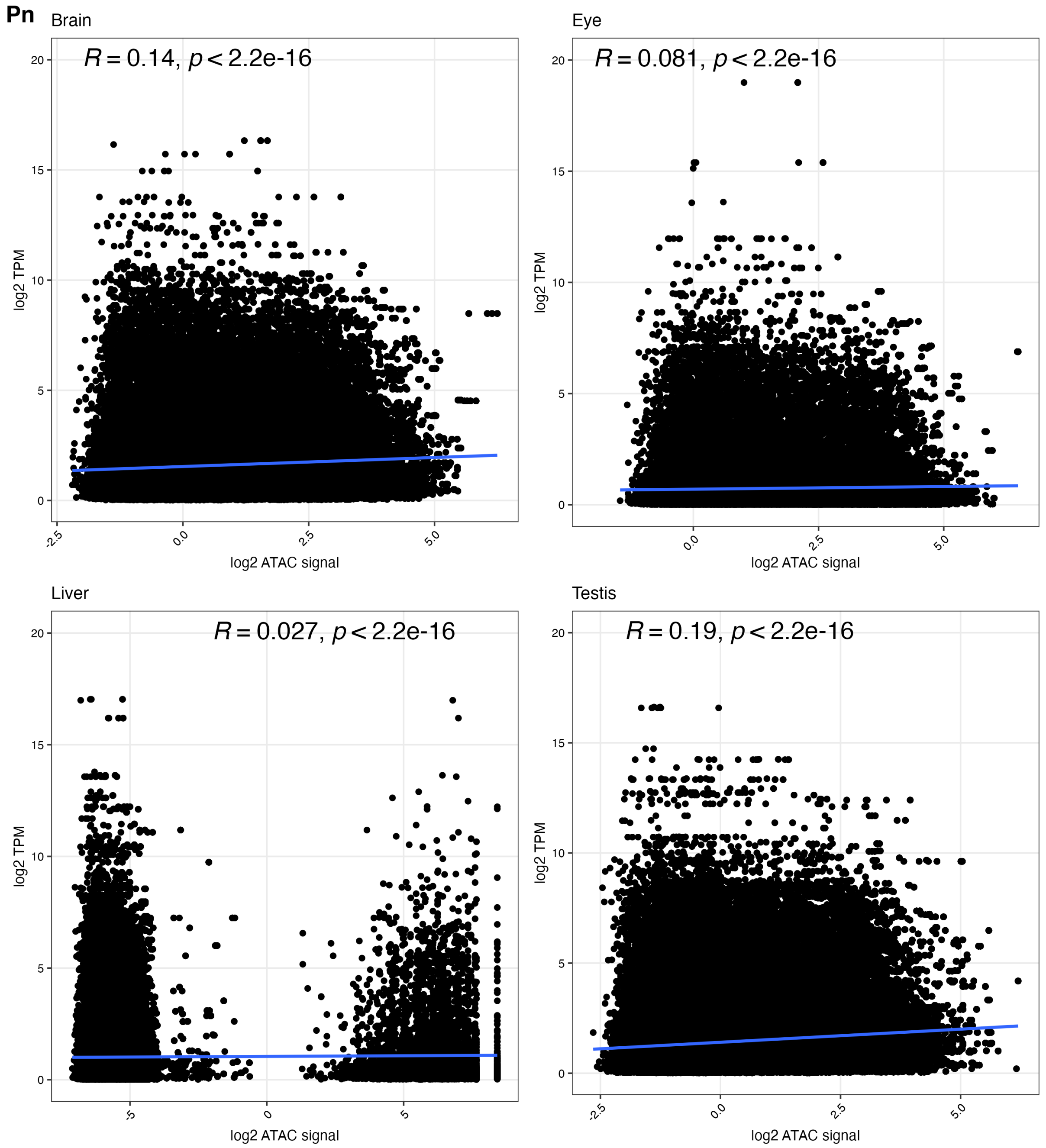

Fig. S14 – Correlation of accessible peaks accounting for gene expression change in *P. nyererei* tissues. Average log2 gene expression as transcripts per million (TPM, y-axis) and average log2 ATAC signal as gene promoter peak counts (x-axis) for each gene across biological replicates, split by tissue (Brain = Forebrain and Eye = Retina). Correlation co-efficient (*R,* blue trendline) and *p-value* shown for each category.

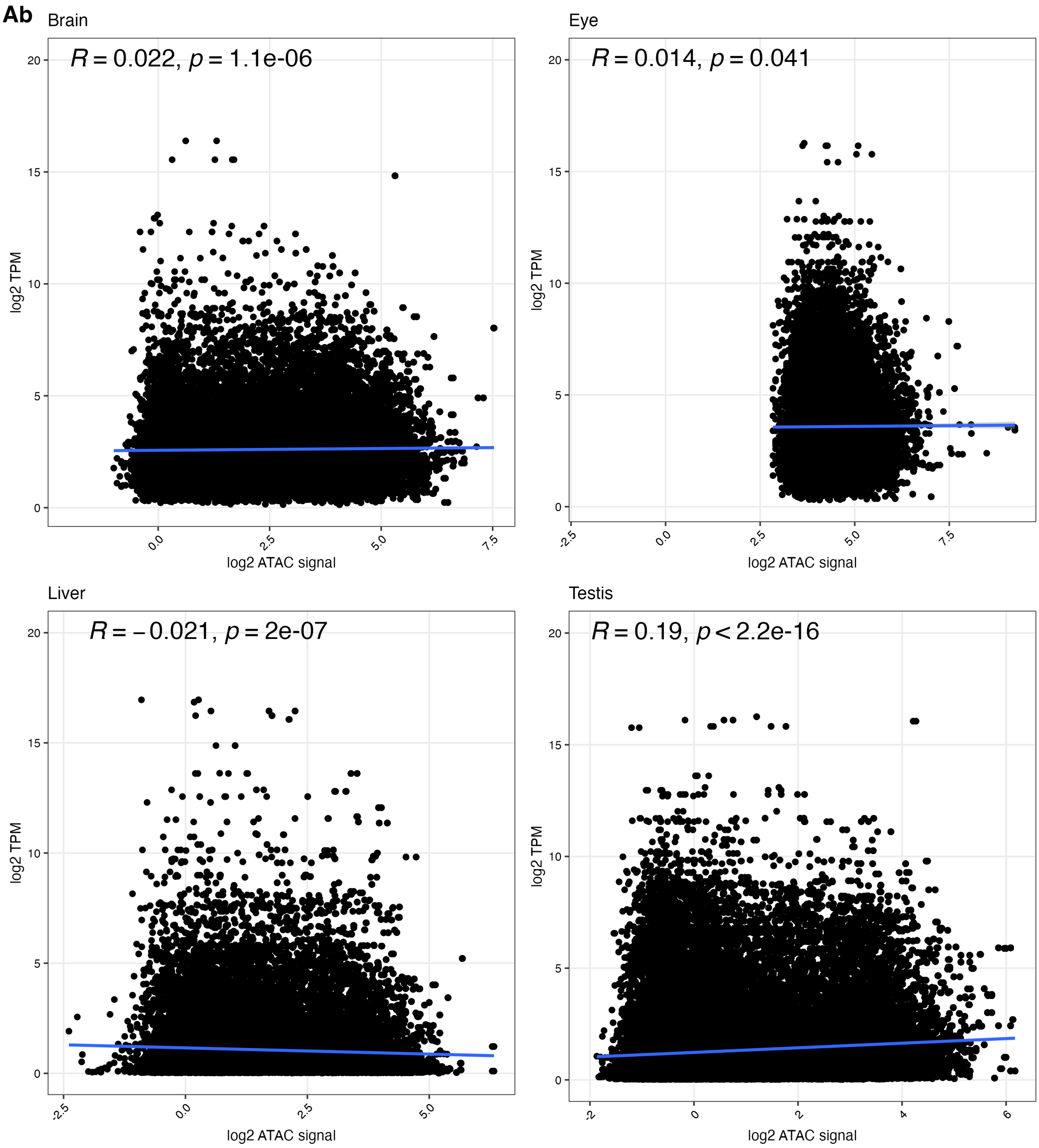

Fig. S15 – Correlation of accessible peaks accounting for gene expression change in *A. burtoni* tissues. Average log2 gene expression as transcripts per million (TPM, y-axis) and average log2 ATAC signal as gene promoter peak counts (x-axis) for each gene across biological replicates, split by tissue (Brain = Forebrain and Eye = Retina). Correlation co-efficient (*R,* blue trendline) and *p-value* shown for each category.

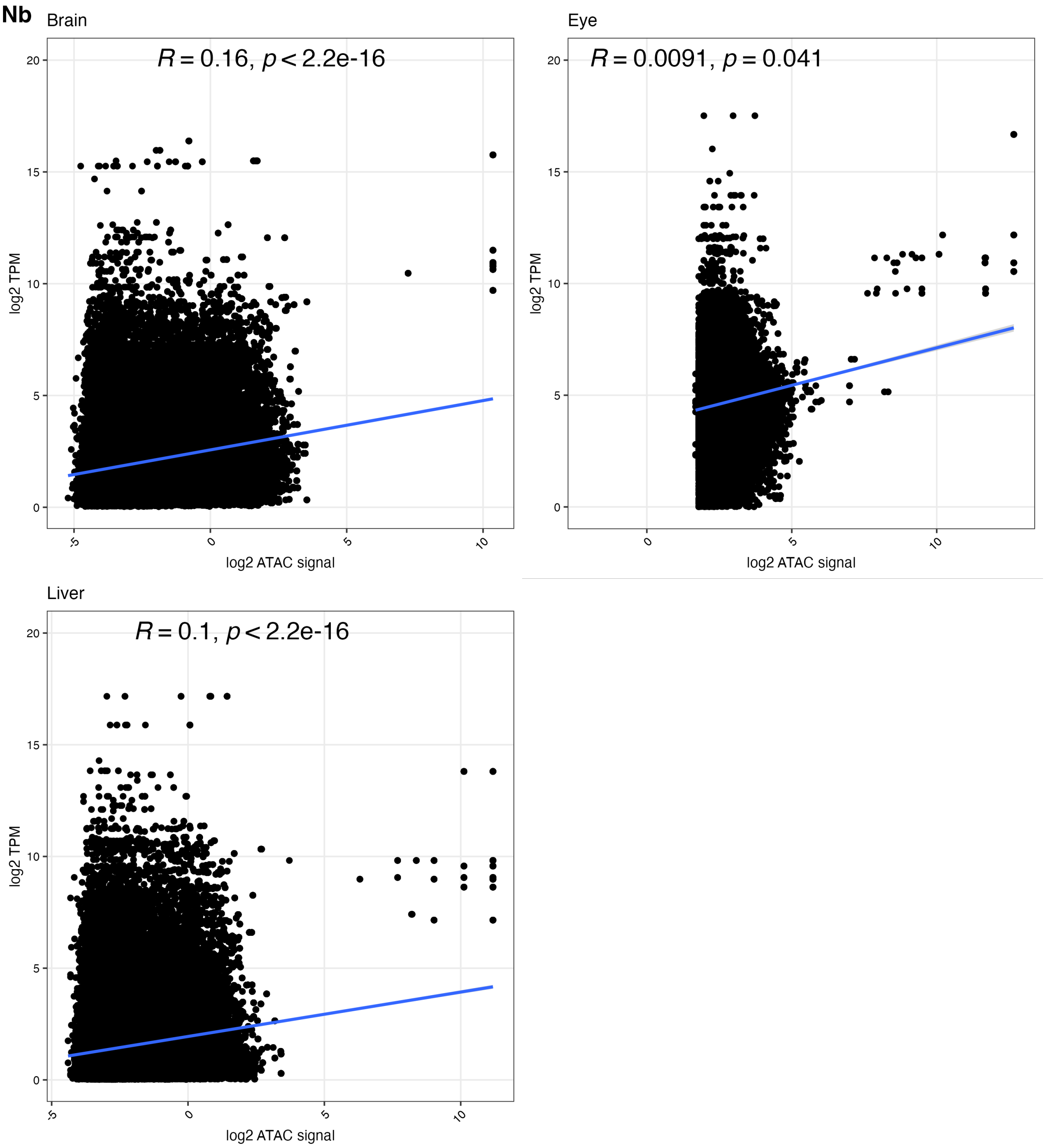

Fig. S16 – Correlation of accessible peaks accounting for gene expression change in *N. brichardi* tissues. Average log*2* gene expression as transcripts per million (TPM, y-axis) and average log2 ATAC signal as gene promoter peak counts (x-axis) for each gene across biological replicates, split by tissue (Brain = Forebrain and Eye = Retina). Correlation co-efficient (*R,* blue trendline) and *p-value* shown for each category.

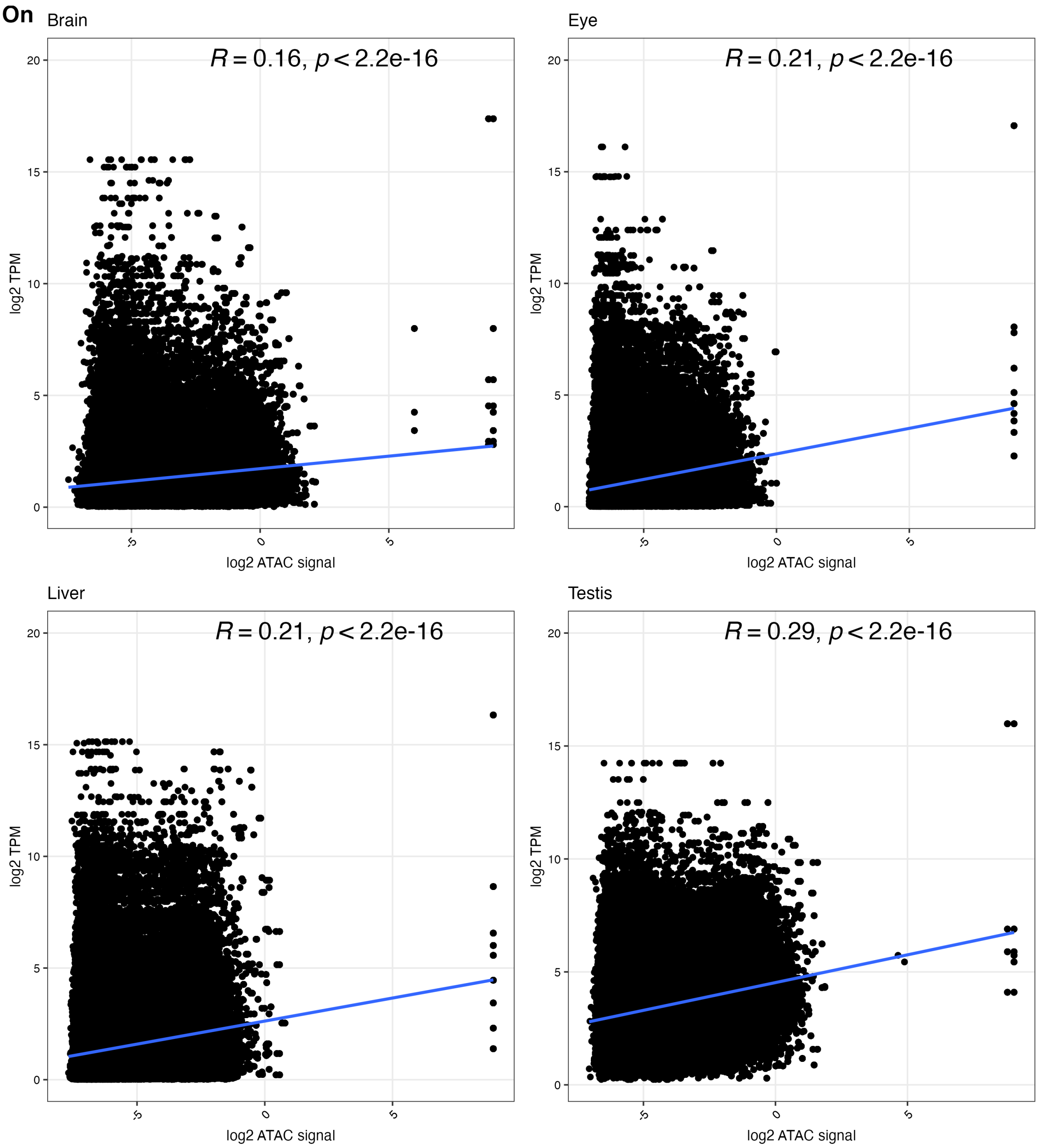

Fig. S17 – Correlation of accessible peaks accounting for gene expression change in *O. niloticus* tissues. Average log*2* gene expression as transcripts per million (TPM, y-axis) and average log2 ATAC signal as gene promoter peak counts (x-axis) for each gene across biological replicates, split by tissue (Brain = Forebrain and Eye = Retina). Correlation co-efficient (*R,* blue trendline) and *p-value* shown for each category.

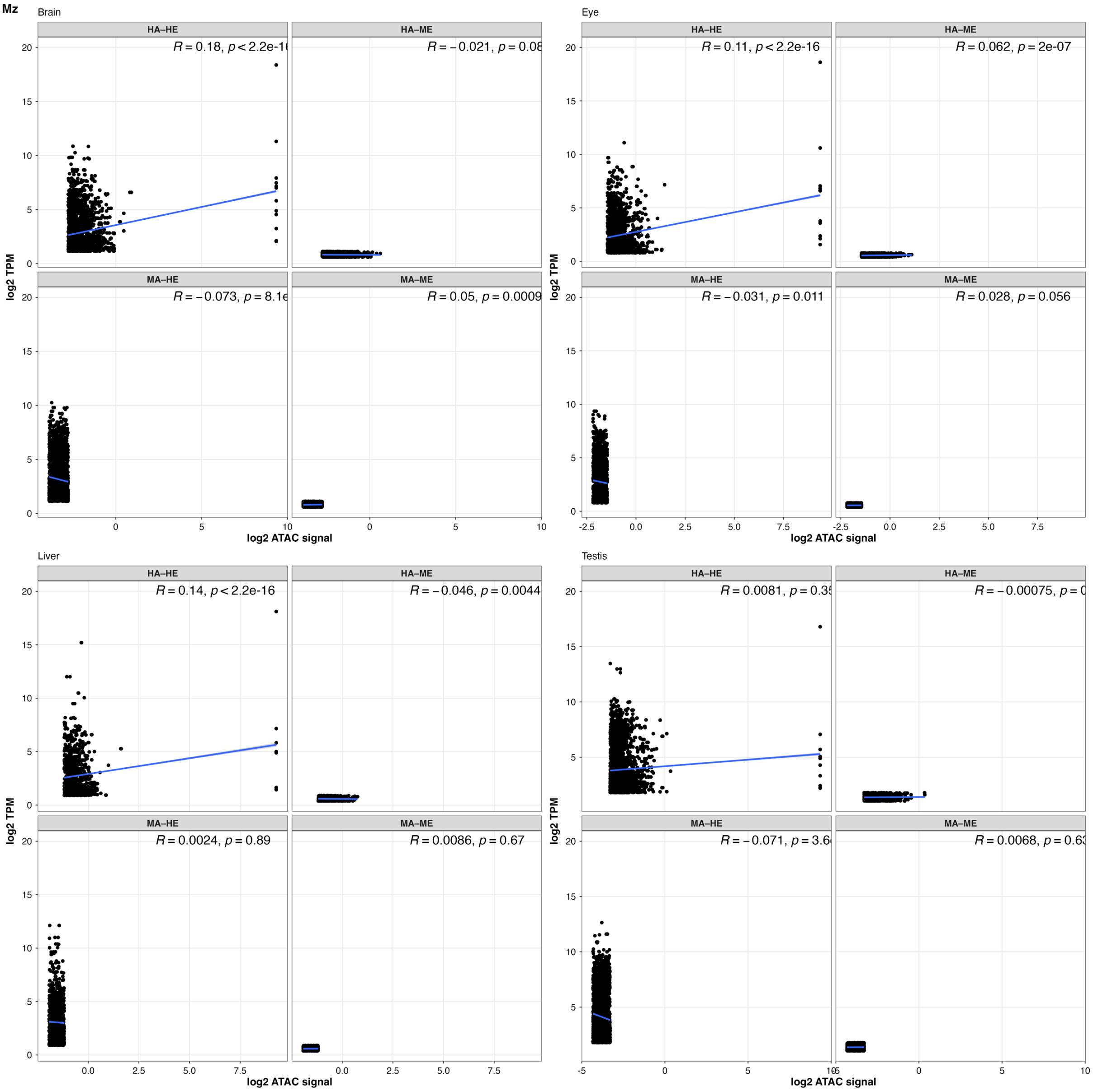

Fig. S18 – Categorised correlation of accessible peaks accounting for gene expression change in *M. zebra* tissues. Tissue-specific average *log2* gene expression as transcripts per million (TPM, y-axis) and average *log2* ATAC signal as gene promoter peak counts (x-axis) for each gene across same tissue biological replicates (Brain = Forebrain and Eye = Retina), split by high accessibility and high expression (HA-HE - both average TPM and ATAC signal values are more than the 70th percentiles), high accessibility and medium-low expression (HA-ME - ATAC signal values more than the 70th percentiles and average TPM less than the 50th

percentile), medium-low accessibility and high expression (MA-HE - ATAC signal values less than the 50th percentiles and average TPM more than the 70th percentile), and medium-low accessibility and medium-low expression (MA-ME - average ATAC signal count less than the 50th percentile and average TPM more than the 70th percentile). Correlation co-efficient (*R,* blue trendline) and *p-value* shown for each category.

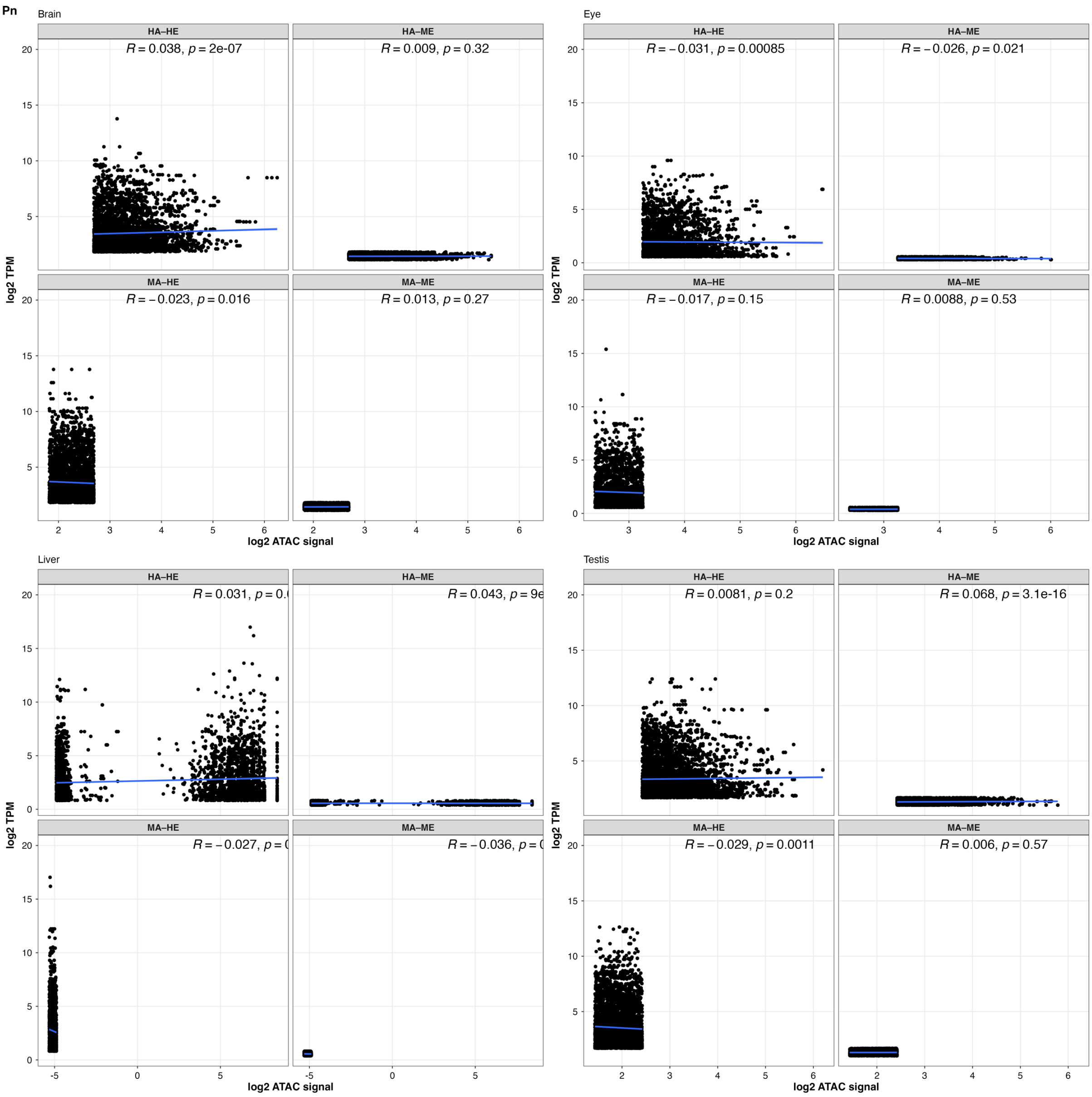

Fig. S19 – Categorised correlation of accessible peaks accounting for gene expression change in *P. nyererei* tissues. Tissue-specific average *log2* gene expression as transcripts per million (TPM, y-axis) and average *log2* ATAC signal as gene promoter peak counts (x-axis) for each gene across same tissue biological replicates (Brain = Forebrain and Eye = Retina), split by high accessibility and high expression (HA-HE - both average TPM and ATAC signal values are more than the 70th percentiles), high accessibility and medium-low expression (HA-ME - ATAC signal values more than the 70th percentiles and average TPM less than the 50th

percentile), medium-low accessibility and high expression (MA-HE - ATAC signal values less than the 50th percentiles and average TPM more than the 70th percentile), and medium-low accessibility and medium-low expression (MA-ME - average ATAC signal count less than the 50th percentile and average TPM more than the 70th percentile). Correlation co-efficient (*R,* blue trendline) and *p-value* shown for each category.

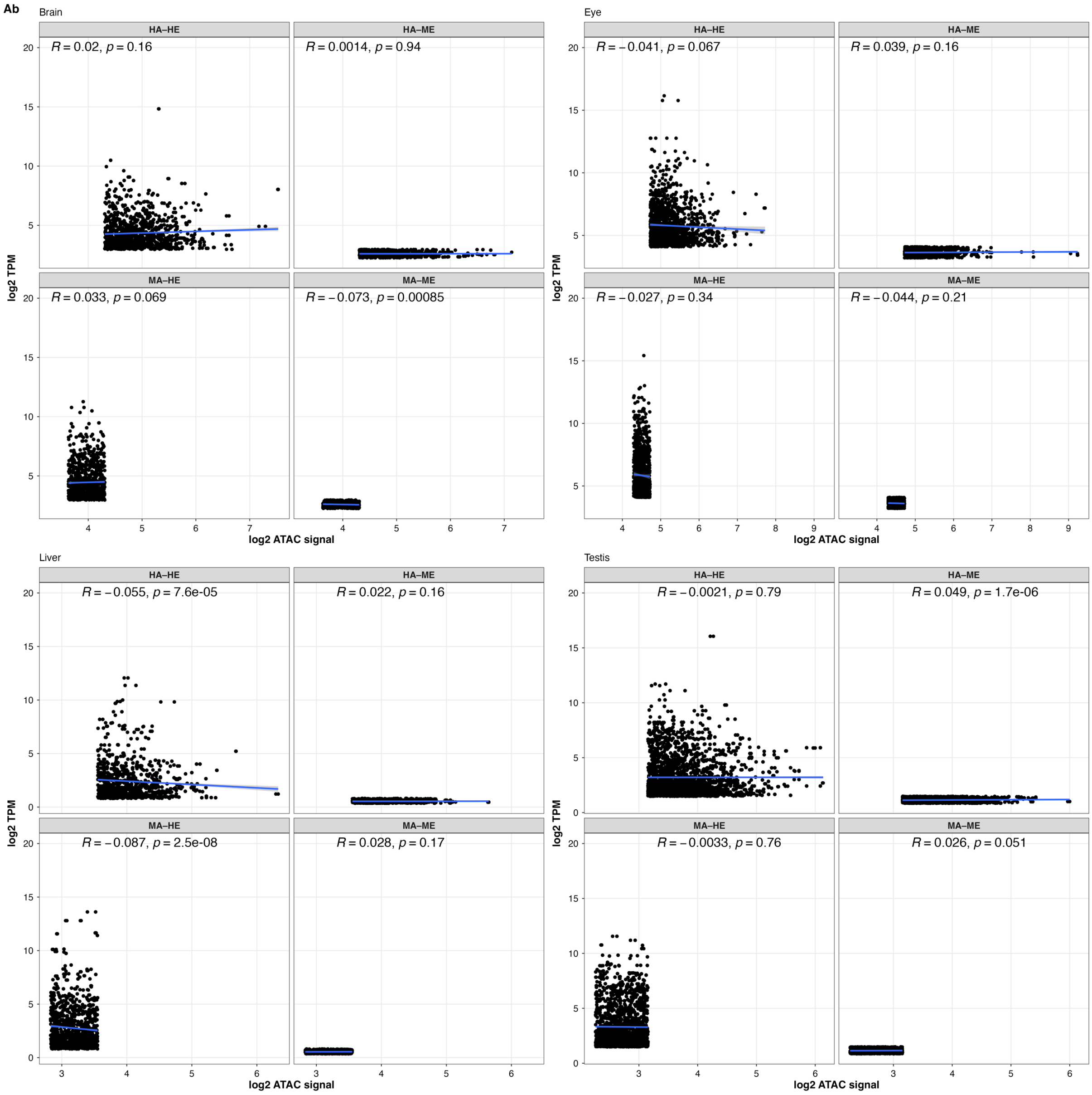

Fig. S20 – Categorised correlation of accessible peaks accounting for gene expression change in *A. burtoni* tissues. Tissue-specific average *log2* gene expression as transcripts per million (TPM, y-axis) and average *log2* ATAC signal as gene promoter peak counts (x-axis) for each gene across same tissue biological replicates (Brain = Forebrain and Eye = Retina), split by high accessibility and high expression (HA-HE - both average TPM and ATAC signal values are more than the 70th percentiles), high accessibility and medium-low expression (HA-ME - ATAC signal values more than the 70th percentiles and average TPM less than the 50th

percentile), medium-low accessibility and high expression (MA-HE - ATAC signal values less than the 50th percentiles and average TPM more than the 70th percentile), and medium-low accessibility and medium-low expression (MA-ME - average ATAC signal count less than the 50th percentile and average TPM more than the 70th percentile). Correlation co-efficient (*R,* blue trendline) and *p-value* shown for each category.

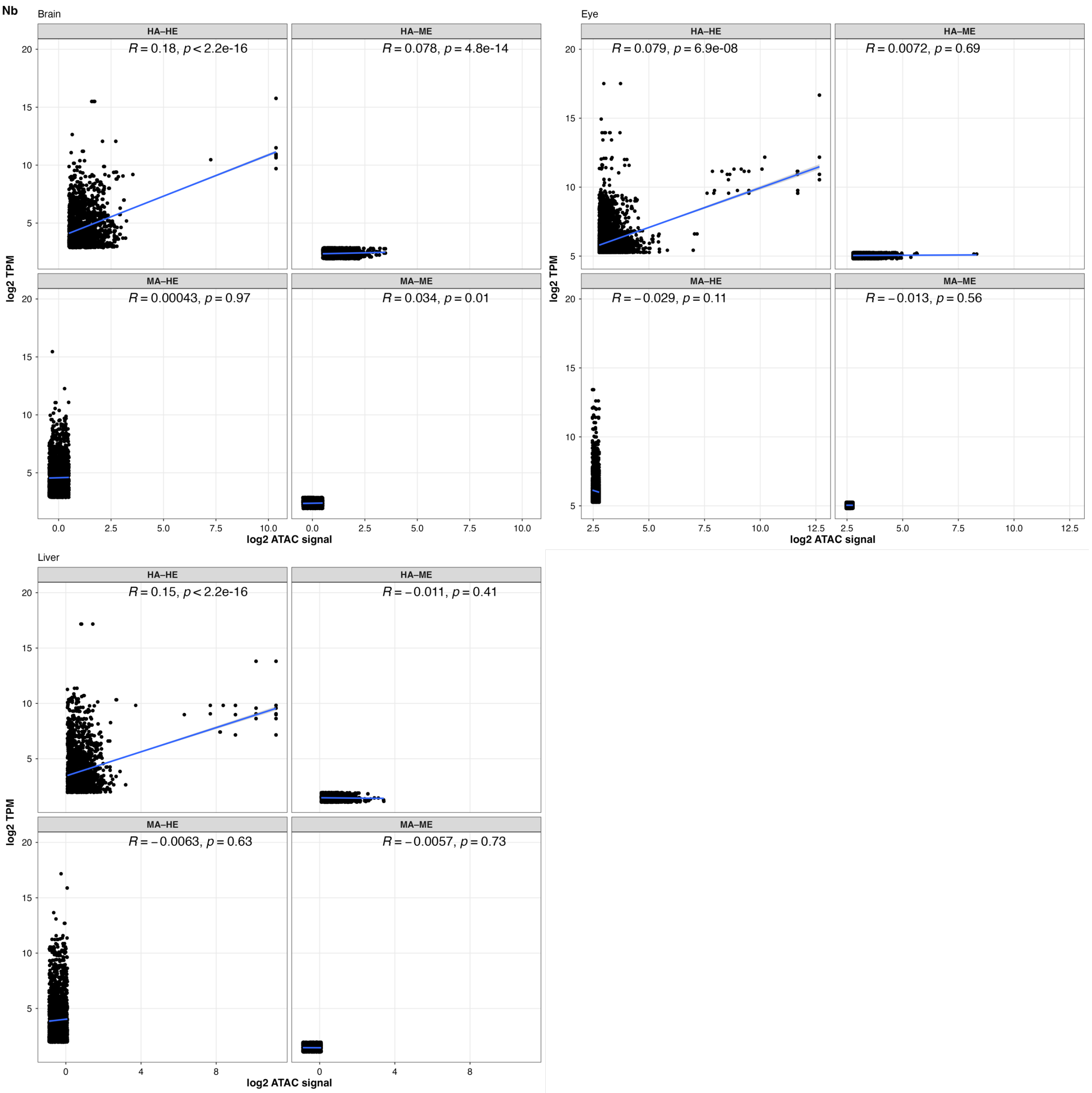

Fig. S21 – Categorised correlation of accessible peaks accounting for gene expression change in *N. brichardi* tissues. Tissue-specific average *log2* gene expression as transcripts per million (TPM, y-axis) and average *log2* ATAC signal as gene promoter peak counts (x-axis) for each gene across same tissue biological replicates (Brain = Forebrain and Eye = Retina), split by high accessibility and high expression (HA-HE - both average TPM and ATAC signal values are more than the 70th percentiles), high accessibility and medium-low expression (HA-ME - ATAC signal values more than the 70th percentiles and average TPM less than the 50th

percentile), medium-low accessibility and high expression (MA-HE - ATAC signal values less than the 50th percentiles and average TPM more than the 70th percentile), and medium-low accessibility and medium-low expression (MA-ME - average ATAC signal count less than the 50th percentile and average TPM more than the 70th percentile). Correlation co-efficient (*R,* blue trendline) and *p-value* shown for each category.

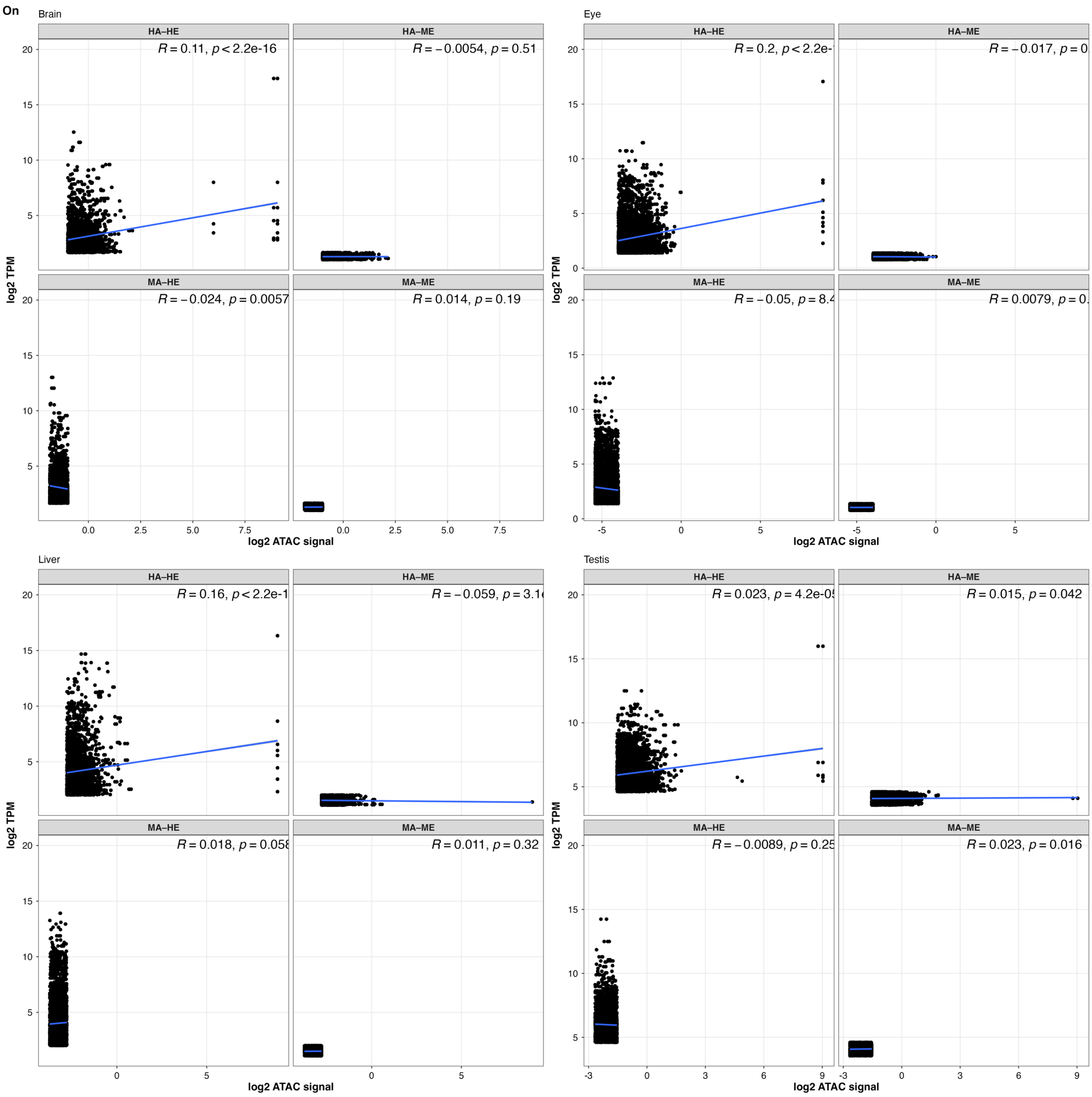

Fig. S22 – Categorised correlation of accessible peaks accounting for gene expression change in *O. niloticus* tissues. Tissue-specific average *log2* gene expression as transcripts per million (TPM, y-axis) and average *log2* ATAC signal as gene promoter peak counts (x-axis) for each gene across same tissue biological replicates (Brain = Forebrain and Eye = Retina), split by high accessibility and high expression (HA-HE - both average TPM and ATAC signal values are more than the 70th percentiles), high accessibility and medium-low expression (HA-ME - ATAC signal values more than the 70th percentiles and average TPM less than the 50th

percentile), medium-low accessibility and high expression (MA-HE - ATAC signal values less than the 50th percentiles and average TPM more than the 70th percentile), and medium-low accessibility and medium-low expression (MA-ME - average ATAC signal count less than the 50th percentile and average TPM more than the 70th percentile). Correlation co-efficient (*R,* blue trendline) and *p-value* shown for each category.

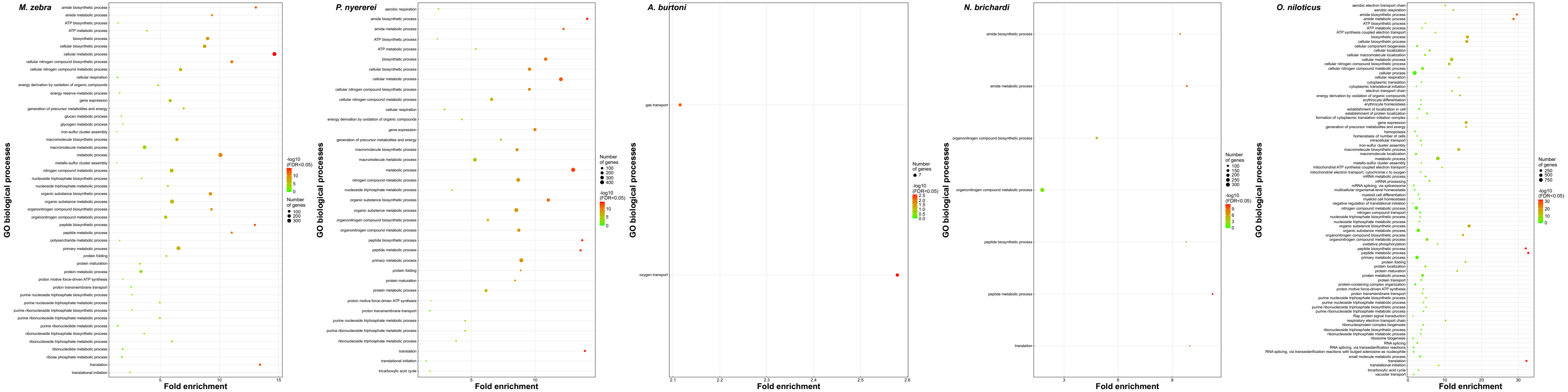

Fig. S23 – Gene Ontology (GO) enrichment of genes categorised as high accessibility (HA) and high expression (HE) in retina tissue of five cichlid species. GO biological processes enrichment of HA-HE genes in retina tissue of five cichlids. Circles show enriched biological processes (y-axis) of significance (-*log₁₀* FDR <0.05, heatmap to right) and -*log₁₀* FDR <0.05 (x-axis) values of each term across each tissue. Number of enriched genes for each term shown by size of each circle

(A)

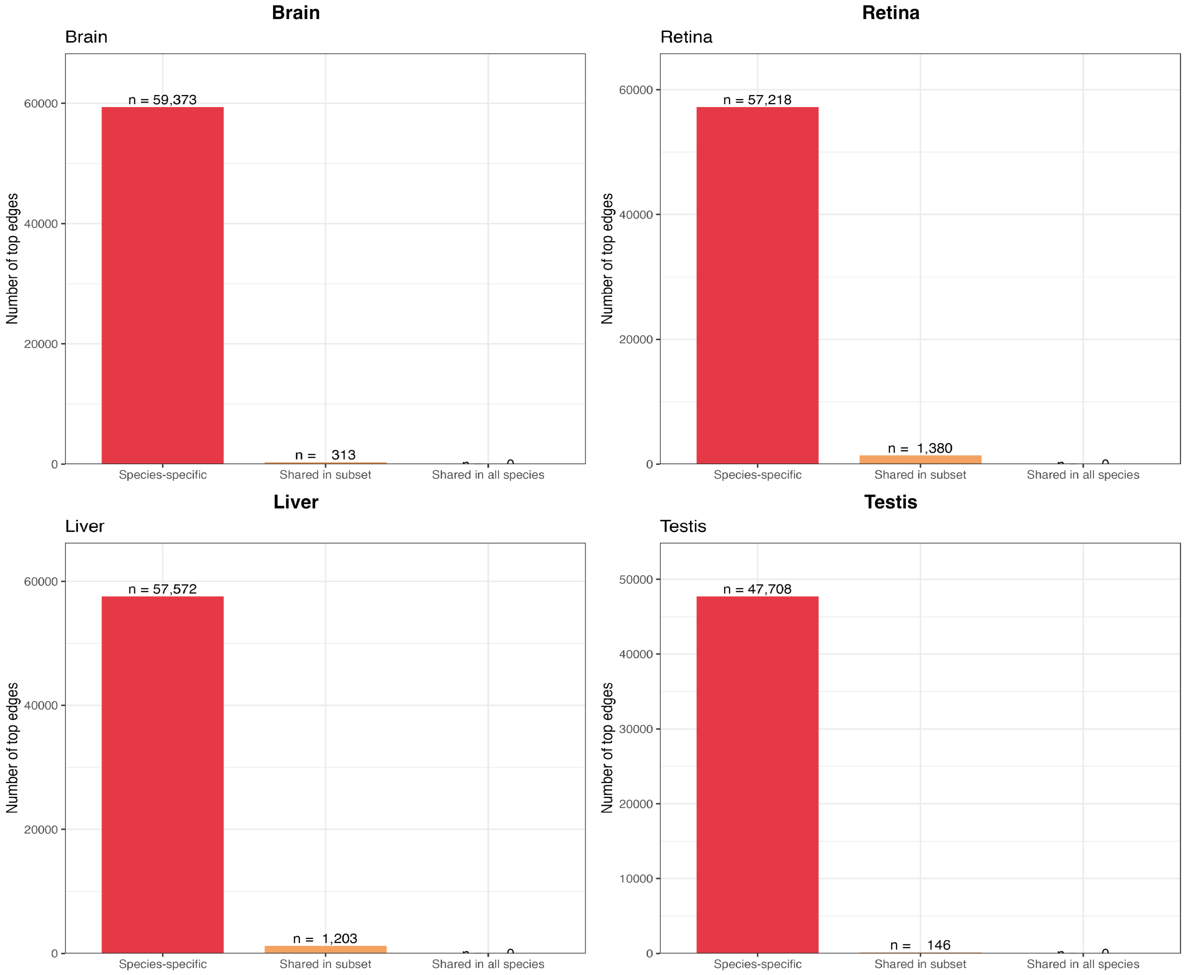
Brain
Brain
Number of top edges
n = 59,373
n = 313
Species-specific
Shared in subset
Shared in all species
Retina
Retina
n = 57,218
n = 1,380
Liver
Liver
n = 57,572
n = 1,203
Testis
Testis
n = 47,708
n = 146

(B)

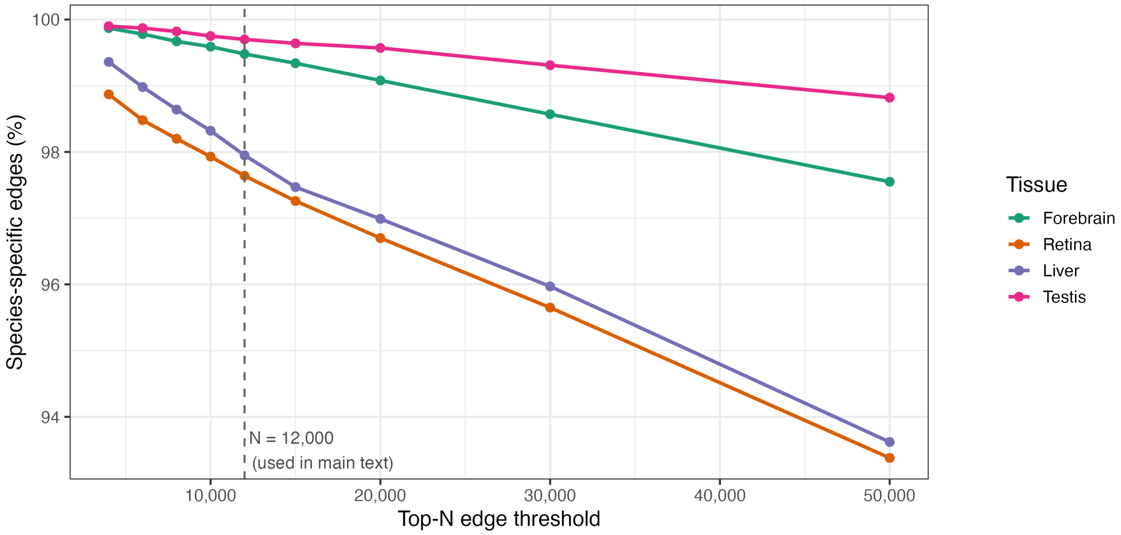
Species-specific edges (%)
Top-N edge threshold
N = 12,000
(used in main text)
Tissue
Forebrain
Retina
Liver
Testis

(C)

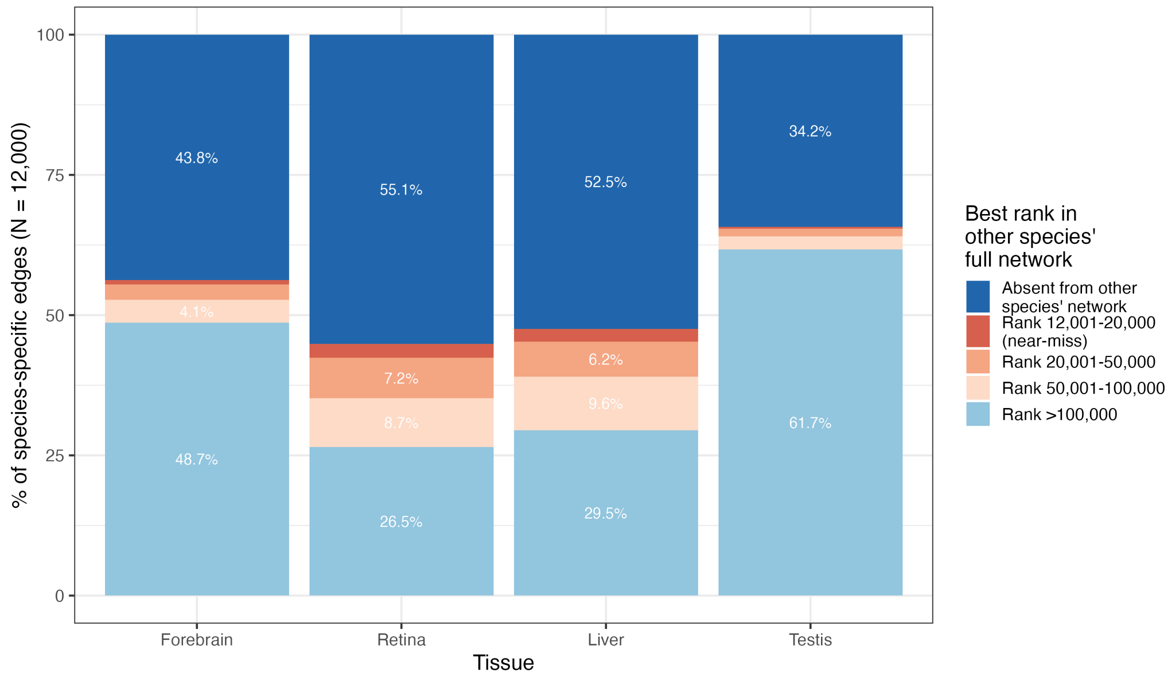

Fig. S24 – Conservation or divergence of top 12,000 expression only inferred GRN edges across tissues. (A) Distribution of top 12k GENIE3 edges classified as species-specific, shared in a subset of species, or shared in all five species for forebrain, retina, liver, and testis. Counts are shown on the bars and where absent, are zero-count categories. **(B)** Percentage of non-redundant top-*N* GENIE3 edges (union across the five species within each tissue) classified as species-specific, plotted across a range of thresholds (*N* = 4,000–50,000 edges per species-tissue network) for each of the four tissues (colour). The dashed vertical line marks *N* = 12,000, the threshold used throughout the main text and Fig. 6. **(C)** For edges classified as species-specific at the *N* = 12,000 threshold, the best (lowest) rank that same edge achieves in each other species' complete, untruncated GENIE3 network (up to ~11.6 million edges per network), summarised into bands: absent from every other species' network entirely (dark blue); ranked 12,001–20,000 — a "near-miss" that would be reclassified as shared if the threshold were relaxed (red); ranked 20,001–50,000 (orange); ranked 50,001–100,000 (light orange); or ranked beyond 100,000 (light

blue). Percentages are labelled within each stacked segment where ≥3%. Full underlying counts are given in Supplementary Table S17c.

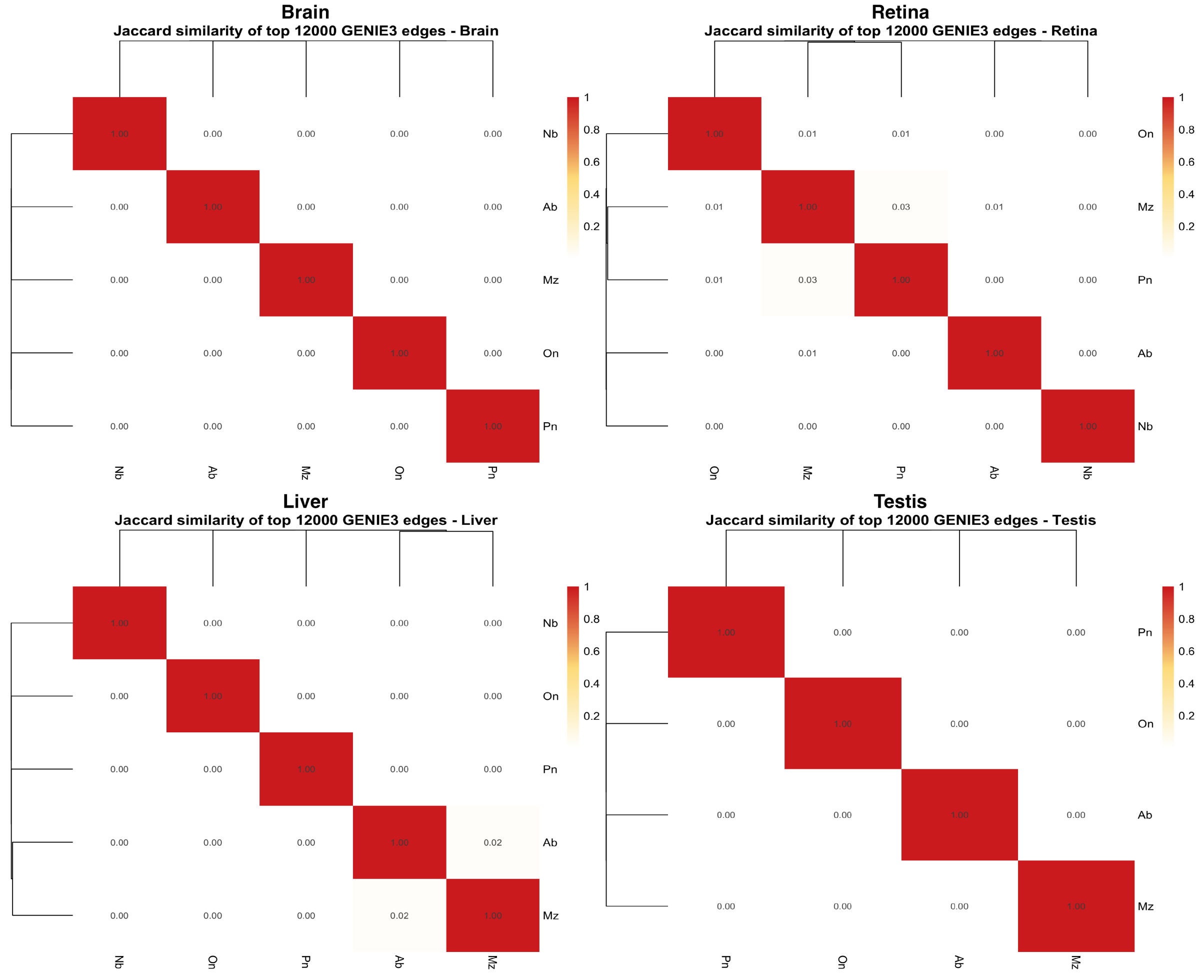

Fig. S25 - Jaccard similarity of the top 12,000 GENIE3 edges across species in forebrain, retina, liver, and testis. Heatmaps show pairwise Jaccard similarity among the top 12,000 GENIE3 edges for each tissue, with panels for forebrain, retina, liver, and testis. Species are ordered by hierarchical clustering on both axes, and the same species labels are shown on the x- and y-axes in each panel. Cell values are displayed within the matrix and range from 0 to 1, with darker red indicating higher similarity and pale/white shading indicating lower similarity.

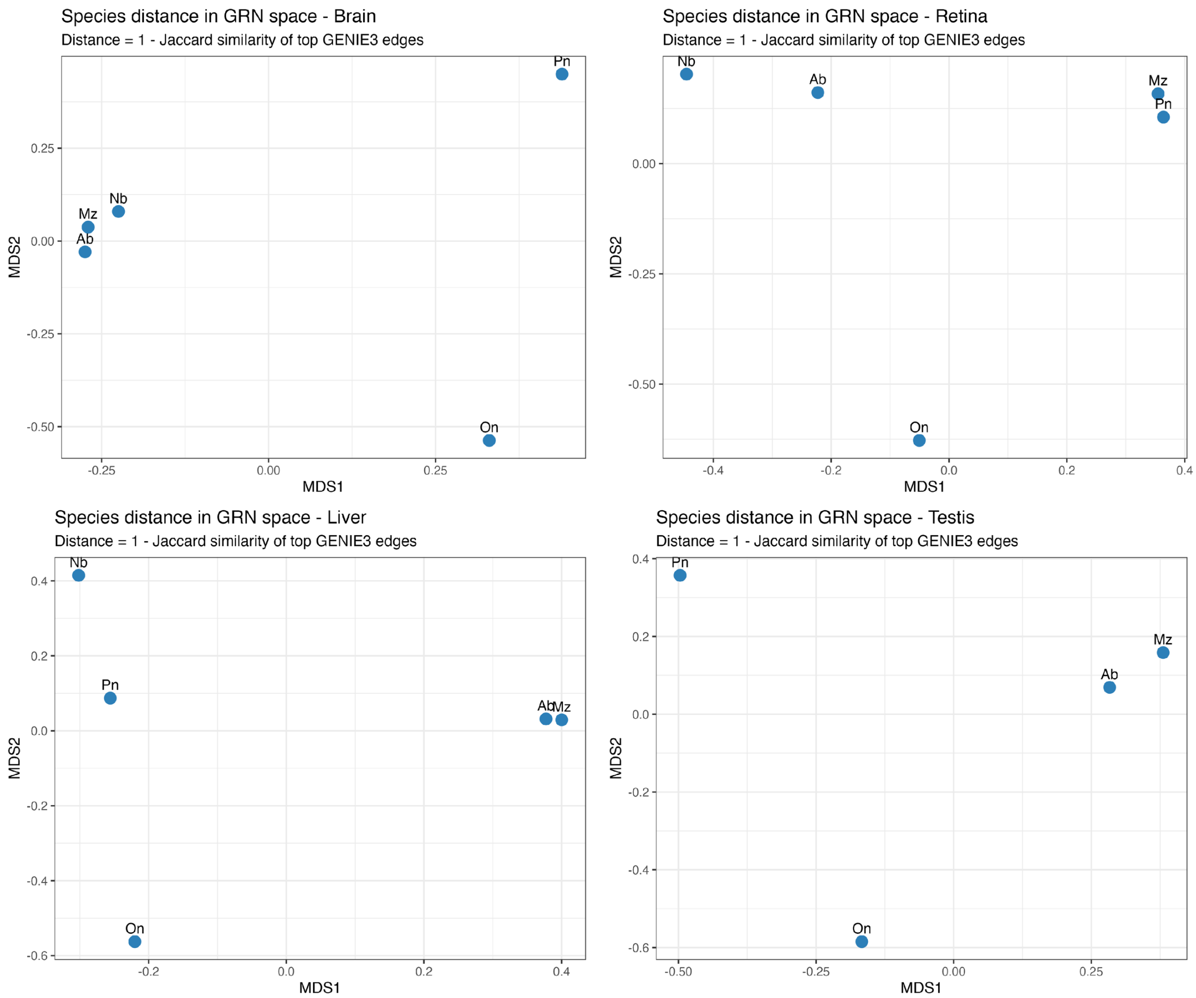

Fig. S26 - Species distance in GENIE3 edge GRN space across tissues. MDS plots of species distances in gene regulatory network (GRN) space for forebrain, retina, liver, and testis, where distance is defined as 1 minus Jaccard similarity of top GENIE3 edges. Points are coloured blue and labelled by species (Mz, Pn, Ab, Nb, On), plotted against MDS1 and MDS2 dimensions.

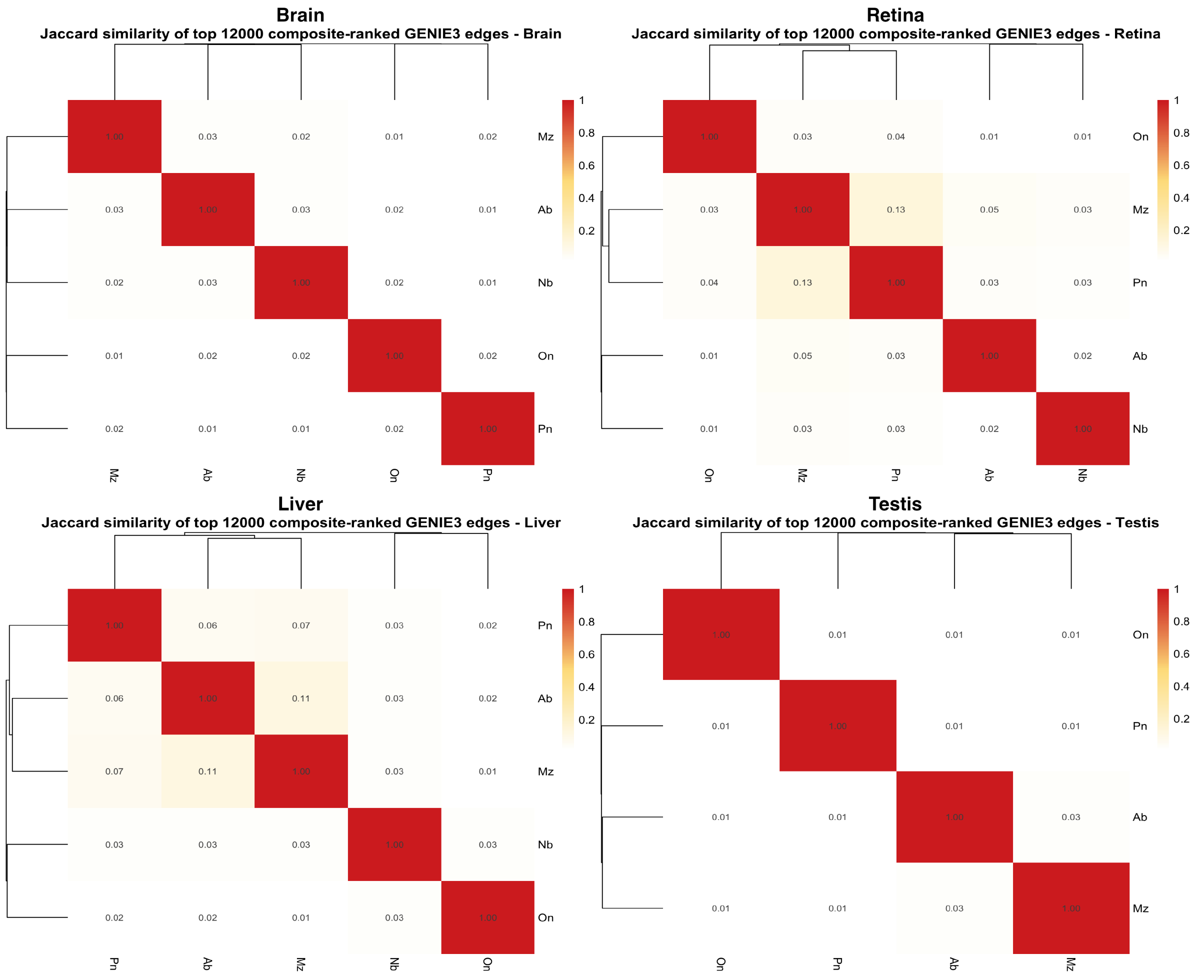

Fig. S27 - Jaccard similarity of top composite-ranked (motif-support integrated) GENIE3 edges across species. Heatmaps show pairwise Jaccard similarity among the top 12,000 composite-ranked (integrating motif-support) GENIE3 edges for forebrain, retina, liver, and testis (as separate panels). Species are ordered by hierarchical clustering on both axes, with identical species labels (Mz, Pn, Ab, Nb, On) on x- and y-axes per panel. Cell values are shown in the matrix, ranging from 0 to 1, with darker red for higher similarity and lighter shades for lower similarity.

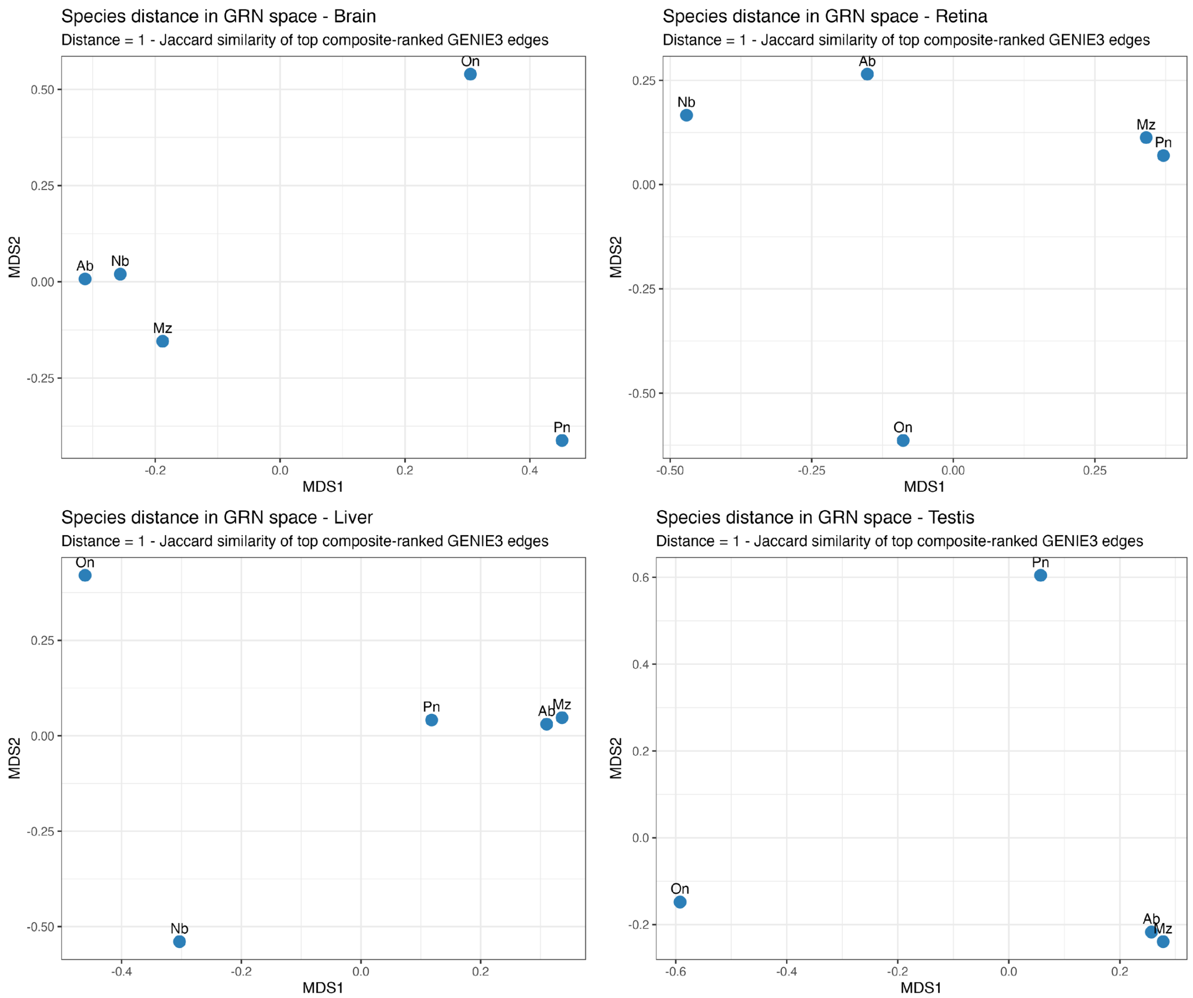

Fig. S28 - Species distance in composite-ranked (motif-support integrated) GENIE3 edge GRN space across tissues.

MDS plots of species distances in gene regulatory network (GRN) space for forebrain, retina, liver, and testis, where distance is defined as 1 minus Jaccard similarity of top composite-ranked (motif-support integrated) GENIE3 edges. Points are coloured blue and labelled by species (Mz, Pn, Ab, Nb, On), plotted against MDS1 and MDS2 dimensions.

Top rewired GENIE3 edges - Brain

CompositeWeight-based rewiring; Unified gene symbols used in labels where available

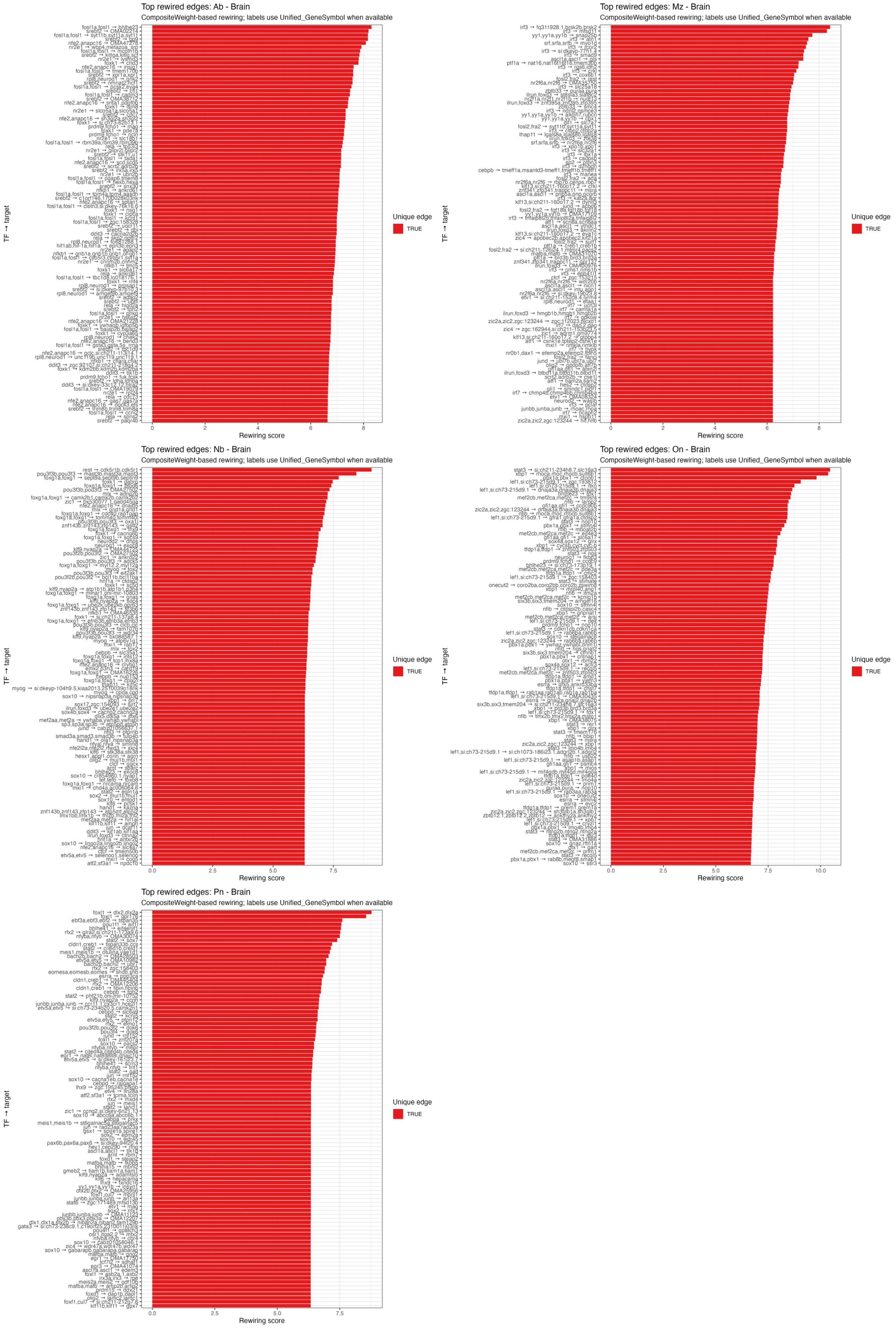

Fig. S29 - Top rewired GENIE3 edges in forebrain across species. Bar plots ranking the top composite-weighted rewired GENIE3 edges in brain for each of the five species. Bars are coloured red and show rewiring score on the x-axis; gene symbols (unified where available) label the y-axis. Red horizontal lines indicate edges confirmed as unique in motif-supported GENIE3 edges.

Top rewired GENIE3 edges - Retina

CompositeWeight-based rewiring; Unified gene symbols used in labels where available

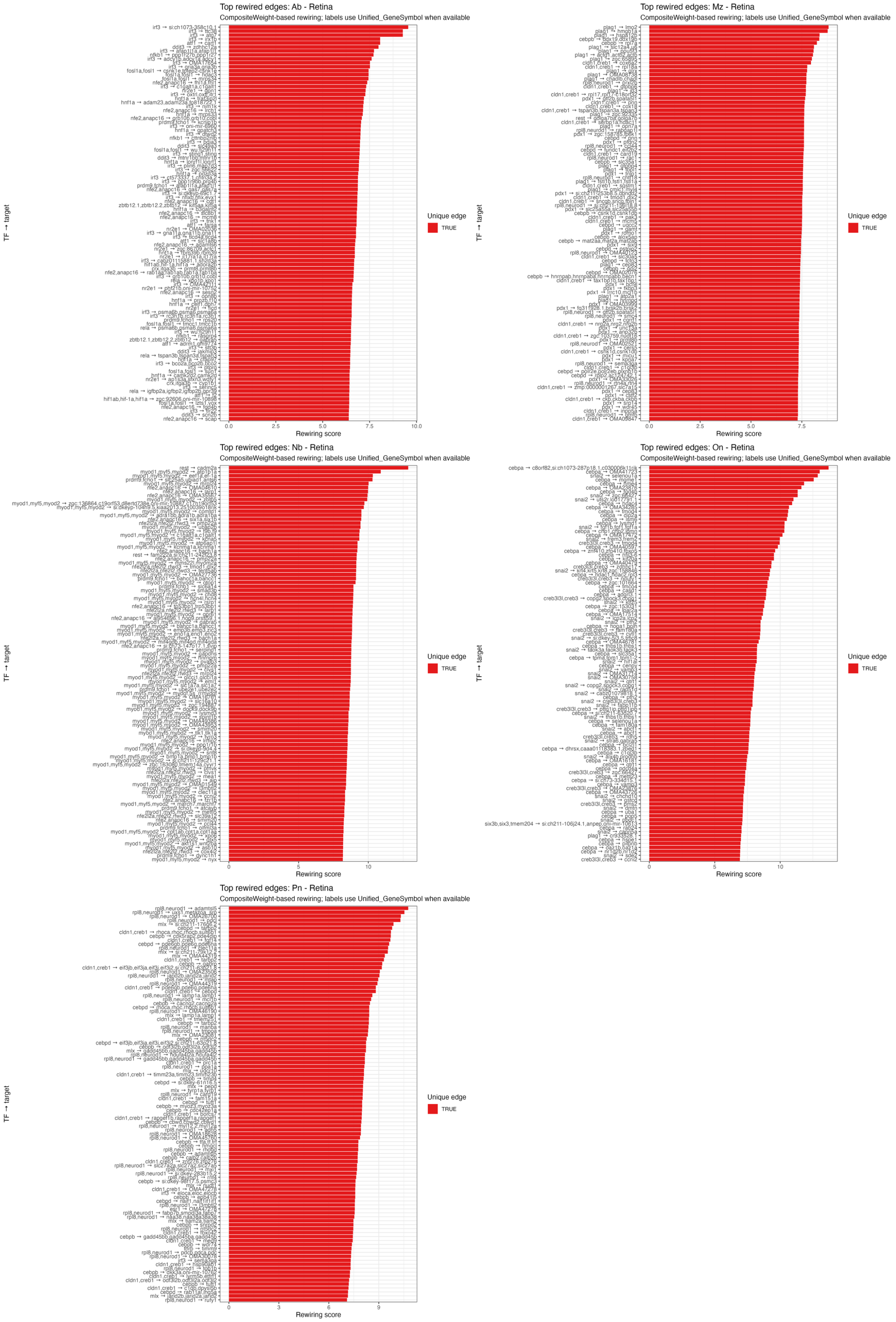

Fig. S30 - Top rewired GENIE3 edges in retina across species. Bar plots ranking the top composite-weighted rewired GENIE3 edges in retina for each of the five species. Bars are coloured red and show rewiring score on the x-axis; gene symbols (unified where available) label the y-axis. Red horizontal lines indicate edges confirmed as unique in motif-supported GENIE3 edges.

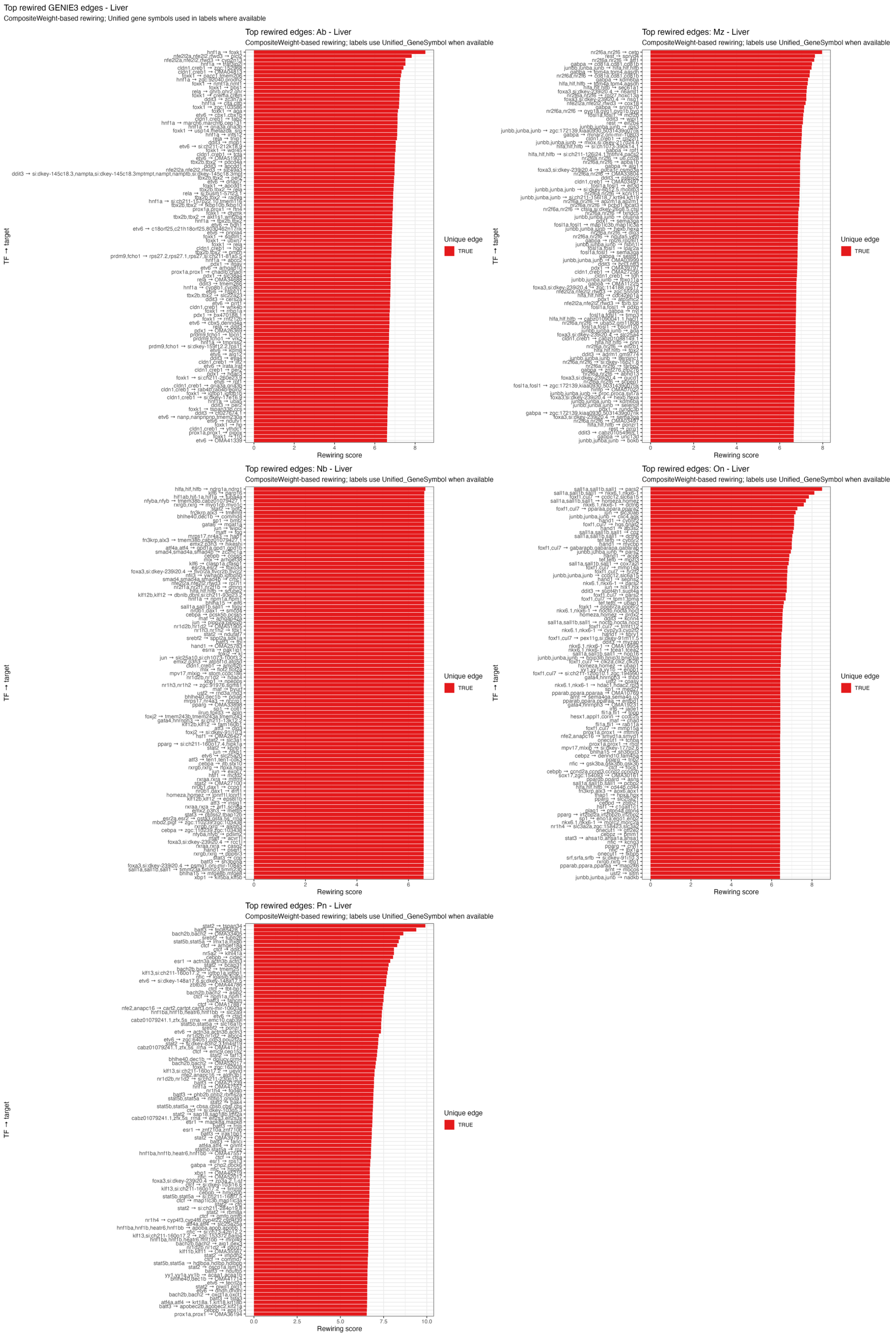
Top rewired GENIE3 edges - Liver
CompositeWeight-based rewiring; Unified gene symbols used in labels where available
Top rewired edges: Ab - Liver
CompositeWeight-based rewiring; labels use Unified_GeneSymbol when available
Top rewired edges: Mz - Liver
CompositeWeight-based rewiring; labels use Unified_GeneSymbol when available
Top rewired edges: Nb - Liver
CompositeWeight-based rewiring; labels use Unified_GeneSymbol when available
Top rewired edges: On - Liver
CompositeWeight-based rewiring; labels use Unified_GeneSymbol when available
Top rewired edges: Pn - Liver
CompositeWeight-based rewiring; labels use Unified_GeneSymbol when available
TF → target
Rewiring score
Unique edge
TRUE

Fig. S31 - Top rewired GENIE3 edges in liver across species. Bar plots ranking the top composite-weighted rewired GENIE3 edges in liver for each of the five species. Bars are coloured red and show rewiring score on the x-axis; gene symbols (unified where available) label the y-axis. Red horizontal lines indicate edges confirmed as unique in motif-supported GENIE3 edges.

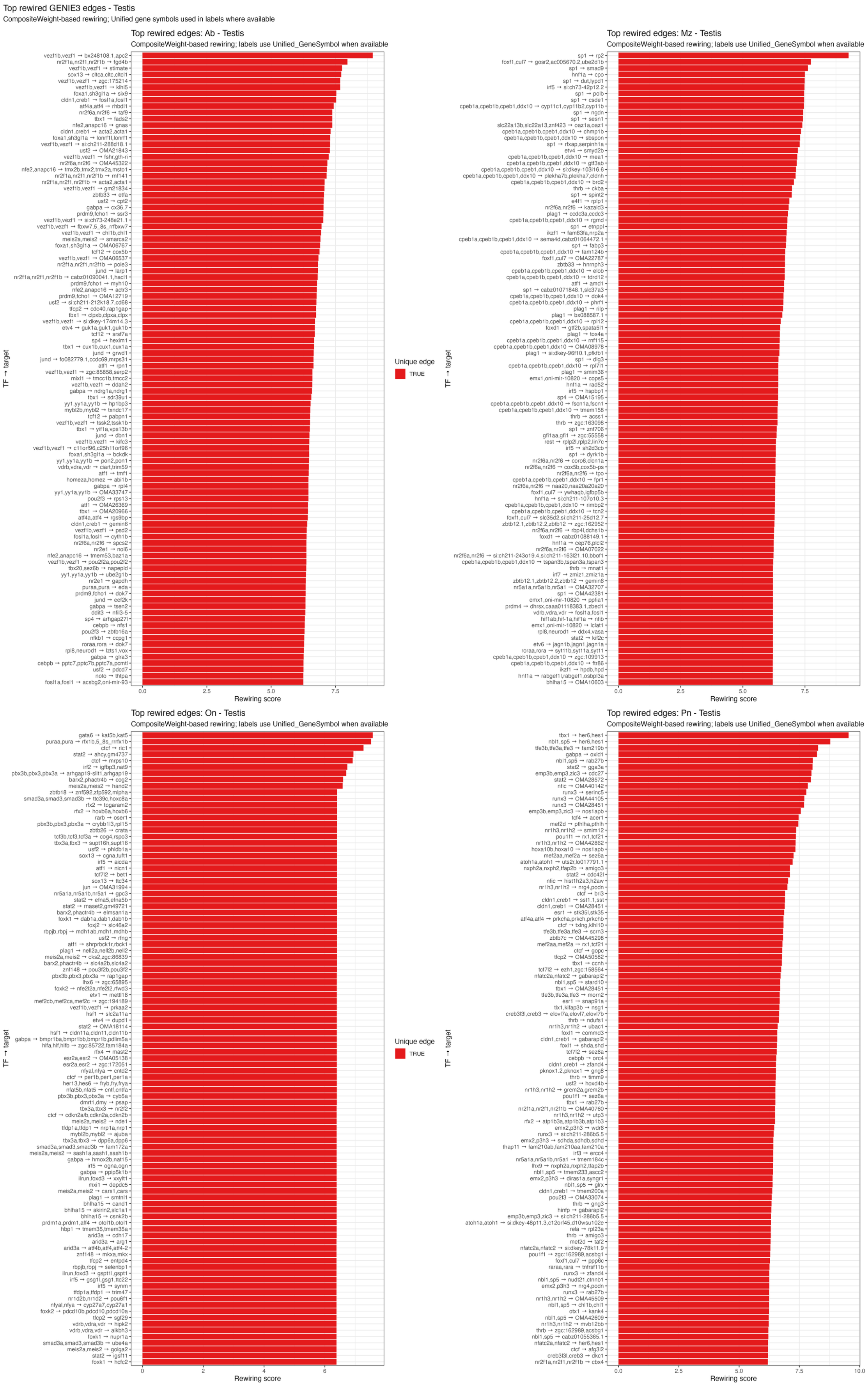

Top rewired GENIE3 edges - Testis
CompositeWeight-based rewiring; Unified gene symbols used in labels where available
Top rewired edges: Ab - Testis
CompositeWeight-based rewiring; labels use Unified_GeneSymbol when available
Top rewired edges: Mz - Testis
CompositeWeight-based rewiring; labels use Unified_GeneSymbol when available
Top rewired edges: On - Testis
CompositeWeight-based rewiring; labels use Unified_GeneSymbol when available
Top rewired edges: Pn - Testis
CompositeWeight-based rewiring; labels use Unified_GeneSymbol when available
TF → target
Rewiring score
Unique edge
TRUE

Fig. S32 - Top rewired GENIE3 edges in testis across species. Bar plots ranking the top composite-weighted rewired GENIE3 edges in testis for of four species. Bars are coloured red and show rewiring score on the x-axis; gene symbols (unified where available) label the y-axis. Red horizontal lines indicate edges confirmed as unique in motif-supported GENIE3 edges.

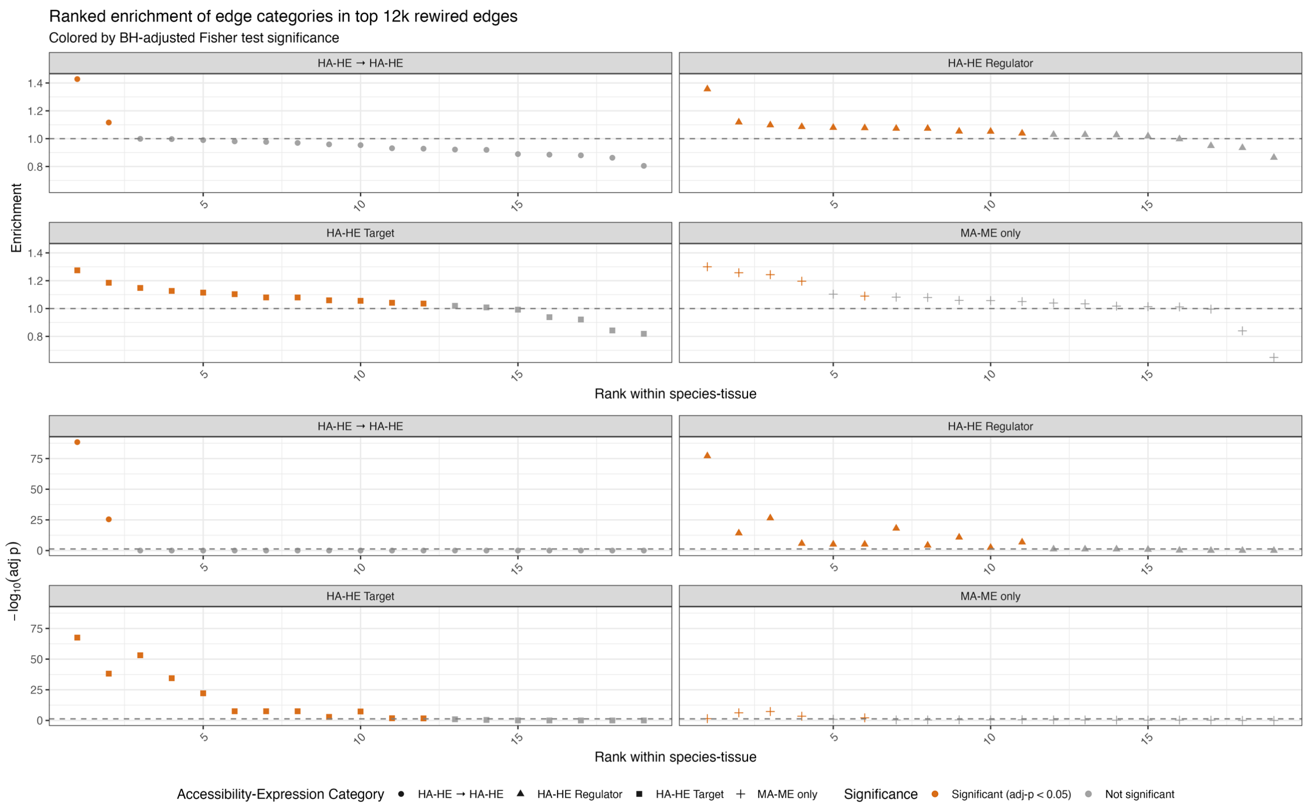

Fig. S33 – Collated ranked enrichment of HA/HE categories in rewired edges. Line plots show BH-adjusted Fisher test enrichment of accessibility-expression categories (HA-HE → HA+HE, HA-HE regulator/target, MA-ME only) among the top 12K rewired edges, ranked by enrichment rank within species-tissue. Points are coloured by significance (orange for adj. $p<0.05$, grey for non-significant) with shapes distinguishing categories (circles for HA-HE → HA+HE, triangles for HA-HE as regulators, square as targets, and cross for MA-ME only). Separate panels for enrichment and their $-\log_{10}$(p-adj).

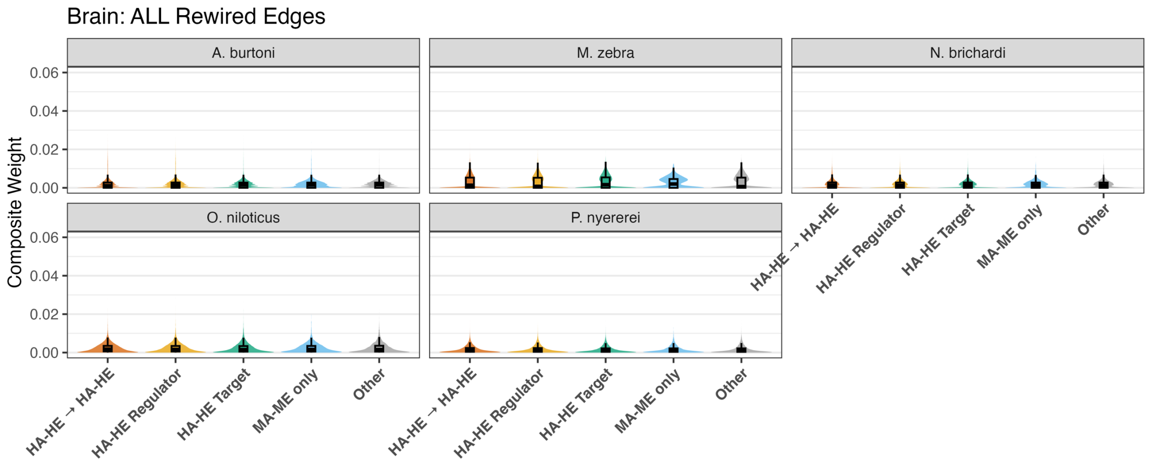

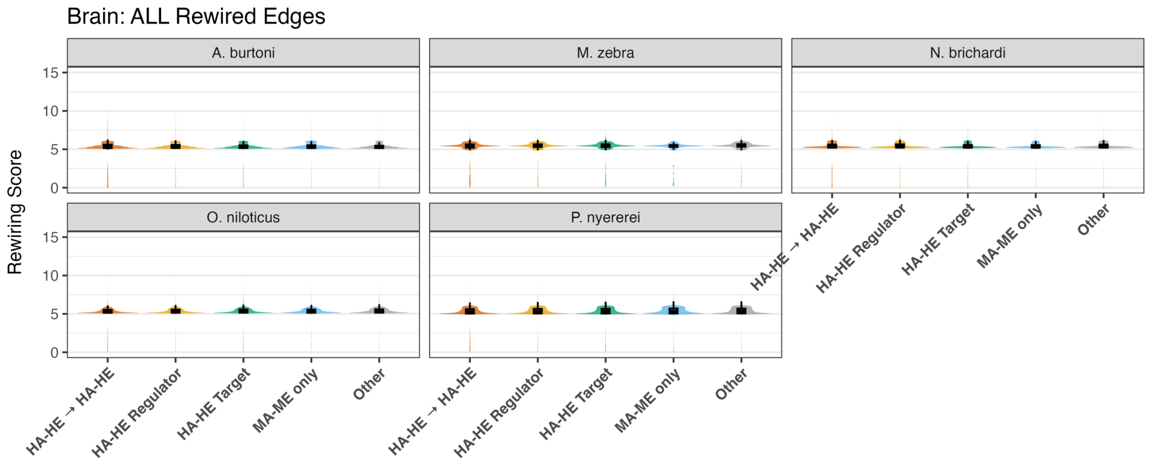

Fig. S34 – Accessibility-Expression category distribution in all rewired edges for forebrain across species. Top row: violin/box plots of composite weight (y-axis) for rewired edges by all accessibility-expression categories (x-axis: HA-HE → HA+HE, HA-HE regulator/target, MA-ME, other) across all five species. Bottom row: violin/box plots of rewiring scores (y-axis) for the same categories and species. All categories coloured distinctly (HA-HE → HA+HE: orange; HA-HE regulator: yellow; HA-HE target: green; MA-ME: blue; other: grey).

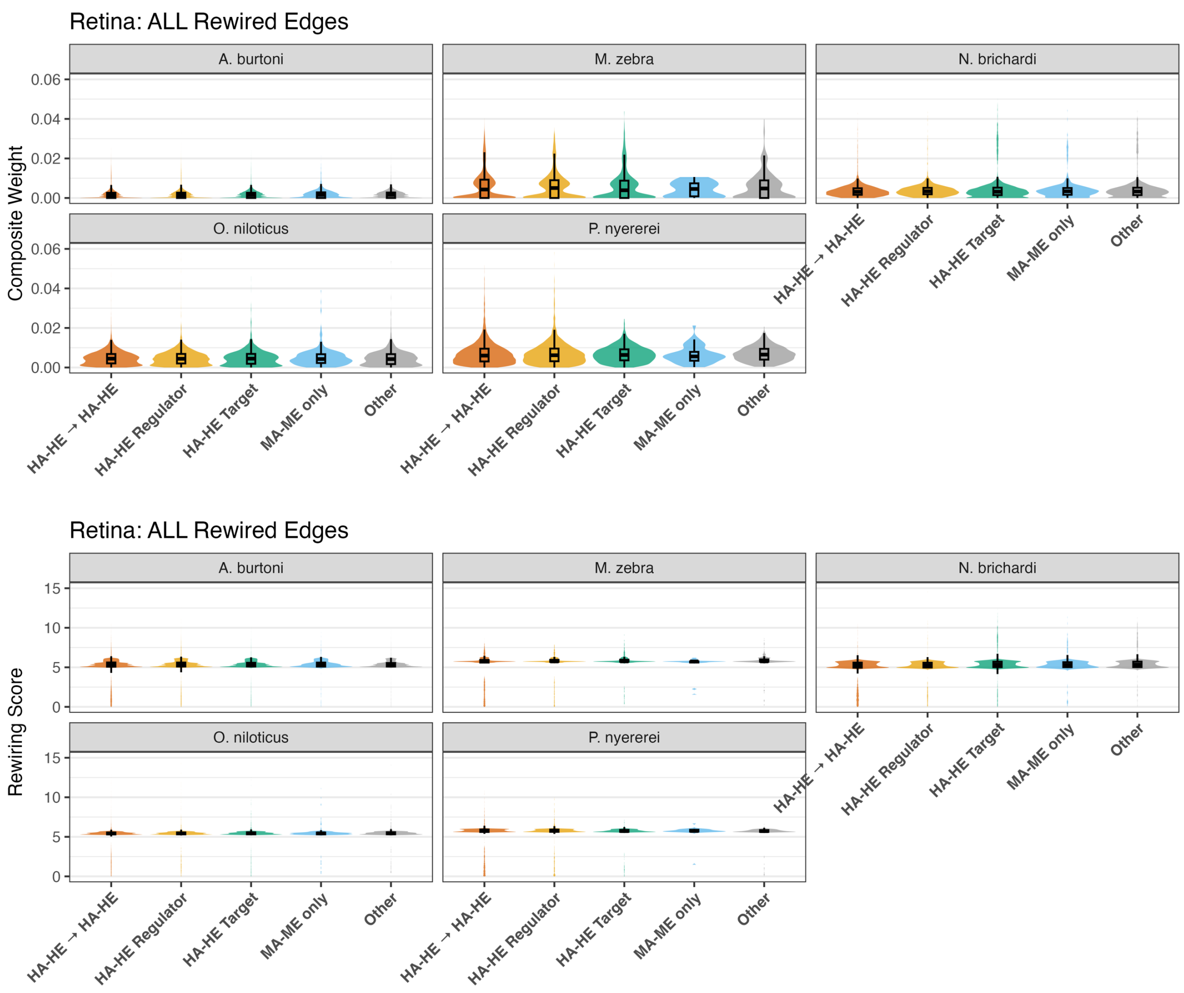

Fig. S35 – Accessibility-Expression category distribution in all rewired edges for retina across species. Top row: violin/box plots of composite weight (y-axis) for rewired edges by all accessibility-expression categories (x-axis: HA-HE → HA+HE, HA-HE regulator/target, MA-ME, other) across all five species. Bottom row: violin/box plots of rewiring scores (y-axis) for the same categories and species. All categories coloured distinctly (HA-HE → HA+HE: orange; HA-HE regulator: yellow; HA-HE target: green; MA-ME: blue; other: grey).

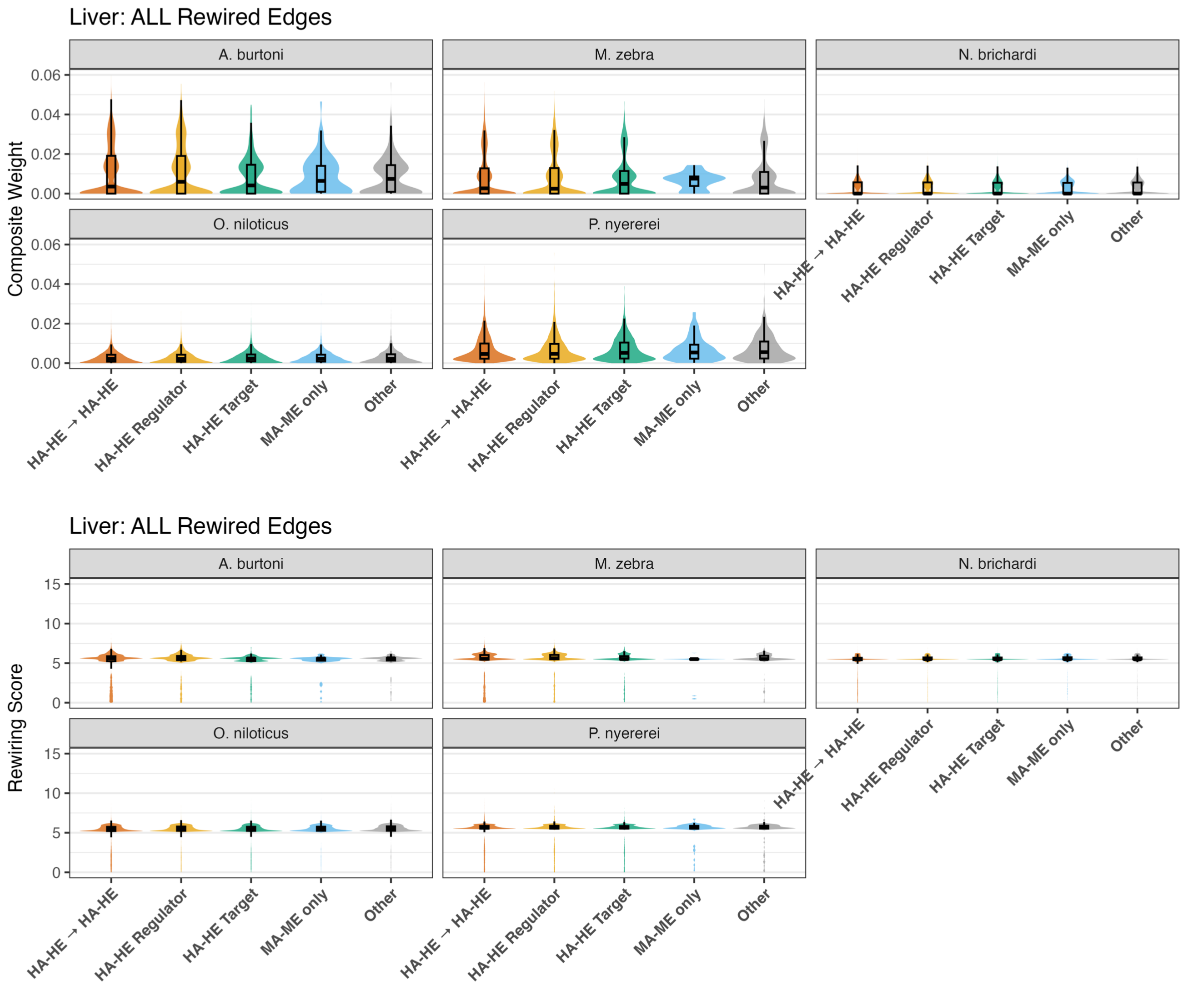

Fig. S36 – Accessibility-Expression category distribution in all rewired edges for liver across species. Top row: violin/box plots of composite weight (y-axis) for rewired edges by all accessibility-expression categories (x-axis: HA-HE → HA+HE, HA-HE regulator/target, MA-ME, other) across all five species. Bottom row: violin/box plots of rewiring scores (y-axis) for the same categories and species. All categories coloured distinctly (HA-HE → HA+HE: orange; HA-HE regulator: yellow; HA-HE target: green; MA-ME: blue; other: grey).

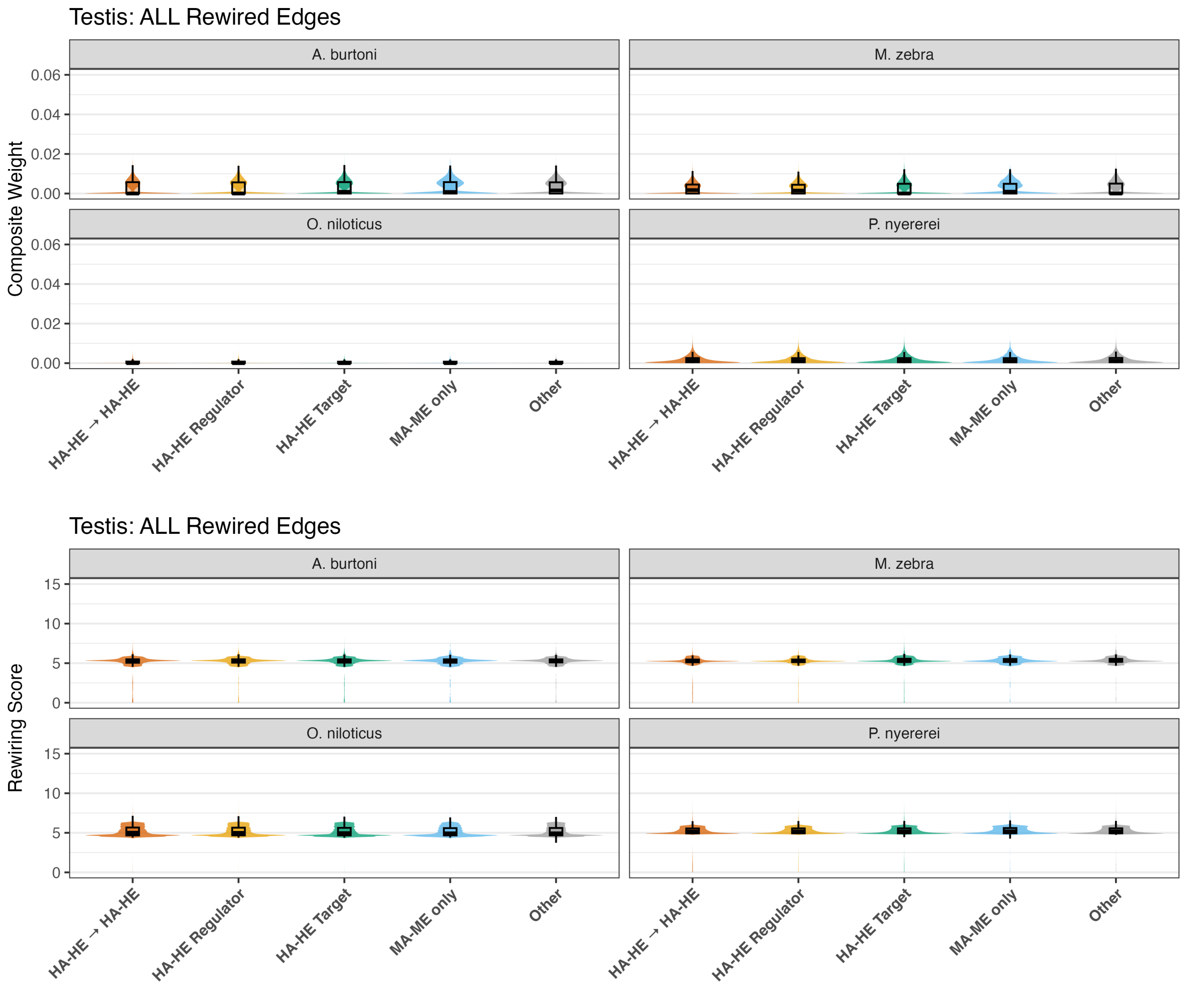

Fig. S37 – Accessibility-Expression category distribution in all rewired edges for testis across species. Top row: violin/box plots of composite weight (y-axis) for rewired edges by all accessibility-expression categories (x-axis: HA-HE → HA+HE, HA-HE regulator/target, MA-ME, other) across four species. Bottom row: violin/box plots of rewiring scores (y-axis) for the same categories and species. All categories coloured distinctly (HA-HE → HA+HE: orange; HA-HE regulator: yellow; HA-HE target: green; MA-ME: blue; other: grey).

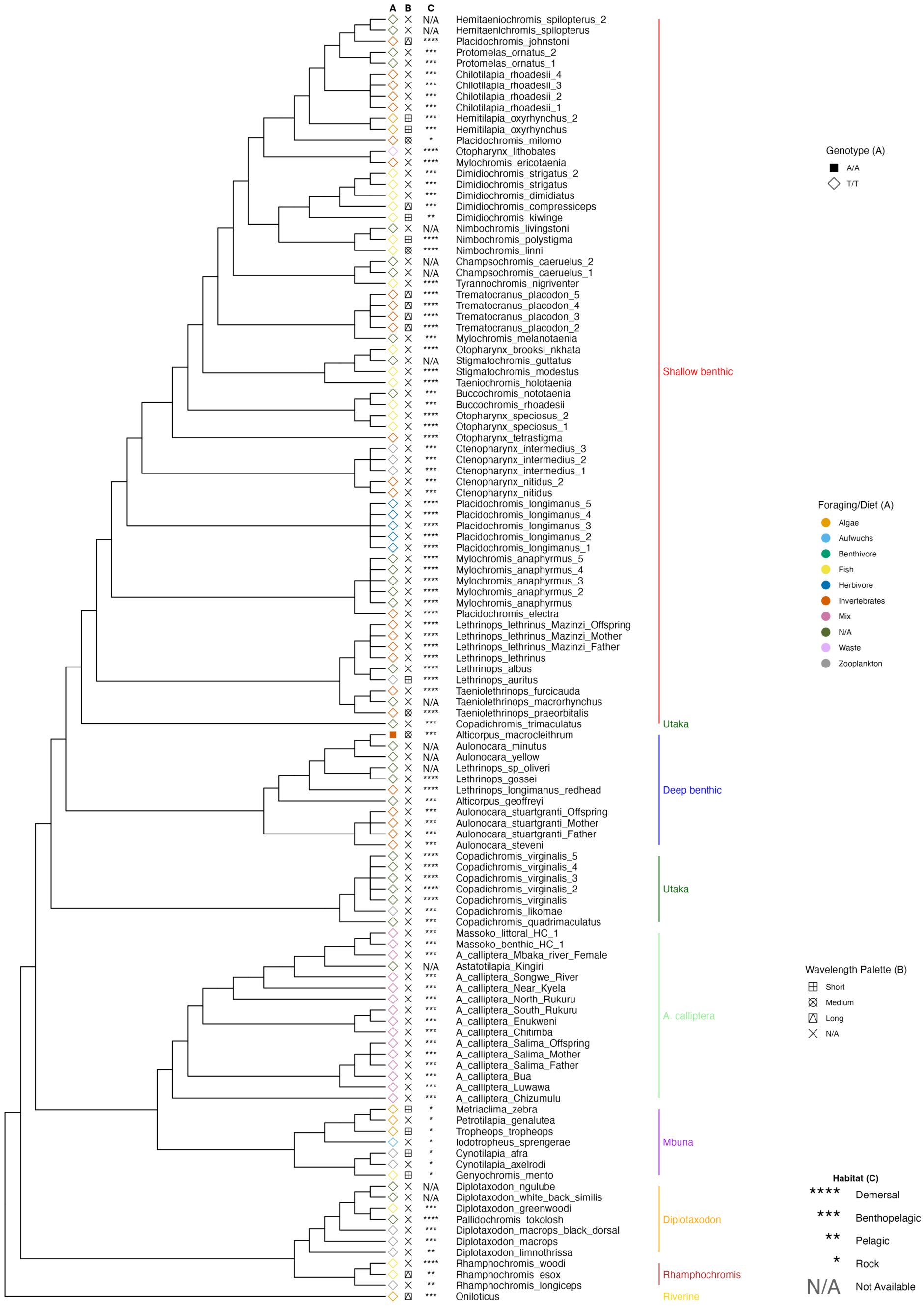

Fig. S38 – SNP genotypes overlapping MAF TFBS in *M. zebra actr1* promoter and other Lake Malawi species. Lake Malawi phylogeny reproduced from published least controversial and all included species ASTRAL phylogeny [34], including *O. niloticus* as an outgroup. Phylogenetic branches labelled with species sample name and clade according to legends (right): (a) species foraging/diet habit (color) [39] and phased SNP genotype (shape) [34,35]; (b) adult opsin wavelength palette utilised [39] and (c) species habitat [39,40].

```
                                              ....:..970....:..980....:..990....:.1000....:.1010....:.1020
O. niloticus actr1 gene promoter           180 ATTATTAGATAAATGTGATAGTTAACTGTGATCTGCCAGCACACCAGCAGAAACGCTGCC
R. 'chilingali' actr1 gene promoter        945 ATTATTTGTTAAATGTGATAGTTAACTGTGATCTGCCAGCAGACCAGCAGAAACGCTGCC
T. 'mauve' actr1 gene promoter             946 ATTATTTGTTAAATGTGATAGTTAACTGTGATCTGCCAGCAGACCAGCAGAAACGCTGCC
A. stuartgranti actr1 gene promoter        837 ATTATTTGTTAAATGTGATAGTTAACTGTGATCTGCCAGCAGACCAGCAGAAACGCTGCC
M. zebra actr1 gene promoter               945 ATTATTTGTTAAATGTGATAGTTAACTGTGATCTGCCAGCAGACCAGCAGAAACGCTGCC
A. calliptera actr1 gene promoter          944 ATTATTTGTTAAATGTGATAGTTAACTGTGATCTGCCAGCAGACCAGCAGAAACGCTGCC
A. macrocleithrum actr1 gene promoter        1 ---------------------------------------------------------GCC

                                              ....:.1030....:.1040....:.1050....:.1060....:.1070....:.1080
O. niloticus actr1 gene promoter           240 GCTCGTTGACAGCGTACAGACATGCTGATTAGCTCTCCGTGCTGCCAGTCAGGATCTCTG
R. 'chilingali' actr1 gene promoter       1005 ACTCGTTGACAGCGTATAGACATGCTGATTGGCTCTCCGTGATGCCAGTCAGGATCTCTG
T. 'mauve' actr1 gene promoter            1006 ACTCGTTGACAGCGTATAGACATGCTGATTGGCTCTCCGTGATGCCAGTCAGGATCTCTG
A. stuartgranti actr1 gene promoter        897 ACTCGTTGACAGCGTATAGACATGCTGATTGGCTCTCCGTGATGCCAGTCAGGATCTCTG
M. zebra actr1 gene promoter              1005 ACTCGTTGACAGCGTATAGACATGCTGATTGGCTCTCCGTGATGCCAGTCAGGATCTCTG
A. calliptera actr1 gene promoter         1004 ACTCGTTGACAGCGTATAGACATGCTGATTGGCTCTCCGTGATGCCAGTCAGGATCTCTG
A. macrocleithrum actr1 gene promoter        4 ACTCGTTGACAGCGTATAGACAAGCTGATTGGCTCTCCGTGATGCCAGTCAGGATCTCTG

                                              ....:.1090....:.1100....:.1110....:.1120....:.1130....:.1140
O. niloticus actr1 gene promoter           300 GAGCTTGATTGGCTGTCTGCACCGTGAGCCCGCCGCGATGCTGTGGTTGTTGTGGTCGGG
R. 'chilingali' actr1 gene promoter       1065 GAGCTTGATTGGCTGTCTGCACCGTGAGCCCGCCTCGGTGCTGTGGTTGTTGTGGACGGG
T. 'mauve' actr1 gene promoter            1066 GAGCTTGATTGGCTGTCTGCACCGTGAGCCCGCCTCGGTGCTGTGGTTGTTGTGGACGGG
A. stuartgranti actr1 gene promoter        957 GAGCTTGATTGGCTGTCTGCACCGTGAGCCCGCCTCGGTGCTGTGGTTGTTGTGGACGGG
M. zebra actr1 gene promoter              1065 GAGCTTGATTGGCTGTCTGCACCGTGAGCCCGCCTCGGTGCTGTGGTTGTTGTGGACGGG
A. calliptera actr1 gene promoter         1064 GAGCTTGATTGGCTGTCTGCACCGTGAGCCCGCCTCGGTGCTGTGGTTGTTGTGGACGGG
A. macrocleithrum actr1 gene promoter       64 GAGCTTGATTGGCTGTCTGCACCGTGAGCCCGCCTCGGTGCTGTGGTTGTTGTGGACGG-

                                              ....:.1150....:.1160....:.1170....:.1180....:.1190....:.1200
O. niloticus actr1 gene promoter           360 GCGGCAGCAGCAGCGGCAGAGGAGAGTCGACAGGCGTAGAGCGGAGCGGCTAACCGGCGC
R. 'chilingali' actr1 gene promoter       1125 GCG---GCAGCAGCGGCAGAGGAGCGTCGACAGGCGTAGAGCGGAGCTGCTAACCGGCGC
T. 'mauve' actr1 gene promoter            1126 GCG---GCAGCAGCGGCAGAGGAGCGTCGACAGGCGTAGAGCGGAGCTGCTAACCGGCGC
A. stuartgranti actr1 gene promoter       1017 GCG---GCAGCAGCGGCAGAGGAGCGTCGACAGGCGTAGAGCGGAGCTGCTAACCGGCGC
M. zebra actr1 gene promoter              1125 GCG---GCAGCAGCGGCAGAGGAGCGTCGACAGGCGTAGAGCGGAGCTGCTAACCGGCGC
A. calliptera actr1 gene promoter         1124 GCG---GCAGCAGCGGCAGAGGACCGTCGACAGGCGTAGAGCGGAGCTGCTAACCGGCGC
A. macrocleithrum actr1 gene promoter      123 ------------------------------------------------------------

                                              ....:.1210....:.1220....:.1230....:.1240....:.1250....:.1260
O. niloticus actr1 gene promoter           420 TGATTTTTACATGATTAAAACACTAAACGGGAACGAAGACTCACGCGGATCGATAGAAAC
R. 'chilingali' actr1 gene promoter       1182 TGATTTTTACATGATTAAAACACTAAACGGGCACGAAGACTCACGCGGATCGATAGAAAC
T. 'mauve' actr1 gene promoter            1183 TGATTTTTACATGATTAAAACACTAAACGGGCACGAAGACTCACGTGGATCGATAGAAAC
A. stuartgranti actr1 gene promoter       1074 TGATTTTTACATGATTAAAACACTAAACGGGCACGAAGACTCACGTGGATCGATAGAAAC
M. zebra actr1 gene promoter              1182 TGATTTTTACATGATTAAAACACTAAACGGGCACGAAGACTCACGCGGATCGATAGAAAC
A. calliptera actr1 gene promoter         1181 TGATTTTTACATGATTAAAACACTAAACGGGCACGAAGACTCACGCGGATCGATAGAAAC
A. macrocleithrum actr1 gene promoter      123 ------------------------------------------------------------

                                              ....:.1270....:.1280...
O. niloticus actr1 gene promoter           480 ACACCTGAAGCGCACGTGCGGTC > O. niloticus actr1 gene
R. 'chilingali' actr1 gene promoter       1242 ACACCTGAAGCGCACGTGCGGTC
T. 'mauve' actr1 gene promoter            1243 ACACCTGAAGCGCACGTGCGGTC
A. stuartgranti actr1 gene promoter       1134 ACACCTGAAGCGCACGTGCGGTC
M. zebra actr1 gene promoter              1242 ACACCTGAAGCGCACGTGCGGTC > M. zebra actr1 gene
A. calliptera actr1 gene promoter         1241 ACACCTGAAGCGCACGTGCGGTC
A. macrocleithrum actr1 gene promoter      123 -----------------------
```

MAF_HUMAN.H11MO.1.B TFBS Motif

T Segregating TFBS variant

Fig. S39 – Alignment of actr1 gene promoter region in Lake Malawi cichlids, *O. niloticus*, and *A. calliptera*. Predicted MAF motifs (blue shading) and polymorphic sites (orange text) in *O. niloticus* and *M. zebra actr1* gene promoter region. Orthologous alignment with other cichlid genomes and consensus *A. macrocleithrum* reads [34,35] shown with light grey or white shading indicating polymorphic sites.

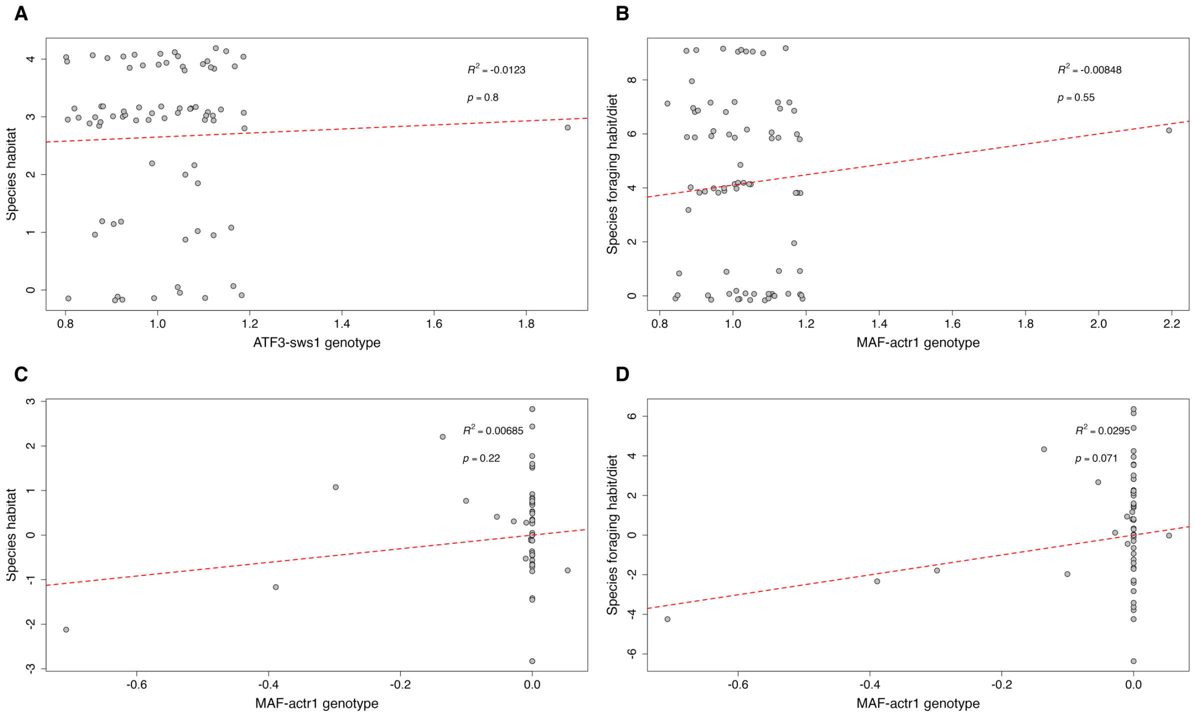

Fig. S40 – Phylogenetic independent contrast analysis of MAF-actr1 target site genotypes of Lake Malawi species against their ecology. Phylogenetic independent scatterplots of MAF-actr1 TFBS genotypes (1=T/T, 2=A/A) in 119 Lake Malawi individuals (73 species) against their respective (a) habitat (0=N/A, 1=Rock, 2=Pelagic, 3=Benthopelagic, 4=Demersal); and (b) foraging habit/diet (0=N/A, 1=Algae, 2=Aufwuchs, 3=Benthivore, 4=Fish, 5=Herbivore, 6=Invertebrates, 7=Mix, 8=Waste, 9=Zooplankton). Corresponding scatterplots of Lake Malawi ASTRAL phylogeny 3 and regression model fitted to MAF-actr1 TFBS genotypes (1=T/T, 2=A/A) of 119 Lake Malawi individuals (73 species) against their respective (c) habitat (0=N/A, 1=Rock, 2=Pelagic, 3=Benthopelagic, 4=Demersal); and (d) foraging habit/diet (0=N/A, 1=Algae, 2=Aufwuchs, 3=Benthivore, 4=Fish, 5=Herbivore, 6=Invertebrates, 7=Mix, 8=Waste, 9=Zooplankton). All data points used as per Supplementary Fig. S24, with overlapping coordinates 'jittered' around their respective point to highlight density. Adjusted r2 and p-value of each regression line shown in top right of each plot.

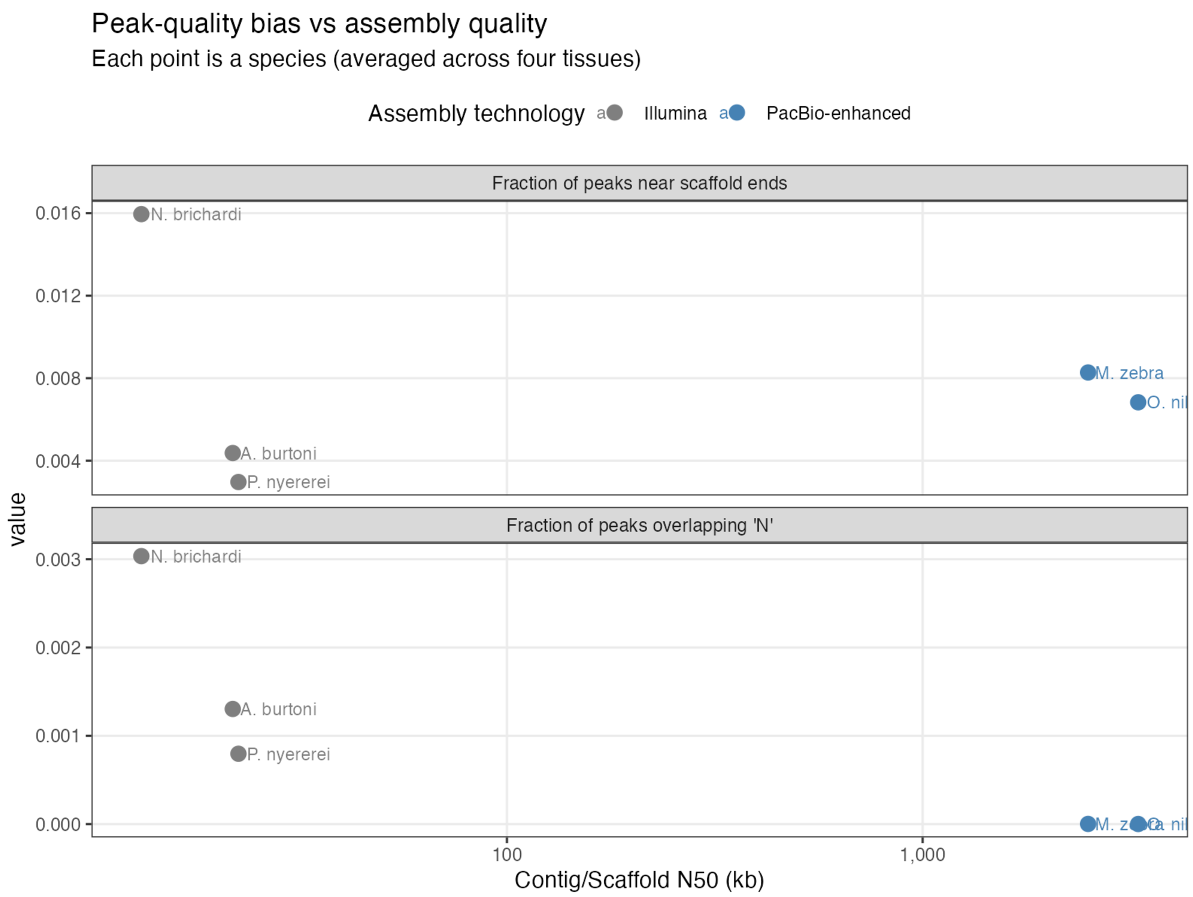

Fig. S41 – Raw relationship between assembly contiguity and peak bias. Species-level mean fractions of ATAC peaks overlapping at least 10% of gap-masked sequence (N/n) or lying within 100 bp of scaffold ends, plotted against assembly contiguity (N50, kb) for the five cichlid species. For each species, peak metrics were averaged across the four tissues examined. Points are coloured by assembly technology, with Illumina-only assemblies in grey and PacBio-enhanced assemblies in blue. The plot shows the unadjusted association between assembly quality and peak placement relative to gap-rich or terminal scaffold regions.

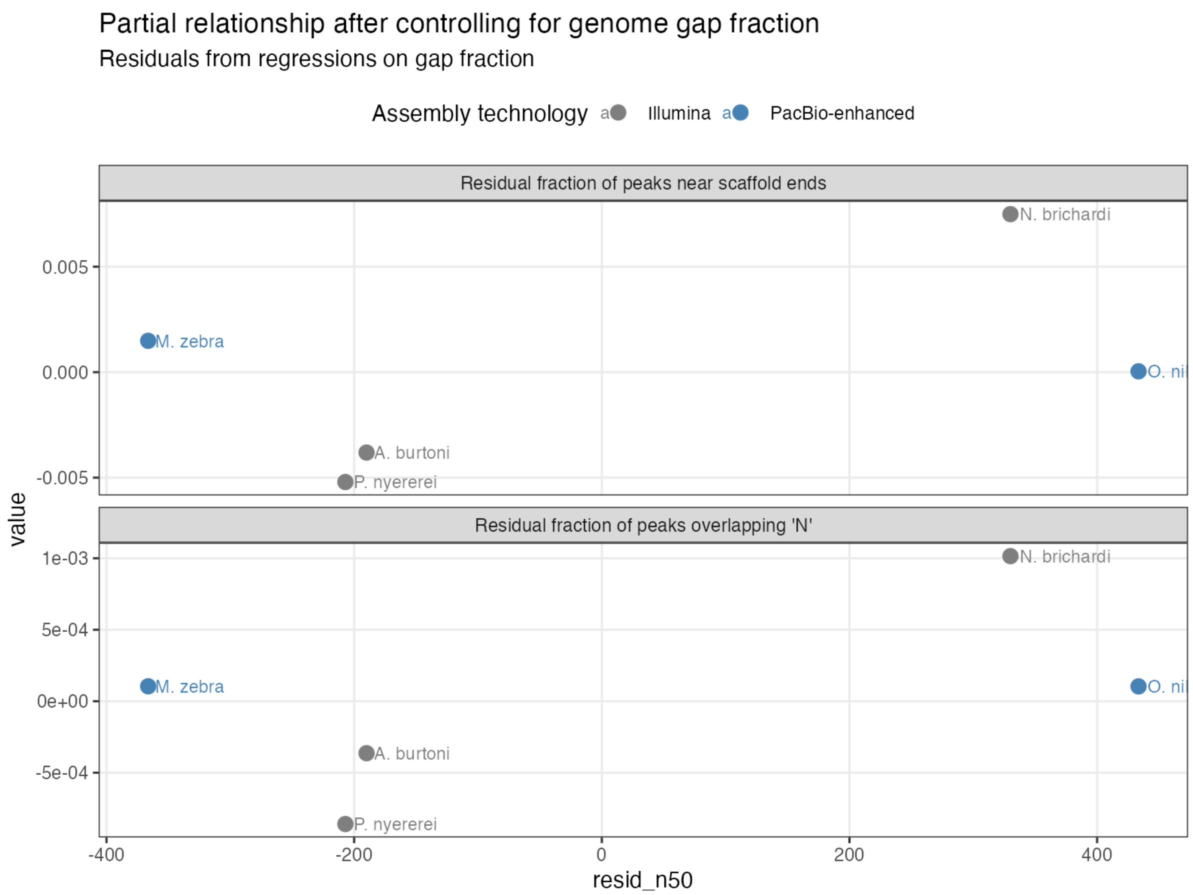

Fig. S42 – Gap-adjusted relationship between assembly contiguity and peak bias. Residual species-level mean fractions of ATAC peaks overlapping at least 10% of gap-masked sequence (N/n) or lying within 100 bp of scaffold ends, plotted against residual assembly contiguity after controlling for genome-wide gap fraction. Residuals were obtained from regressions on genome gap fraction, calculated as the proportion of bases in the genome FASTA encoded as N/n. Peak metrics were averaged across the four tissues for each species, and points are coloured by assembly technology as in Fig. S41. This adjusted analysis assesses whether the apparent association between contiguity and peak placement persists after accounting for species-wide assembly gap burden.

<u>**References**</u>

1. Kasper, C., Hebert, F.O., Aubin-Horth, N. & Taborsky, B. Divergent brain gene expression profiles between alternative behavioural helper types in a cooperative breeder. *Molecular Ecology* **27**, 4136–4151 (2018).
2. Nishiguchi, K.M., Aoki, M., Nakazawa, T. & Abe, T. Macular degeneration as a common cause of visual loss in spinocerebellar ataxia type 1 (SCA1) patients. *Ophthalmic Genetics* **40**, 49–53 (2019).
3. Mehta, T.K. *et al.* Evolution of miRNA binding sites and regulatory networks in cichlids. *bioRxiv*, 2021.12.14.472604–2021.12.14.472604 (2021).
4. Mehta, T. *et al.* Evolution of regulatory networks associated with traits under selection in cichlids. *Genome Biol* **22**, 25–25 (2021).
5. Wang, X., Kiyokawa, H., Dennewitz, M.B. & Costa, R.H. The Forkhead Box m1b transcription factor is essential for hepatocyte DNA replication and mitosis during mouse liver regeneration. *Proceedings of the National Academy of Sciences* **99**, 16881–16886 (2002).
6. Soler-Llavina, G.J., Fuccillo, M.V., Ko, J., Südhof, T.C. & Malenka, R.C. The neurexin ligands, neuroligins and leucine-rich repeat transmembrane proteins, perform convergent and divergent synaptic functions in vivo. *Proceedings of the National Academy of Sciences* **108**, 16502–16509 (2011).
7. Tissir, F. & Goffinet, A.M. Reelin and brain development. *Nat Rev Neurosci* **4**, 496–505 (2003).
8. Govindarajan, V., Ito, M., Makarenkova, H.P., Lang, R.A. & Overbeek, P.A. Endogenous and ectopic gland induction by FGF-10. *Dev Biol* **225**, 188–200 (2000).
9. Spady, T. *et al.* Evolution of the cichlid visual palette through ontogenetic subfunctionalization of the opsin gene arrays. *Mol Biol Evol* **23**, 1538–47 (2006).
10. Khanam, A. *et al.* Blockade of Neutrophil's Chemokine Receptors CXCR1/2 Abrogate Liver Damage in Acute-on-Chronic Liver Failure. *Front Immunol* **8**, 464 (2017).
11. Zaldivar, M.M. *et al.* The chemokine receptor CXCR3 limits injury after acute toxic liver damage. *Lab Invest* **92**, 724–34 (2012).
12. Juma, A.R. *et al.* PLAG1 deficiency impairs spermatogenesis and sperm motility in mice. *Sci Rep* **7**, 5317 (2017).
13. Paigen, K. & Petkov, P.M. PRDM9 and Its Role in Genetic Recombination. *Trends Genet* **34**, 291–300 (2018).
14. Pollard, K., Hubisz, M.J., Rosenbloom, K.R. & Siepel, A. Detection of nonneutral substitution rates on mammalian phylogenies. *Genome Res* **20**, 110–21 (2010).
15. Mehta, T. *et al.* Evolution of miRNA-binding sites and regulatory networks in cichlids. *MBE* **39**, 146 (2022).
16. Brawand, D. *et al.* The genomic substrate for adaptive radiation in African cichlid fish. *Nature* **2**, 17 (2014).
17. Conte, M.A., Gammerdinger, W.J., Bartie, K.L., Penman, D.J. & Kocher, T.D. A high quality assembly of the Nile Tilapia (Oreochromis niloticus) genome reveals the structure of two sex determination regions. *BMC Genomics* **18**, 341–341 (2017).
18. Conte, M.A. *et al.* Chromosome-scale assemblies reveal the structural evolution of African cichlid genomes. *GigaScience* **8**, giz030–giz030 (2019).

19. Benjamini, Y. & Hochberg, Y. Benjamini Y, Hochberg Y. Controlling the false discovery rate: a practical and powerful approach to multiple testing. *Journal of the Royal Statistical Society B* **57**, 289–300 (1995).
20. Hofmann, H.A. Functional genomics of neural and behavioral plasticity. *J Neurobiol* **54**, 272–82 (2003).
21. Pistocchi, A. *et al.* Crucial role of zebrafish prox1 in hypothalamic catecholaminergic neurons development. *BMC Developmental Biology* **8**, 27 (2008).
22. Acampora, D., Avantaggiato, V., Tuorto, F. & Simeone, A. Genetic control of brain morphogenesis through Otx gene dosage requirement. *Development* **124**, 3639–3650 (1997).
23. He, Z., Agostini, M., Liu, H., Melino, G. & Simon, H.U. p73 regulates basal and starvation-induced liver metabolism in vivo. *Oncotarget* **6**, 33178–90 (2015).
24. Lee, J.M., Lee, D.J., Bae, S.C. & Jung, H.S. Abnormal liver differentiation and excessive angiogenesis in mice lacking Runx3. *Histochem Cell Biol* **139**, 751–8 (2013).
25. Eozenou, C. *et al.* The TALE homeodomain of PBX1 is involved in human primary testis-determination. *Hum Mutat* **40**, 1071–1076 (2019).
26. Ile, K.E. *et al.* Zebrafish class 1 phosphatidylinositol transfer proteins: PITPbeta and double cone cell outer segment integrity in retina. *Traffic* **11**, 1151–67 (2010).
27. Lee, C.S., Friedman, J.R., Fulmer, J.T. & Kaestner, K.H. The initiation of liver development is dependent on Foxa transcription factors. *Nature* **435**, 944–947 (2005).
28. Gusmao, E.G., Allhoff, M., Zenke, M. & Costa, I.G. Analysis of computational footprinting methods for DNase sequencing experiments. *Nat Methods* **13**, 303–9 (2016).
29. Mehta, T.K. *et al.* Chromatin accessibility in gill tissue identifies candidate genes and loci associated with aquaculture relevant traits in tilapia. *Genomics* **115**, 110633 (2023).
30. Carleton, K. Cichlid fish visual systems: mechanisms of spectral tuning. *Integr zool* **4**, 75–86 (2009).
31. Hahn, C., Genner, M.J., Turner, G.F. & Joyce, D.A. The genomic basis of cichlid fish adaptation within the deepwater “twilight zone” of Lake Malawi. *Evolution Letters* **1**, 184–198 (2017).
32. Anand, D., Agrawal, S.A., Slavotinek, A. & Lachke, S.A. Mutation update of transcription factor genes FOXE3, HSF4, MAF, and PITX3 causing cataracts and other developmental ocular defects. *Human Mutation* **39**, 471–494 (2018).
33. Snoeks, J., Carvalho, G.R. & Project, S.G.L.M.N.B.C. *The cichlid diversity of Lake Malawi/Nyasa/Niassa : identification, distribution, and taxonomy*, 360 p. : ill. (some col.), maps (some col.) (Cichlid Press, El Paso, Tex, 2004).
34. Malinsky, M. *et al.* Whole-genome sequences of Malawi cichlids reveal multiple radiations interconnected by gene flow. *Nature Ecol & Evol* **2**, 1940–1955 (2018).
35. Blumer, L.M. *et al.* Introgression dynamics of sex-linked chromosomal inversions shape the Malawi cichlid radiation. *Science* **388**, eadr9961 (2025).

36. Hosseini, R., Balci, A.T., Kostka, D., Clark, N. & Chikina, M. Ultra-fast variant effect prediction using biophysical transcription factor binding models. *bioRxiv*, 2024.06.26.600873 (2025).
37. Felsenstein, J. Phylogenies and the Comparative Method. *The American Naturalist* **125**, 1–15 (1985).
38. Li, Z. *et al.* Identification of transcription factor binding sites using ATAC-seq. *Genome Biology* **20**, 45–45 (2019).
39. Hofmann, C.M. *et al.* The eyes have it: regulatory and structural changes both underlie cichlid visual pigment diversity. *PLoS biology* **7**, e1000266–e1000266 (2009).
40. Froese, R. & Pauly, D. Fishbase. in *FishBase* (2017).
